# Early biomarkers in the presymptomatic phase of cognitive impairment: changes in the endocannabinoidome and serotonergic pathways in Alzheimer's-prone mice after mTBI

**DOI:** 10.1186/s40478-024-01820-0

**Published:** 2024-07-12

**Authors:** Francesca Guida, Monica Iannotta, Anna Lauritano, Rosmara Infantino, Emanuela Salviati, Roberta Verde, Livio Luongo, Eduardo Maria Sommella, Fabio Arturo Iannotti, Pietro Campiglia, Sabatino Maione, Vincenzo Di Marzo, Fabiana Piscitelli

**Affiliations:** 1 Pharmacology Division, Department of Experimental Medicine, University of Campania “L. Vanvitelli”, Naples, Italy; 2 Endocannabinoid Research Group, Institute of Biomolecular Chemistry (ICB), National Research Council (CNR), Pozzuoli, (NA) Italy; 3 Dipartimento di Farmacia, Università Degli Studi di Salerno, Fisciano, (SA) Italy; 4 Institut Universitaire de Cardiologie et de Pneumologie de Québec and Institut sur la Nutrition et les Aliments Fonctionnels, Centre NUTRISS, Université Laval, Quebec City, Canada

**Keywords:** Traumatic brain injury, Alzheimer’s disease, APP-SWE mice, Dementia, Endocannabinoidome, Serotonin

## Abstract

**Background:**

Despite extensive studies on the neurobiological correlates of traumatic brain injury (TBI), little is known about its molecular determinants on long-term consequences, such as dementia and Alzheimer’s disease (AD).

**Methods:**

Here, we carried out behavioural studies and an extensive biomolecular analysis, including inflammatory cytokines, gene expression and the combination of LC-HRMS and MALDI-MS Imaging to elucidate the targeted metabolomics and lipidomics spatiotemporal alterations of brains from wild-type and APP-SWE mice, a genetic model of AD, at the presymptomatic stage, subjected to mild TBI.

**Results:**

We found that brain injury does not affect cognitive performance in APP-SWE mice. However, we detected an increase of key hallmarks of AD, including Aβ_1-42_ levels and BACE1 expression, in the cortices of traumatized transgenic mice. Moreover, significant changes in the expanded endocannabinoid (eCB) system, or endocannabinoidome (eCBome), occurred, including increased levels of the endocannabinoid 2-AG in APP-SWE mice in both the cortex and hippocampus, and *N*-acylserotonins, detected for the first time in the brain. The gene expression of enzymes for the biosynthesis and inactivation of eCBs and eCB-like mediators, and some of their main molecular targets, also underwent significant changes. We also identified the formation of heteromers between cannabinoid 1 (CB_1_) and serotonergic 2A (5HT_2A_) receptors, whose levels increased in the cortex of APP-SWE mTBI mice, possibly contributing to the exacerbated pathophysiology of AD induced by the trauma.

**Conclusions:**

Mild TBI induces biochemical changes in AD genetically predisposed mice and the eCBome may play a role in the pathogenetic link between brain injury and neurodegenerative disorders also by interacting with the serotonergic system.

**Supplementary Information:**

The online version contains supplementary material available at 10.1186/s40478-024-01820-0.

## Background

Traumatic brain injury (TBI) is a major and debilitating disease affecting millions of people around the world. It is considered a global priority not only for health issues, but also for the burden to health-care systems and economies [1]. TBI is defined as an alteration of brain function induced by external mechanical forces and its severity can range along a continuum from mild to severe [2]. Despite being the less severe form of TBI, mild-TBI (mTBI) is the most common. It is characterised by an initial neuroinflammation, followed by the late appearance of neurological debilitating symptoms and cognitive impairments occurring in approximately 15% of individuals [3]. An epidemiological link between a history of TBI and the development of Alzheimer's disease (AD) and related dementias later in life exists [4]. In particular, amyloid-β (Aβ) plaques, a major hallmark of AD, have been shown in post-mortem brains of patients with a history of TBI, suggesting that this condition may predispose to AD development [5]. However, there is yet no consensus for this association and the underlying molecular mechanisms remain underexplored.

Accumulating evidence has shown that the endocannabinoid (eCB) signalling system, and its expanded version, the “endocannabinoidome” (eCBome), play a key role in numerous physiological and pathological conditions, including neuroprotection [6, 7]. In fact, the molecular targets of endocannabinoids are expressed and functionally activated in strategic nuclei of the brain where they regulate the secondary events linked with trauma [8], thus offering exciting and attractive targets for novel therapeutic drugs [7]. At the turn of the century the eCB system included: (1) two G-protein coupled receptors (GPCRs), known as cannabinoid receptors (CB_1_ and CB_2_) through which the psychoactive component of cannabis, Δ9-tetrahydrocannabinol (THC), also acts; (2) two potent endogenous agonists of cannabinoid receptors, *N*-arachidonoyl-ethanolamine (anandamide, AEA) and 2-arachidonoyl-glycerol (2-AG), named endocannabinoids (eCBs); (3) the enzymes that regulate endocannabinoid biosynthesis (NAPE-PLD, ABHD4, GDE1 for AEA, and DAGLα and DAGLβ for 2-AG) and degradation (FAAH for AEA, and MAGL, ABHD6, ABHD12 and FAAH for 2-AG) [9]. However, other endogenous AEA and 2-AG congeners and analogues, including other usually cannabinoid receptor-inactive or weakly active *N*-acyl-ethanolamines (NAEs, like AEA) and monoacylglycerols (MAGs, like 2-AG), but also *N*-acyl-aminoacids, *N*-acyl-dopamines and serotonines, have been identified, and their biosynthesis, inactivation and function through non-cannabinoid receptors investigated [10].

Using the weight-drop method, we have previously demonstrated that mTBI induces an increase of pro-inflammatory markers, including IL-1β [11], and causes substantial changes in eCB signaling, with a prolonged 2-AG level increase in brain areas regulating memory and cognition, such as the hippocampus, prefrontal cortex, insula and hypothalamus [12].

Here, we investigated the hypothesis that the eCBome, including the eCB system, is involved in the pathogenetic link between TBI and neurodegenerative disorders. Specifically, we hypothesised that the brain trauma may predispose to a cognitive decline, by exacerbating and/or accelerating pathological and cognitive damage in AD. In this context, we carried out an extensive investigation of the neurobehavioral and biomolecular landscapes in APP-SWE mice at a pre-symptomatic stage subjected to mTBI, using a multidisciplinary approach, including: (1) behavioural testing to characterise the long term cognitive sequelae of the brain injury in WT and APP-SWE mice; (2) biochemical analysis to measure brain (cortex and hippocampus) Aβ_1−42_ levels and the key circulating inflammatory cytokines; (3) omics analysis [transcriptomics, targeted metabolomics by Matrix Assisted Laser Desorption Ionization Mass Spectrometry Imaging (MALDI-MSI) and lipidomics by liquid chromatography coupled to high resolution mass spectrometry (LC-HRMS)] to investigate the possible alterations of the eCBome at the brain level.

## Methods

### Experimental design

**Fig. 1:**
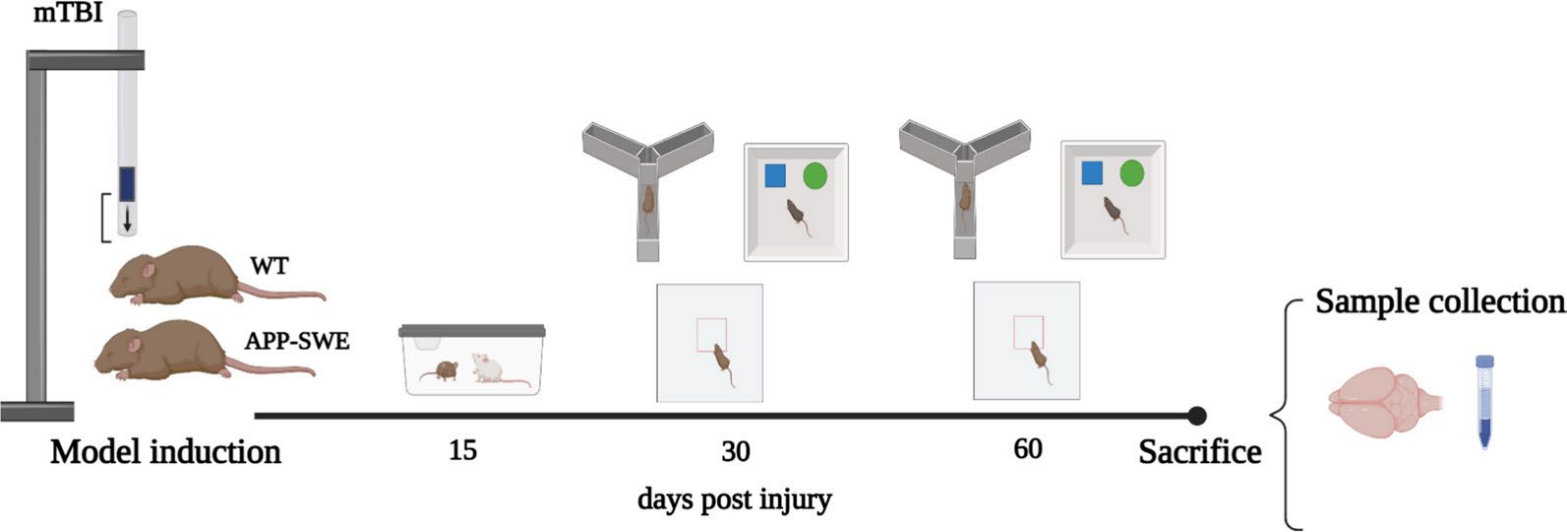
Timeline of the experimental design

Mice were divided into four experimental groups: WT Sham and mTBI or APP-SWE Sham and mTBI. After 1 week of acclimation, animals were submitted to the trauma. Based on our previous studies [11, 26], behavioural evaluations were performed at different time points (aggressive behaviour at day 15 after surgery, whereas the remaining evaluations were performed at 30 and 60 days). By assigning the animals to distinct behavioural tasks, the testing was arranged to prevent the impact of prior testing experiences. The day after the last experiments (60 days), blood sampling and beheading were performed for biochemical evaluations. The heads were immediately immersed in liquid nitrogen for 6 s. The brains were then removed and the regions of interest (hippocampus and cortex) dissected within 20 s on an ice surface. A timeline of the workflow is shown in Fig. 1.

### Animals

A total number of 100 APP-SWE, which carry a transgene coding for the 695-amino acid isoform of human Alzheimer β-amyloid (Aβ) precursor protein carrying the Swedish mutation [B6;SJL-Tg(APP-SWE) Rd1-tested; 1349Rrd1-wt/wt and 1349Rrd1- tg/wt (WT)] mice (10–17 weeks) obtained from Taconic Biosciences were housed under controlled illumination (12 h light/dark cycle; light on at 6:00 am) and environmental conditions (ambient temperature 20–22 °C, humidity 55%-60%) for 1 week before the commencement of experiments. Mice were housed one per cage, as recommended by the manufacturing company, with chow and tap water available ad libitum. The experimental procedures were approved by the Animal Ethics Committee of University of Campania "Vanvitelli" Naples. Italian (D.L. 116/92) and European Commission (O.J. of E.C. L358/1 18/12/86) laws on the protection of laboratory animals were observed in the care of the animals [MOH project 1185/2020]. All efforts were made to minimize animal suffering and to reduce the number of animals used.

### Mild TBI induction

Experimental mTBI was performed using a weight-drop device developed in our laboratory [11]. Mice were anesthetized with an intraperitoneal injection of Tribromoethanol (250 mg/kg) and placed in a prone position on a spongy support. After a midline longitudinal incision, the skull was exposed to locate the area of impact and placed under a metal tube device where the opening was positioned directly over the animal's head. The injury was induced by dropping a cylindrical metal weight (50 g), through a vertical metal guide tube from a height of 20 cm. The point of impact was between the anterior coronal suture (bregma) and posterior coronal suture (lambda) along the midline. Immediately following injury, the skin was closed with surgical wound clips and mice were placed back in their cages to allow for recovery from the anesthesia and mTBI. Sham mice were submitted to the same procedure as described for mTBI, but without the release of the weight.

### Resident-intruder

At 15 days after mTBI or sham surgery, mice were tested for aggressive behavior using the resident intruder test. Mice were individually housed for 1 week in plexiglas cages to establish a home territory and to increase the aggression of the resident experimental mice. To begin, food containers were removed, and an intruder mouse of the same gender was placed in a resident home cage and resident–intruder interactions were analyzed for 10 min. The aggressive behavior of resident socially-isolated mice was characterized by an initial pattern of exploratory activity around the intruder, which was followed by rearing and tail rattle, accompanied in a few seconds by wrestling and/or a violent biting attack. The number of these attacks and latency to the first attack during the 10 min observation period was recorded. The resident intruder test was performed according to Belardo et al. [13].

### Open field

Open Field At 30 and 60 days after mTBI or sham surgery, mice were tested for motor activity and anxiety-like behavior in the open field test. Test Motor activity was also evaluated by open field test in sham and mTBI mice. Briefly, each mouse was individually monitored for 5 min in an open arena (50 cm (length) × 50 cm (width) × 50 cm (height) divided into 16 square grids). Parameters evaluated included: (1) number of transitions; (2) number of rearings; and (3) time spent in the periphery or center (s).

### Novel object recognition (NOR)

To assess learning and long-term memory the Novel Object Recognition (NOR) task was used at 30 and 60 days after mTBI. Two identical objects were placed into the arena during a 6 min sample phase. One of the objects was exchanged by a new object and memory was assessed by comparing the time spent exploring the novel object as compared with the time spent exploring the familiar object during a 5 min test phase. One week before the NOR experiments, the animals experienced handling by the experimenter and habituation to the arena for 5 consecutive days before the habituation, respectively. For habituation, mice were placed into the empty arena (40 × 30 × 30 cm width × length × height, PVC) for 60 min. For NOR experiments custom-built plastic pieces (Polyoxymethylen, POM), were used with different shapes (bell: 5 cm diameter, 6 cm height; diamond: 7 × 7 × 7 cm; cube 5 × 5 × 5 cm) and same colour (black) or different colour and size (glass: 8.3 cm diameter, 8.5 cm height; cup: 6 cm diameter, 6 cm height). The objects were cleaned thoroughly with 70% ethanol followed by distilled water between trials to remove olfactory cues. During the sample phase on the first day of the NOR test, the mice were allowed to explore the two identical black objects (two bells) for 6 min. For the short-delay test phase (1.5 h) one of the sample objects was replaced by a new one (bell by diamond) and exploration was measured for 5 min. For the long-delay test phase (24 h) the new object was again exchanged by another new object. The location of the novel object at 24 h was always different from that at 1.5 h, either first left then right, or vice versa. Consequently, the location of the familiar object also switched between the two test phases. Objects with the same colour but different shapes were considered to be similar to acquisition objects. Active exploration was defined as direct sniffing or whisking towards the objects or direct nose contact. Climbing over the objects was not counted as exploration. The relative exploration was quantified by normalizing the difference between the exploration time of the novel (Tn) and familiar object (Tf) by the total time of exploration (Ttot) to calculate the NOR discrimination index: NOR index = (Tn–Tf)/Ttot. With identical acquisition objects the NOR index was always less than 0.2 indicating that there was no side preference in the mice used for the study.

### Y maze

To assess spatial memory the Y maze test was used at 30 and 60 days post injury [14], with a forced alternation protocol adapted by Wolf et al. [15]. The apparatus consisted of three enclosed arms (30 × 5 × 15 cm; length x width x height) converging on an equilateral triangular center (5 × 5 × 5 cm). At the beginning of the experimental session, each mouse was placed in the center platform and the number of spontaneous alternations (defined as a number of successive triplet entries into each of the three arms without any repeated entries) was monitored in a 5 min test session. The percentage of alternation was calculated as the percentage of the ratio of the number of alternations/ (total number of arm entries − 2).

### Aβ and pro-inflammatory markers measurement

The levels of Aβ (1–40 and 1–42) and pro-inflammatory cytokines (IFN-γ, IL-6, IL-22, IL-17A, TNF-α and IL-1β) were measured in the plasma samples, in the different experimental conditions, by SinglePlex ELISA kit (Elabscience and Diaclone) with a GENios-Pro Reader (Tecan) following the manufacturer's instructions.

### RNA extraction and quantitative PCR (qPCR)

Total RNA was isolated from the hippocampus and cortex by use of the TRIzol Reagent (Cat# 15596026; ThermoFisher, Italy), reacted with DNase-I (Cat# 180680151U/µl; ThermoFisher, Italy) for 15 min at room temperature, followed by spectrophotometric quantification. The final preparation of RNA was considered DNA- and protein-free if the ratio between readings at 260/280 nm was ≥ 1.7. Isolated mRNA was reverse-transcribed by the use of iScript™ Reverse Transcription Supermix (Cat# 1708840; Biorad, Italy). Quantitative PCR (qPCR) was carried out in a real-time PCR system CFX384 (Bio-Rad) using the SYBR Green PCR Kit (Cat# 1725274, Biorad; Italy) Each sample was amplified simultaneously in quadruplicate in a one-assay run with a nontemplate control blank for each primer pair to control for contamination or primer-dimer formation, and the cycle threshold (Ct) value for each experimental group was determined. The housekeeping gene ribosomal protein S16 was used to normalize the Ct values, using the 2^−ΔCt^ formula. Differences in mRNA content between groups were expressed as 2^−ΔΔCt^, as previously described [16].

### Lipid extraction and eCBome analysis

Brain tissues (hippocampus and cortex) were frozen in liquid nitrogen immediately after dissection, which took place within 5 min from sacrifice. Frozen tissues were then homogenized and extracted with chloroform/methanol/Tris–HCl 50 mM pH 7.5 (2:1:1, v/v) containing internal deuterated standards for AEA, 2-AG, PEA, OEA, DHEA, EPEA, OlGly and *N*-acylserotonins quantification by isotope dilution (5 pmol for [^2^H]_8_AEA; 50 pmol for [^2^H]_5_2-AG, [^2^H]_4_PEA, and [^2^H]_2_OEA; 10 pmol for [^2^H]_4_DHEA, [^2^H]_4_EPEA, [^2^H]_8_OlGly, [^2^H]_17_OA5HT). Then the lipid extract was purified using open bed chromatography with silica gel. Fractions enriched in eCBs and *N*-acylethanolamines (9:1, CHCl_3_/CH_3_OH, v/v) were analyzed by liquid chromatography-atmospheric pressure chemical ionization-single quadrupole mass spectrometry (LC–APCI–MS, Shimadzu), as previously described [12, 17], whereas *N*-acylglycines and *N*-acylserotonins were analyzed by liquid chromatography coupled to an ion trap-time of flight mass spectrometry with electrospray ion source (LC–ESI–MS–IT–TOF, Shimadzu). Endogenous levels of eCBome mediators were calculated on the basis of their area ratio with the internal deuterated standard signal areas, all *N*- acylserotonins were calculated on the basis of their area ratio with the OA5HT deuterated standard signal areas. In the case of unsaturated monoacylglycerols, the data are presented as 2-monoacylglycerols (2-MAGs) but represent the combined signals from the 2- and 1(3)-isomers since the latter are most likely generated from the former via acyl migration from the *sn*-2 to the *sn*-1 or *sn*-3 position.

### MALDI-MSI analysis

The mouse brain samples were mounted in a cryostat microtome (Leica CM3050S, Leica Microsystems, Wetzlar, Germany) attached by using drops of water and sectioned at thicknesses of 12 μm at -20 °C upon one-hour conditioning. Tissue sections were thaw-mounted onto pre-cooled indium tin oxide (ITO) coated glass slides (Bruker Daltonics, Bremen, Germany) and stored at -80 °C until further use. MALDI-MSI for neurotransmitters were performed on coronal rat sections at level -2.06 mm (distance from bregma) which was selected as the most representative for investigating the brain areas, Cortex (CE) and Hippocampus (HP).

Sections were desiccated at room temperature 1 h prior to acquiring the optical images on a reflecta® MF5000 scanner (reflecta®, Germany) with HistoView Tissue Scanner II software v1.00.90.

Neurotransmitters of serotonergic metabolic pathway were visualized by on-tissue chemical derivatization with reactive matrix 4-(anthracen-9-yl)-2-fluoro-1-methylpyridin-1-ium iodide salt (FMP-10) [18], with slight modifications. Briefly, FMP-10 was dissolved in 70% ACN (4.4 mM, 5.5 mL), an automated pneumatic sprayer (TM-Sprayer, HTX Technologies) was used to spray the solution over the tissue sections in twenty-five passes at 90 °C, with a nitrogen gas pressure of 10 psi, flow rate of 80 μL/min, nozzle velocity of 1100 mm/min and 2 mm track spacing.

All MALDI-MSI experiments were performed on a solariX 7 T ESI/MALDI-Fourier-transform ion cyclotron resonance mass spectrometer (Bruker Daltonics) equipped with a Smartbeam II 2 kHz laser. The analyses were performed in positive ion mode, at a lateral resolution of 60 μm, with 100 laser shots per position. The laser power was optimized at the start of each run and then held constant during the experiment. The data were acquired in the mass range of m/z 200–1500 using 1 million data points. The ion transfer optics parameters were set as follows: funnel RF amplitude 120.0 Vpp, RF amplitude TOF 350.0 Vpp, TOF 0.6 ms, and RF frequency transfer optic 4 MHz.

The MS method was externally calibrated using red phosphorus dissolved in 50% ACN and spotted beside the tissue section and internal calibration was applied using theFMP-10 cluster ion (m/z 555.2231) as lock mass. Tissue sections were analysed in a random order to prevent any possible bias due to matrix degradation or variation in mass spectrometer sensitivity. The investigated neurotransmitters and metabolites were identified by exact mass matching with a mass tolerance ± 1.5 ppm (Table S2). In addition, their anatomical distribution and order of derivatization, corresponding to the number of attached FMP-10 molecules, were used for confirmation, as previously described [18, 19].

All MSI data were visualized using flexImaging (Bruker Daltonics, version 6.0) and the FT-ICR spectra were normalized against the root-mean-square (RMS) of all data points. Only the ion intensity of OA5HT was normalized to the corresponding deuterated standard, consisting of 1 μg/mL OA5HT-d17 prepared in 50% methanol and homogeneously sprayed over the tissues using an automated sprayer under the following conditions: nozzle temperature was set at 90 °C, with a nitrogen gas pressure of 6 psi, solvent flow rate of 70 μL/min, nozzle velocity of 1100 mm/min, six passes with horizontal/vertical rotation, and 2 mm track spacing.

The reduced spectra were imported into the SCiLS Lab software package (v.2021a, Bruker Daltonics) and the mean ion intensity for each compound in the average mass spectra of the region of interest (either the whole tissue section or a specific brain region) was exported and used for data exploration and statistical analysis.

### Immunoprecipitation

The cortex was lysed in a buffer solution (Tris·HCl 50 mM, NaCl 150 mM, 1% Triton X-100, EDTA 2 mM, EGTA 5 mM, and protease inhibitors mixture) and then the lysate collected and maintained under agitation for 30 min at 4 °C before centrifugation for 15 min at 15,000 × g. Supernatants were recovered and quantified by a Bio-Rad DC Protein Assay Kit. Subsequently, 0.8–1 mg of each lysate sample was used for the immunoprecipitation (IP) procedure using a Dynabeads Protein G Kit (catalog no. 10007D; Life Technology) following the manufacturer’s instructions. Rabbit anti-CB_1_ (IgG) (catalog no. Y080037) was used to immunoprecipitate 5-HT_2A_ protein. IP samples were recovered after heating at 70 °C for 10 min in β-mercaptoethanol sample buffer (2 ×) and loaded on precast polyacrylamide gels (4–12% gradient; Bolt Bis–Tris Plus gels; Life Technologies) and then transferred to a PVDF membrane. Rabbit anti-5-HT_2A_ antibody (1:1000) was incubated overnight at 4 °C. All of the proteins not pulled down by the anti-CB_1_ antibody/beads complex (flow-through) were loaded (at a concentration of 50 μg) on precast polyacrylamide gels and blotted using anti-5-HT_2A_ and anti–α-tubulin (1:500; Sigma-Aldrich) antibodies as a control for the IP procedure.

### Statistical analysis

Data analysis was performed by Prism Software 9.0. Data are represented as boxplots with individual points. Two-way ANOVA or Two-way ANOVA for repeated measures were performed to examine the influence of two different categorical independent variables on one continuous dependent variable. Different multicomparison corrections were applied for different types of analysis. For the behaviour, Dunnett’s or Sidak’s post hoc tests were performed for inter- and intra-group analysis, respectively. Tukey’s multicomparisons correction was performed for ex vivo analysis. Finally, correlation analyses were carried out by Spearman correlation coefficient. For all the analysis, a *P* value (*P*) < 0.05 was considered significant. Detailed statistical analysis containing normality distribution tests and F scores are reported as Additional file 1 (Table S1).

## Results

### mTBI in WT mice causes alterations in cognitive performance similar to those observed in pre-symptomatic APP-SWE genotype

**Fig. 2:**
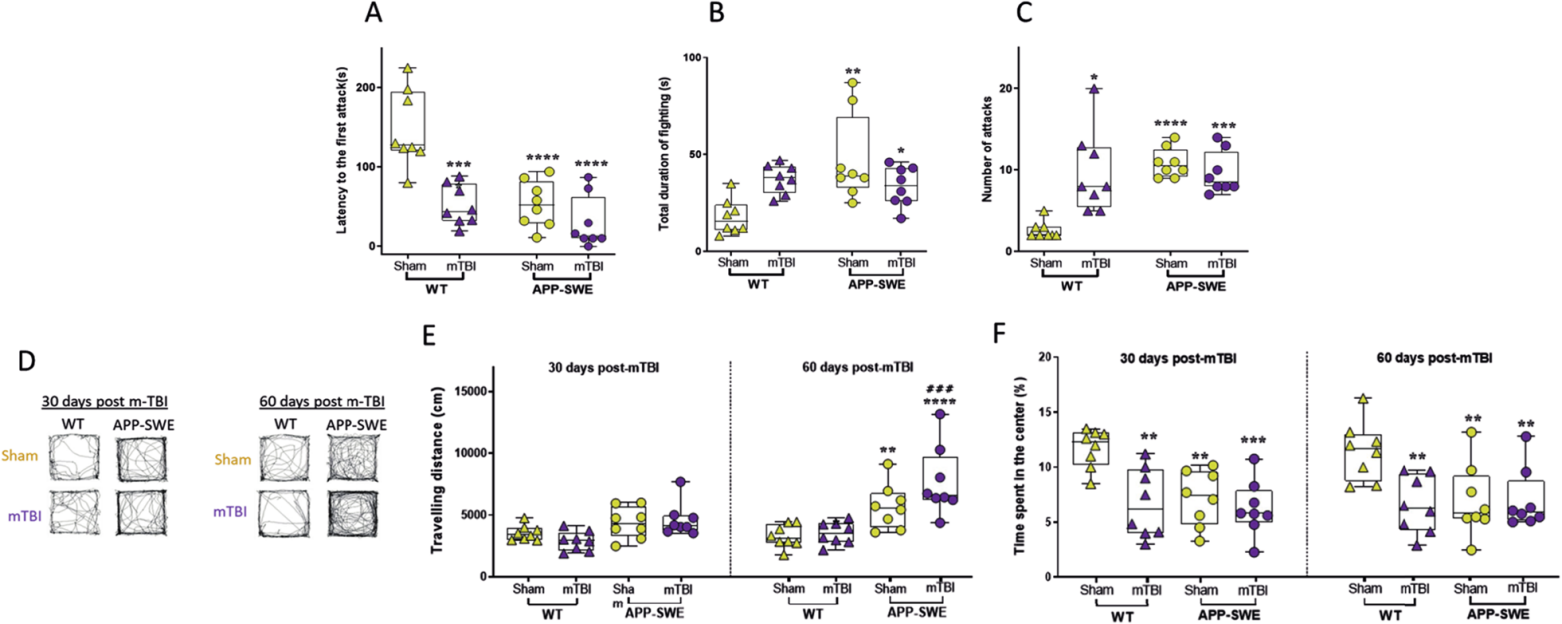
Effects of mTBI on aggressiveness and locomotion in WT or APP-SWE mice. **A**–**C** show the latency to the first attack (s), duration of fighting (s) and the number of attacks in the resident intruder test. **D** shows representative tracks of the total travelled distance in each group of mice in the open field. **E**, **F** show the total travelling distance (cm) and the time spent in the centre (s) in the open field test. Data are shown as mean ± SEM of 8 mice per group, 30 and 60 days post mTBI induction. Two-way ANOVA or Two-way ANOVA for repeated measures were performed for the resident intruder test and OFT 30–60 days post-mTBI, respectively. Dunnett’s or Sidak’s post hoc tests were applied for multiple comparisons inter- or intra- group, respectively (*indicates significant differences compared to WT sham; # indicates significant differences compared to the same group at 30 days post mTBI)

15 days after trauma induction, mice exhibited aggressive behaviour, as suggested by the resident intruder test. A reduced latency to attack, as well as, an increased number of attacks and duration of total fighting were observed in WT mTBI mice, as compared with Sham mice (latency to the first attack: WT mTBI 51.38 s ± 8.91 vs WT sham 148.3 ± 17.20). Two-way ANOVA showed significant differences for interaction (F_1, 28_ = 8.725, *P* = 0.0063), genotype (F_1, 28_ = 22.34, *P* < 0.0001) and type of injury (F_1, 28_ = 23.70, *P* < 0.0001); number of attacks WT mTBI 9.75 ± 1.79 vs WT sham 2.63 ± 0.38, Two-way ANOVA showed significant differences for interaction (F_1, 28_ = 6.7527, *P* = 0.0163) and for genotype (F_1, 28_ = 21.35, *P* < 0.0001) but not for type of injury (F_1, 28_ = 2.154, *P* = 0.1533); duration of total fighting: WT mTBI 37.38 s ± 2.61 vs WT sham 17.90 ± 3.18, Two-way ANOVA showed significant differences for interaction (F_1, 28_ = 8.594, *P* = 0.0066), but not for genotype (F_1, 28_ = 0.3779, *P* = 0.5437) and type of injury (F_1, 28_ = 3.940, *P* = 0.0570)] (Fig. 2A–C).

The open field test was used to evaluate the motor and exploratory activity, as well as the possible anxiety-like behaviour. No significant change in the travelled distance was observed between sham and mTBI groups (Fig. 2D). Regardless of the trauma, APP-SWE mice showed a substantial increase in the total travelling distance, indicating an overall hyperlocomotion, as compared with control animals (APP-SWE Sham 4365.48 cm ± 445.43 vs WT Sham 3555.94 cm ± 217.85, APP-SWE Sham 5690.03 cm ± 637.67 vs WT Sham 3288.94 cm ± 323.85 for 30 and 60 days post trauma, respectively, and APP-SWE mTBI 60 days 7711.08 cm ± 984.20 vs APP-SWE mTBI 30 days 4621.62 cm ± 473.91). Two-way ANOVA for repeated measures showed significant differences for interaction (F_3, 28_ = 4.178, *P* = 0.0145), for time (F_1, 28_ = 12.03, *P* = 0.00017) and type of injury (F_3, 28_ = 13,41, *P* < 0.0001) but not for Subjects (matching) (F_28, 28_ = 1.213, *P* = 0.3068)] (Fig. 2E). The time spent in the centre was significantly reduced (at 30 and 60 days) following the trauma in WT mice, suggesting an anxiety-like behaviour (WT mTBI 6.71% ± 1.10 vs WT Sham 11.72% ± 0.62 and WT mTBI 6.55% ± 0.91 vs WT Sham 11.46% ± 0.94 for 30 and 60 days post trauma, respectively). Two-way ANOVA for repeated measures showed a significant difference only for type of injury (F_3, 28_ = 11.40, *P* < 0.0001) but not for interaction (F_3, 28_ = 0.1559, *P* = 0.9250), time (F_1, 28_ = 0.002758, *P* = 0.9585) and subjects (matching) (F_28, 28_ = 1.340, *P* = 0.2220). However, this effect was not observed in APP-SWE, which exhibited a higher baseline level of anxiety, as compared with WT animals (time spent in the center 30 days post injury: APP-SWE sham 7.15% ± 0.89 vs WT Sham 11.72% ± 0.62, APP-SWE mTBI 6.25% ± 0.88 vs WT Sham 11.72% ± 0.62; time spent in the center 60 days post injury: APP-SWE sham 6.93% ± 1.16 vs WT Sham 11.72% ± 0.62, APP-SWE mTBI 7.02% ± 0.97 vs WT Sham 11.72% ± 0.62), (Fig. 2F).

**Fig. 3:**
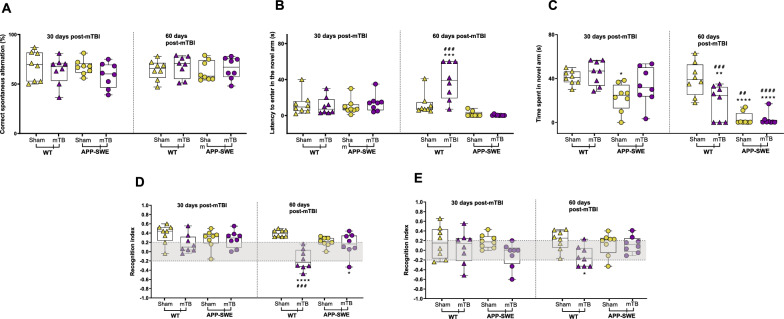
Effects of mTBI on cognitive performance in WT or APP-SWE mice. **A** shows the percentage of the alternation in the Y-maze test. **B**, **C** show the latency to enter the novel arm (s) and time spent in the novel arm (s) in the forced protocol of Y-maze. **D**, **E** shows NOR index in the novel object recognition test at 1.5-h and 24-h delay, respectively. Two-way ANOVA or Two-way ANOVA for repeated measures were performed for the resident intruder test and OFT 30–60 days post-mTBI, respectively. Dunnett’s or Sidak’s post hoc tests were applied for multiple comparisons inter- or intra- group, respectively (* indicates significant differences compared to WT sham; # indicates significant differences compared to the same group at 30 days post mTBI)

To investigate possible cognitive deficits due to the trauma, we performed specific tasks measuring spatial and discriminative memory functioning. We found no significant impairment of spatial working memory, measured as spontaneous alternation behaviour in the Y maze (Y- maze 30 and 60 days: WT Sham 68.13% ± 5.21 and 64.33% ± 3.53 30; APP-SWE Sham 63.41% ± 5.0 and 67.78% ± 6.90 30; APP-SWE sham 67.04% ± 2.68 and 63.13% ± 3.60, APP-SWE mTBI 58.70 5 ± 4.47 and 66.0% ± 3.89 30). Two-way ANOVA for repeated measures showed no difference for interaction (F_3, 28_ = 1,009, *P* = 0.4036), time (F_1, 28_ = 0,1202, *P* = 0.7315) type of injury (F_3, 28_ = 0,3342, *P* = 0.8007) and subjects (matching) (F_28, 28_ = 1,075, *P* = 0.4251)] (Fig. 3A). However, using the forced alternation protocol, we found an increase in the latency of entry, as well a decrease of the time spent in the novel arm in WT mTBI animals, 60 days post injury [latency to enter in the novel arm: WT Sham 12.63 s ± 4.24 vs WT mTBI 38.50 s ± 7.34; Two-way ANOVA for repeated measures showed a difference for interaction (F_3, 28_ = 12.66, *P* < 0.0001), type of injury (F_3, 28_ = 8.287, *P* = 0.0004) but not for time (F_1, 28_ = 0.2968, *P* = 0.5902) and subjects (matching) (F_28, 28_ = 1.319, *P* = 0.2345); time spent in the novel arm: WT Sham 63.41% ± 5.0 vs APP-SWE Sham 67.78% ± 6.90; Two-way ANOVA for repeated measures showed difference for interaction (F_3, 28_ = 4,990, *P* = 0.0067), time (F_1, 28_ = 45.11, *P* < 0.0001) and type of injury (F_3, 28_ = 16.14, *P* < 0.0001) but not for subjects (matching) (F_28, 28_ = 1.150, *P* = 0.3569)] (Fig. 3B). In APP mice after both sham and mTBI, we detected a strong decrease in the time spent in the novel arm, while the latency was not affected (Fig. 3C).

In the Novel object recognition test all groups of mice were tested on the capability of mnemonic discrimination of dissimilar objects (recognition memory). In the acquisition phase, mice were exposed to two identical objects with the same colour, size, and shape. The total exploration time during the acquisition phase was not different between groups (not shown). All groups of mice had an exploration time of less than 0.2 indicating that there was no side preference. However, 60 days post injury, WT mice showed a reduced recognition index, indicating an impaired discriminative memory performance (1.5 or 24 h delay), (NOR 1.5 h: WT Sham 0.40 ± 0.03 vs APP-SWE Sham -0.17 ± 0.08). Two-way ANOVA for repeated measures showed difference for interaction (F_3, 28_ = 3.827, *P* = 0.0205), time (F_1, 28_ = 9.928, *P* = 0.0039) and type of injury (F_3, 28_ = 9.218, *P* = 0.0002) but not for subjects (matching) (F_28, 28_ = 3.597, *P* = 0.0257).  The NOR 24 h showed significant differences between WT Sham and APP-SWE Sham (WT Sham 0.21 ± 0.07 vs APP-SWE Sham: − 0.13 ± 0.07). Two-way ANOVA for repeated measures showed a difference for the type of injury (F_3, 28_ = 9.218, *P* = 0.0002) but not for interaction (F_3, 28_ = 1.779, *P* = 0.1740), time (F_1, 28_ = 0.01077, *P* = 0.9181) and subjects (matching) (F_28, 28_ = 0.7785, *P* = 0.7440)]. On the other hand, we found that brain trauma did not induce significant changes in APP-SWE mice (Fig. 3D, E).

### mTBI induces Aβ_1−42_ and BACE1 in the cortex of APP-SWE mice

**Fig. 4:**
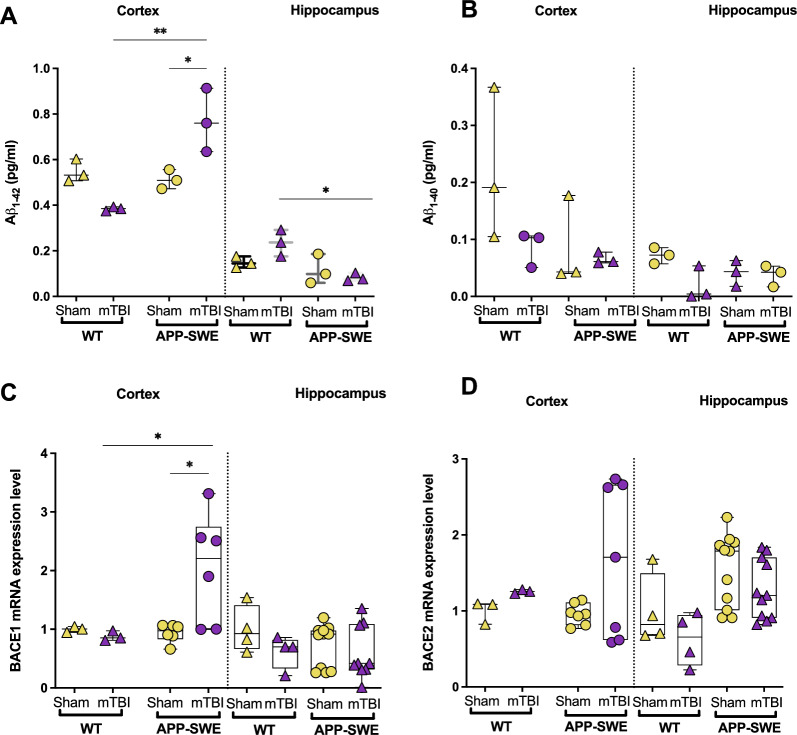
Effects of mTBI on beta-amyloid and beta-site amyloid precursor protein cleaving enzymes (BACE 1 and 2) transcript levels in brain areas of APP-SWE mice and their WT controls. **A** Aβ_1−42_ and **B** Aβ_1−40_ levels in the cortex and hippocampus of WT and APP-SWE mice (sham and mTBI). **C** BACE1 and **D** BACE2 gene expression levels in cortex and hippocampus of WT and APP-SWE mice (sham and mTBI). Statistical analysis was performed by Two-way analysis of variance (ANOVA) followed by Tukey's multiple comparison test (*P ≤ 0.05; **P ≤ 0.01). Data are represented as boxplots with individual points

Although we did not detect substantial differences between sham and mTBI groups in the APP-SWE genotype, we evaluated possible quantitative changes due to the trauma, in early-stage biomarkers for AD, such as Aβ_1–40_, Aβ_1−42_ and the beta-site amyloid precursor protein cleaving enzyme 1 (BACE1). Our data show an increase of Aβ_1−42_ in the cortex of APP-SWE mice subjected to mTBI in comparison to sham APP-SWE (*P* < 0.05) or WT mTBI (*P* < 0.01, Fig. 4A). Two-way ANOVA showed significant differences for interaction (F_1, 8_ = 22.31, *P* = 0.0015), genotype (F_1, 8_ = 15.49, *P* = 0.0043) but not for the type of injury (F_1, 8_ = 1.118, *P* = 0.3213). On the other hand, in the hippocampus, Aβ_1−42_ levels were significantly decreased in comparison to WT mTBI (*P* < 0.05, Fig. 4A). Two-way ANOVA showed significant differences for genotype (F_1, 8_ = 12.32, *P* = 0.0080) but not for interaction (F_1, 8_ = 4.853, *P* = 0.0587) and type of injury (F_1, 8_ = 1.032, *P* = 0.3395). No significant changes were induced by the trauma in WT animals. As shown in Fig. 4B, Aβ_1−40_ did not undergo any significant changes in either brain area analysed.

Moreover, we analysed gene expression levels of beta-secretase 1, also known as beta-site amyloid precursor protein cleaving enzyme 1 (BACE1), the enzyme responsible for amyloid precursor protein (APP) cleavage and subsequent formation of Aβ peptides, including Aβ_1-42_, which aggregates into bioactive conformational species and likely initiates toxicity in AD. Furthermore, there exists ahomologous BACE2, which shares 59% of its amino acid sequence and is composed of an identical structural domain as BACE1 [20]. However, while the role of BACE1 in the pathogenesis of AD has received significant attention, that of BACE2 has not. Therefore, we investigated the gene expression levels of both BACE1 and BACE2 in the cortex and hippocampus of WT and APP-SWE mice subjected to mTBI. As shown in Fig. 4C, BACE1 significantly increased in the cortex of mTBI APP-SWE in comparison to their sham controls (*P* < 0.05), while it did not change in the hippocampus.

Two-way ANOVAs showed significant differences in the cortex for interaction (F_1, 14_ = 4.783, *P* = 0.0462) but not for genotype (F_1, 14_ = 3.866, *P* = 0.0694) and type of injury (F_1, 14_ = 3.092, *P* = 0.1005). In the hippocampus, two-way ANOVA showed no significant differences for interaction (F_1, 24_ = 0.5775, *P* = 0.4547), for genotype (F_1, 24_ = 0.7459, *P* = 0.3963) and type of injury (F_1, 24_ = 2.445, *P* = 0.1310). On the other hand, in neither analysed area, BACE2 did undergo any significant changes (Fig. 4D).

### mTBI induces elevated plasmatic levels of pro-inflammatory cytokines

A neuroinflammatory response is triggered by mTBIs that lead to perturbations in the levels of inflammatory cytokines [21, 22]. However, most studies focused on the acute phase and/or on the most severe forms of brain trauma, whereas chronic neuroinflammation might be the most notable pathological feature shared by mTBI and AD and it is worth investigating the immune response at later time points. Therefore, we analysed peripheral levels of interleukin 1 beta (IL-1β), tumor necrosis factor alpha (TNFα), interleukin 22 (IL-22), interleukin 6 (IL-6), interleukin 17A (IL-17A) and interferon gamma (IFN-γ) at the end of the behavioural studies, 60 days post-mTBI.

**Fig. 5:**
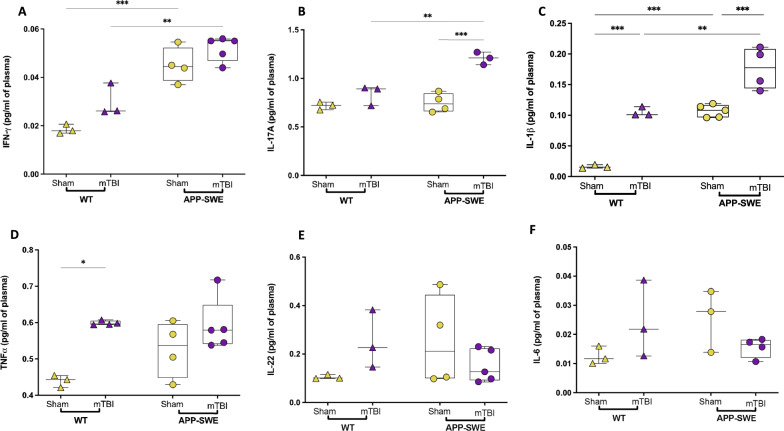
Plasmatic levels of pro-inflammatory cytokines in WT and APP-SWE mice (sham and mTBI). **A** interferon gamma (IFN-γ); **B** interleukin 17A (IL-17A); **C** interleukin 1 beta (IL-1β); **D** tumor necrosis factor alpha (TNFα); **E** interleukin 22 (IL-22) and **F** interleukin 6 (IL-6) expressed as pg/ml of plasma. Statistical analysis was performed by Two-way analysis of variance (ANOVA) followed by Tukey's multiple comparison test (*P ≤ 0.05; **P ≤ 0.01; ***P ≤ 0.001). Data are represented as boxplots with individual points

In particular, IFN-γ increased significantly in APP-SWE sham in comparison to WT sham (Fig. 5A, *P* < 0.01) and APP-SWE mTBI as compared to WT mTBI mice (*P* < 0.01).

Two-way ANOVAs showed significant differences for genotype (F_1, 11_ = 64.86, *P* < 0.0001) and type of injury (F_1, 11_ = 9.091, *P* = 0.0118) but not for interaction (F_1, 11_ = 0.5635, *P* = 0.4686). IL-17A, shown in Fig. 5B, increased significantly in mTBI APP-SWE compared to WT mTBI (*P* < 0.01) and APP-SWE sham (*P* < 0.001). Two-way ANOVA showed significant differences for interaction (F_1, 9_ = 13.59, *P* = 0.0050), for genotype (F_1, 9_ = 18.96, *P* = 0.0018) and type of injury (F_1, 9_ = 39.42, *P* = 0.0001). IL-1β (Fig. 5C) increased significantly (*P* < 0.01) in WT mTBI vs. WT-sham and APP-SWE mTBI compared to APP-SWE sham and WT mTBI (*P* < 0.001 and 0.01, respectively). Two-way ANOVA showed significant differences for genotype (F_1, 11_ = 62.33, *P* < 0.0001) and type of injury (F_1, 11_ = 62.33, *P* < 0.0001) but not for interaction (F_1, 11_ = 0.9424, *P* = 0.3525). Instead, TNF-α (Fig. 5D) increased significantly only in WT mTBI compared to WT sham (*P* < 0.05), and no significant alteration was observed in APP-SWE mice. Two-way ANOVA showed significant differences in the type of injury (F_1, 12_ = 14.78, *P* = 0.0023) but not for interaction (F_1, 12_ = 2.589, *P* = 0.1336) and genotype (F_1, 12_ = 1.922, *P* = 0.1909). On the other hand, IL-22 and IL-6 (Fig. 5E, F, respectively) did not undergo any change.

### Lipid and gene expression profiles of the eCBome in WT and APP-SWE mice following mTBI

Here we have applied an integrative methodology based on the combination of LC-APCI-MS, LC-HRMS and MALDI-MS imaging to elucidate more comprehensively the metabolomics and lipidomics alterations induced, with spatiotemporal features, in the brain. Moreover, to try to explain the observed changes in the brain levels of eCBs and eCB-like molecules, and understand their potential consequences, we also investigated the gene expression levels of their biosynthetic and degradative enzymes, as well as of their molecular targets.

#### Targeted lipidomics

**Fig. 6:**
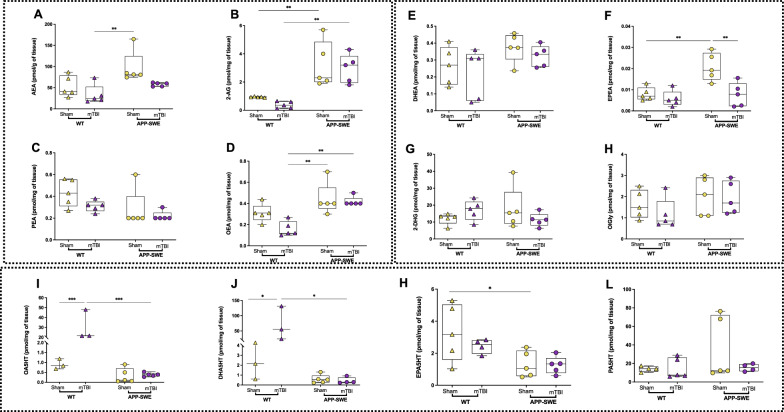
Effects of mTBI on endocannabinoids and *N*-acylethanolamine, monoacylglycerol and *N*-acylserotonin levels in the cortex of APP-SWE mice and their WT controls. **A** Levels of anandamide (AEA) expressed as pmol/g of tissue weight. **B** Levels of 2-arachidonoyl glycerol (2-AG) expressed as pmol/mg of tissue weight. **C** Levels of *N*-palmitoylethanolamine (PEA) expressed as pmol/mg of tissue weight. **D** Levels of *N*-oleoylethanolamine (OEA) expressed as pmol/mg of tissue weight. **E** Levels of *N*-docosahexaenoylethanolamine (DHEA) expressed as pmol/mg of tissue weight. **F** Levels of *N*-eicosapentaenoyl ethanolamine (EPEA) expressed as pmol/mg of tissue weight. **G** 2-docosahexaenoylglycerol (2-DHG) expressed as pmol/mg of tissue weight. **H** *N*-oleoylglycine (OlGly) expressed as pmol/mg of tissue weight. **I** *N*-oleoylserotonin (OA5HT) expressed as pmol/mg of tissue weight. **J** *N*-docosahexaenoylserotonin (DHA5HT) expressed as pmol/mg of tissue weight. **K** *N*-eicosapentaenoylserotonin (EPA5HT) is expressed as pmol/mg of tissue weight. **L** *N-*palmitoylserotonin (PA5HT) is expressed as pmol/mg of tissue weight. Statistical analysis was performed by Two-way analysis of variance (ANOVA) followed by Tukey's multiple comparison test (*P ≤ 0.05; **P ≤ 0.01). Data are represented as boxplots with individual points

**Fig. 7:**
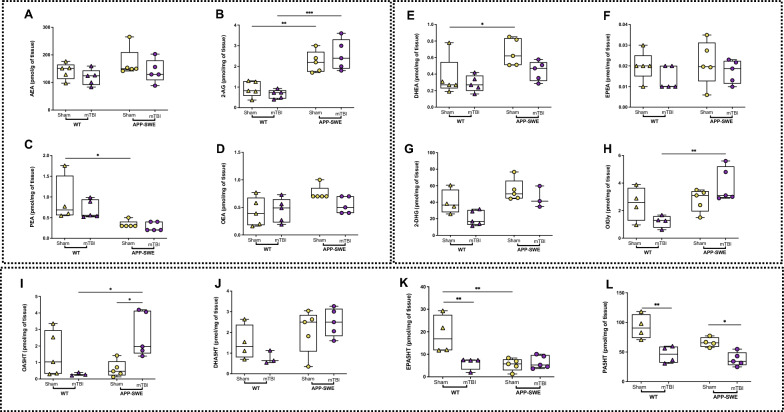
Effects of mTBI on endocannabinoids and *N*-acylethanolamine, monoacylglycerol and *N*-acylserotonin levels in the hippocampus of APP-SWE mice and their WT controls. **A** Levels of anandamide (AEA) expressed as pmol/g of tissue weight. **B** Levels of 2-arachidonoyl glycerol (2-AG) expressed as pmol/mg of tissue weight. **C** Levels of *N*-palmitoylethanolamine (PEA) expressed as pmol/mg of tissue weight. **D** Levels of *N*-oleoylethanolamine (OEA) expressed as pmol/mg of tissue weight. **E** Levels of *N*-docosahexaenoyl ethanolamine (DHEA) expressed as pmol/mg of tissue weight. **F** Levels of *N*-eicosapentaenoyl ethanolamine (EPEA) expressed as pmol/mg of tissue weight. **G** 2-docosahexaenoylglycerol (2-DHG) expressed as pmol/mg of tissue weight. (H) *N*-oleoylglycine (OlGly) is expressed as pmol/mg of tissue weight. **I** *N*-oleoylserotonin (OA5HT) expressed as pmol/mg of tissue weight. **J** *N*-docosahexaenoylserotonin (DHA5HT) expressed as pmol/mg of tissue weight. **K** *N*-eicosapentaenoylserotonin (EPA5HT) expressed as pmol/mg of tissue weight. **L** *N-*palmitoylserotonin (PA5HT) expressed as pmol/mg of tissue weight. Statistical analysis was performed by Two-way analysis of variance (ANOVA) followed by Tukey's multiple comparison test (*P ≤ 0.05; **P ≤ 0.01). Data are represented as boxplots with individual points

In particular, with targeted lipidomics analysis we have analyzed several eCBs and eCB-like mediators, that could be divided into three main families:

1. Classical eCBs and eCB-like mediators, including AEA, 2-AG, PEA and OEA (Figs. 6, 7A–D);
2. Long-chain *N*-acyl amides and monoacylglycerols, including *N*-docosahexaenoylethanolamine (DHEA), *N*-eicosapentaenoylethanolamine (EPEA), 2-docosahexaenoylglycerol (2-DHG) and *N*-oleoylglycine (OlGly) (Figs. 6, 7E–H);
3. *N*-acyl-neurotransmitters, including *N*-oleoylserotonin (OA5HT), *N*-docosahexaenoylserotonin (DHA5HT), *N*-eicosapentaenoylserotonin (EPA5HT) and *N*-palmitoylserotonin (PA5HT) (Figs. 6, 7I–L).

In the cortex (Fig. 6), 2-AG levels significantly increased in APP-SWE in comparison to WT mice (*P* < 0.01 for both WT sham vs APP-SWE sham and WT mTBI vs APP-SWE mTBI Fig. 6B), but no difference between sham and mTBI in APP-SWE mice was observed. Two-way ANOVA showed a significant difference for genotype (F_1, 16_ = 31.31, *P* < 0.0001) but not for interaction (F_1, 16_ = 0.1334, *P* = 0.7197) and for type of injury (F_1, 16_ = 0.8468, *P* = 0.3711). OEA increased in APP-SWE injured mice in comparison to WT mTBI, but not if compared to sham APP-SWE (*P* < 0.01 Fig. 6D). Two-way ANOVA showed a significant difference for genotype (F_1, 16_ = 20.39, *P* = 0.0004) but not for interaction (F_1, 16_ = 2.265, *P* = 0.1518) and type of injury (F_1, 16_ = 3.874, *P* = 0.0666). Regarding long chain *N*-acylethanolamines, only EPEA underwent to a significant change, in particular its levels increased in APP-SWE sham mice compared to WT sham and decreased in APP-SWE mTBI compared to sham mice of the same genotype (*P* < 0.01 both, Fig. 6F). Two-way ANOVA showed significant differences for interaction (F_1, 16_ = 5.897, *P* = 0.0273), for genotype (F_1, 16_ = 10.75, *P* = 0.0047) and for type of injury (F_1, 16_ = 11.66, *P* = 0.0036). To better understand a possible correlation between eCBome mediators that underwent significant alterations, and the possible predisposing effect of mTBI and the APP genotype, we carried out a Spearman correlation analysis (Supplementary Figure S1). Very interestingly, in the cortex of WT mice, Aβ_1−42_ strongly correlated with 2-AG levels (r_s_ = 1), in both sham and mTBI groups. On the other hand, in APP-SWE mice, while in the sham group only EPEA positively correlated with Aβ_1−42_ (r_s_ = 1), in the mTBI group 2-AG directly correlated with Aβ_1−42_ but EPEA showed a negative correlation (r_s_ = -1). However, no significance was observed.

Here we reported the identification and quantification in the brain of some species belonging to the *N*-acylserotonin family for the first time (Figs. 6, 7I, J). In particular, in the cortex, mTBI induced a 20-fold increase in WT mice of OA5HT (*P* < 0.01 Fig. 6I), which instead exhibited a significant decrease in APP-SWE mTBI compared to WT mTBI mice (*P* < 0.001 Fig. 6I). No significant change in APP-SWE mice between sham and mTBI groups was observed.

Two-way ANOVA showed significant differences for interaction (F_1, 12_ = 20.95, *P* = 0.0006), for genotype (F_1, 12_ = 22.64, *P* = 0.0005) and type of injury (F_1, 12_ = 21.12, *P* = 0.0006). Also, DHA5HT significantly increased in WT mTBI (*P* < 0.05 WT sham vs WT mTBI) and decreased in APP-SWE mTBI compared to WT mTBI mice (*P* < 0.05 Fig. 6J). No change was induced by trauma in APP-SWE mice compared to their sham. Two-way ANOVA showed significant differences for interaction (F_1, 11_ = 7.475, *P* = 0.0194), for genotype (F_1, 11_ = 8.244, *P* = 0.0152) and for type of injury (F_1, 11_ = 7.404, *P* = 0.0199). Instead, EPA5HT only significantly decreased in APP-SWE sham compared to WT sham mice (*P* < 0.05 Fig. 6K). Two-way ANOVA showed a significant difference for genotype (F_1, 15_ = 10.42, *P* = 0.0056) but not for interaction (F_1, 15_ = 0.6462, *P* = 0.4340) and for type of injury (F_1, 15_ = 0.8421, *P* = 0.3733). Finally, no significant differences among groups were observed for PA5HT levels (Fig. 6L).

Also in the hippocampus, as shown in Fig. 7, 2-AG levels were significantly higher in APP-SWE than in WT mice (*P* < 0.01 WT sham vs APP-SWE sham and *P* < 0.001 WT mTBI vs APP-SWE mTBI Fig. 7B), with no significant change between sham and mTBI in APP-SWE mice. Two-way ANOVA showed a significant difference for genotype (F_1, 16_ = 51.00, *P* < 0.0001) but not for interaction (F_1, 16_ = 1.709, *P* = 0.2096) and for type of injury (F_1, 16_ = 0.04184, *P* = 0.8405). Instead, PEA decreased in APP-SWE sham compared to WT sham mice (*P* < 0.05 Fig. 7C), while DHEA increased (*P* < 0.05 Fig. 7E). Two-way ANOVA showed significant differences for genotype (F_1, 15_ = 14.17, *P* = 0.0019) but not for interaction (F_1, 15_ = 0.3628, *P* = 0.5559) and type of injury (F_1, 15_ = 1.111, *P* = 0.3085). OlGly increased significantly in mTBI APP-SWE mice compared to WT mTBI group (*P* < 0.01 Fig. 7H). Two-way ANOVA showed significant differences for interaction (F_1, 14_ = 6.447, *P* = 0.0236) and for genotype (F_1, 14_ = 9.539, *P* = 0.0080) but not for type of injury (F_1, 14_ = 0.02949, *P* = 0.8661). Interestingly, also OA5HT increased significantly in mTBI APP-SWE mice compared to their sham and the WT mTBI group (*P* < 0.05 both, Fig. 7I). Two-way ANOVA showed significant differences for interaction (F_1, 14_ = 10.21, *P* = 0.0065) but not for genotype (F_1, 14_ = 2.049, *P* = 0.1742) and type of injury (F_1, 14_ = 0.6663, *P* = 0.4280). EPA5HT decreased significantly in WT mTBI compared to their sham (*P* < 0.01, Fig. 7K) and in the APP-SWE sham group (*P* < 0.001 WT sham vs APP-SWE sham), but no effect was observed in injured APP mice. Two-way ANOVA showed significant differences for interaction (F_1, 14_ = 9.879, *P* = 0.0072), for genotype (F_1, 14_ = 8.581, *P* = 0.0110) and for type of injury (F_1, 14_ = 6.944, *P* = 0.0196). Interestingly, PA5HT decreased significantly after mTBI in both WT and APP-SWE mice compared to their respective sham (*P* < 0.001 and 0.05 WT sham vs WT mTBI and APP-SWE sham vs APP-SWE mTBI, respectively, Fig. 7L). Two-way ANOVA showed significant differences for genotype (F_1, 13_ = 6.085, *P* = 0.0283) and for type of injury (F_1, 13_ = 29.56, *P* = 0.0001) but not for interaction (F_1, 13_ = 1.688, *P* = 0.2164). No significant change in other mediators analysed was observed. Moreover, since recently it has been reported that DHA5HT exerts potent immune-modulatory properties by decreasing IL-6 and IL-1β, among others, in lipopolysaccharide (LPS)-stimulated RAW264.7 macrophages [23], we carried out a Spearman correlation analysis (Supplementary Figure S2) to detect possible correlations with inflammatory cytokines in the plasma. Very interestingly, we found that DHA5HT in the hippocampus of WT sham correlated with IFN-γ (r_s_ = 1) and IL-1β (r_s_ = −1), while in WT mTBI mice it correlated with IL-17A and TNF-α (both r = -1). In APP-SWE sham mice, DHA5HT correlated with IL-1β (r = 0.90; *P* = 0.083) and in APP-SWE mTBI mice with IL-17A (r_s_ = 1). On the other hand, in cortex DHA5HT correlated with IL-1β (r_s_ = −0.87) inWT mTBI mice, whilst in APP-SWE sham mice, it correlated with TNF-α (r_s_ = 1, *P* = 0.083) and in the APP-SWE mTBI group with IL-17A (r_s_ = −1). However, no significance was observed.

#### Gene expression analysis

**Fig. 8:**
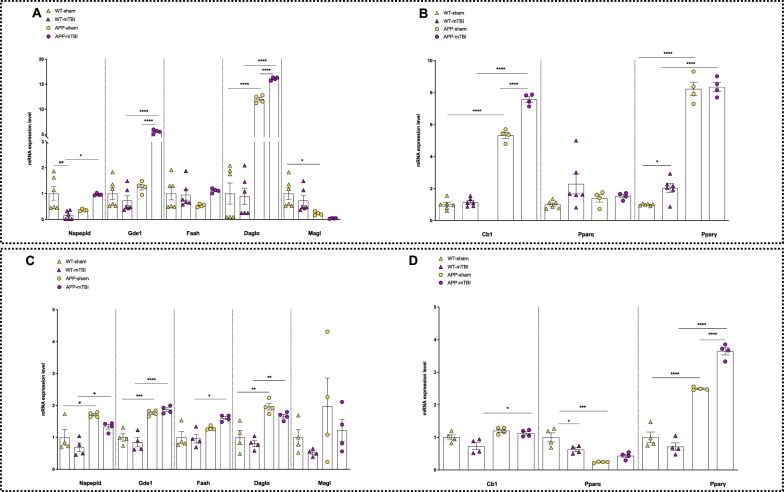
mRNA expression levels of indicated genes analyzed by quantitative PCR analysis in the cortex (**A**, **B**) and hippocampus (**C**, **D**) of wt and APP mice (sham vs mTBI). Differences in mRNA content between groups were expressed as the 2^−ΔΔCt^ formula as reported in the materials and methods section. Statistical analysis was performed by Two-way analysis of variance (ANOVA) followed by Tukey's multiple comparison test (*P ≤ 0.05; **P ≤ 0.01; ***P ≤ 0.001). Data are represented as bar graphs with individual points

Gene expression analysis, measured by qPCR transcriptomics, of the biosynthetic/degradative enzymes and receptors of the eCBome revealed some alterations according to their endogenous levels. In particular, in the cortex, where AEA levels did not change, *Napepld* decreased significantly in WT mTBI in comparison to WT sham and increased in APP-SWE mTBI compared to WT mTBI (*P* < 0.01 and 0.05, respectively, Fig. 8A).

Two-way ANOVA showed a significant difference for interaction (F_1, 16_ = 18.67, *P* = 0.0005) but not for genotype (F_1, 16_ = 0.2591, *P* = 0.6177) and type of injury (F_1, 16_ = 0.5133, *P* = 0.4840). Also *Gde1*, which is another biosynthetic enzyme responsible for AEA and NAE production like *Napepld*, increased significantly in APP-SWE mTBI compared to WT mTBI and APP-SWE sham (*P* < 0.0001 both, Fig. 8A). Two-way ANOVA showed significant differences for interaction (F_1, 16_ = 125.2, *P* < 0.0001), for genotype (F_1, 16_ = 154.2, *P* < 0.0001) and type of injury (F_1, 16_ = 96.94, *P* < 0.0001). On the other hand, *Faah*, a NAE degradative enzyme, did not change. However, the alterations observed may explain only partially the changes in NAE levels observed (i.e. OEA increase in APP-SWE mTBI, and EPEA increase in APP-SWE sham). Regarding 2-AG enzymes, *Dagla* increased significantly in APP-SWE mice (*P* < 0.0001 sham WT vs sham APP-SWE, *P* < 0.0001 for both APP-SWE sham vs APP-SWE mTBI and WTmTBI vs APP-SWE mTBI Fig. 8A), which explains the increased 2-AG biosynthesis in APP-SWE mice, even though trauma did not induce an increased endogenous tone. Two-way ANOVA showed significant differences for interaction (F_1, 16_ = 34.14, *P* < 0.0001), for genotype (F_1, 16_ = 1333, *P* < 0.0001) and type of injury (F_1, 16_ = 30.80, *P* < 0.0001). *Magl* gene expression levels decreased in APP-SWE sham mice in comparison to WT sham ones (*P* < 0.05), again possibly contributing to the observed increase of 2-AG in the former mice. Two-way ANOVA showed a significant difference for genotype (F_1, 16_ = 16.12, *P* = 0.0010) but not for interaction (F_1, 16_ = 0.05423, *P* = 0.8188) and for type of injury (F_1, 16_ = 1.594, *P* = 0.2249).

Three main molecular targets were investigated at the gene expression level: *Cnr1, Ppara* and *Pparg*. In the cortex, *Cnr1* increased significantly in APP-SWE mice (*P* < 0.0001 WT sham vs APP-SWE sham, APP-SWE sham vs APP-SWE mTBI and WT mTBI vs APP-SWE mTBI, Fig. 8B). Two-way ANOVA showed significant differences for interaction (F_1, 16_ = 51.32, *P* < 0.0001), for genotype (F_1, 16_ = 1349, *P* < 0.0001) and type of injury (F_1, 16_ = 66.67, *P* < 0.0001). Also *Pparg* increased significantly in the cortex of APP-SWE in comparison to WT mice (*P* < 0.0001 WT sham vs APP-SWE sham and WT mTBI vs APP-SWE mTBI). Trauma induced an increase in *Pparg* gene expression levels only in WT mice (*P* < 0.05 WT sham vs WT mTBI) with no difference between sham and mTBI groups of APP-SWE animals. On the other hand, *Ppara* did not undergo significant alterations in the cortex. Two-way ANOVA showed significant differences for genotype (F_1, 16_ = 651.2, *P* < 0.0001) and for type of injury (F_1, 16_ = 4.783, *P* = 0.0439) but not for interaction (F_1, 16_ = 2.934, *P* = 0.1061).

In the hippocampus, as shown in Fig. 8C, both *Napepld* and *Gde1* increased in the sham APP-SWE group in comparison to sham WT (*P* < 0.05 and 0.001, respectively) and in mTBI APP-SWE as compared to their WT counterparts (*P* < 0.05 and 0.0001, respectively). Two-way ANOVA showed significant differences for genotype (F_1, 12_ = 21.41, *P* = 0.0006) and for type of injury (F_1, 12_ = 5.442, *P* = 0.0379) but not for interaction (F_1, 12_ = 0.05358, *P* = 0.8208) for *Napepld*. Two-way ANOVA for *Gde1* showed a significant difference for genotype (F_1, 12_ = 82.60, *P* < 0.0001) but not for type of injury (F_1, 12_ = 0.05747, *P* = 0.8146) and interaction (F_1, 12_ = 1.707, *P* = 0.2159). On the other hand, *Faah* also increased in mTBI APP-SWE in comparison to injured WT mice (*P* < 0.05, Fig. 8C). Two-way ANOVA showed a significant difference for genotype (F_1, 12_ = 15.88, *P* = 0.0018) but not for type of injury (F_1, 12_ = 1.481, *P* = 0.2471) and interaction (F_1, 12_ = 2.298, *P* = 0.1554). These data could potentially explain the unchanged NAE levels in this tissue. Transcript levels of *Dagla* increased significantly in APP-SWE mice (*P* < 0.01 in both WT sham vs APP-SWE sham and WT mTBI vs APP-SWE mTBI). Two-way ANOVA showed significant a difference for genotype (F_1, 12_ = 45.19, *P* < 0.0001) but not for the type of injury (F_1, 12_ = 3.237, *P* = 0.0972) and interaction (F_1, 12_ = 0.1496, *P* = 0.7057). Conversely, *Magl* did not change. This result may partially explain the enhanced levels of 2-AG in the hippocampus.

Concerning the genes encoding eCBome-related receptors for the hippocampus (Fig. 8D) *Cnr1* transcript levels increased in injured APP-SWE animals (*P* < 0.05 WT mTBI vs APP-SWE mTBI). Two-way ANOVA showed significant differences for genotype (F_1, 12_ = 15.68, *P* = 0.0019) and for type of injury (F_1, 12_ = 5.303, *P* = 0.0400) but not for interaction (F_1, 12_ = 1.557, *P* = 0.2359). Whereas *Pppara* transcript levels decreased in WT mTBI and APP-SWE sham when compared to WT sham mice (*P* < 0.05 and 0.0001, respectively). Two-way ANOVA showed significant differences for interaction (F_1, 12_ = 12.86, *P* = 0.0037) and for genotype (F_1, 12_ = 35.87, *P* < 0.0001) but not for type of injury (F_1, 12_ = 1.336, *P* = 0.2703). On the other hand, *Pparg* showed an increased expression in APP-SWE animals, either when comparing APP-SWE sham to WT sham and APP-SWE mTBI to WT mTBI (*P* < 0.0001, both). Moreover, *Pparg* transcript levels increased also in APP-SWE mTBI compared to their sham mice (*P* < 0.0001). Two-way ANOVA showed significant differences for interaction (F_1, 12_ = 38.19, *P* < 0.0001), for genotype (F_1, 12_ = 362.3, *P* < 0.0001) and type of injury (F_1, 12_ = 13.79, *P* = 0.0030).

#### MALDI-MS imaging study

**Fig. 9:**
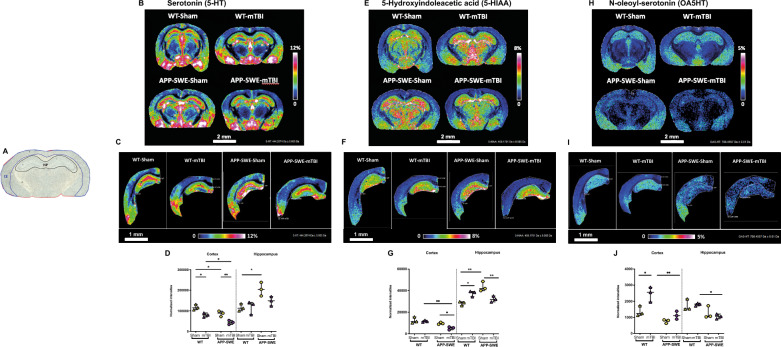
Serotonergic neurotransmitter changes induced by mTBI in APP-SWE mice compared with wild-type controls (WT). Optical image of coronal rat brain sections at bregma − 2.06 mm with brain region annotations: CE, Cortex; HP, Hippocampus (**A**). MALDI–FT–ICR–MS images of Serotonin (5-HT, **B**), 5-Hydroxyindoleacetic acid (5-HIAA, **E**) and N-oleoyl-serotonin (OA5HT, H) in coronal mouse brain tissue section of WT and APP-SWE animals without (Sham) and with mTBI. Scale bar = 2 mm. Visualization by MALDI-MSI of 5-HT (**C**), 5-HIAA (**F**) and OA5HT (**I**) in CE and HP brain regions comparing WT and APP-SWE tissues without (Sham) and with mTBI. Scale bar = 1 mm. Color scale bars are shown as a percentage of maximum intensity and were adjusted for each ion image to show a clear distribution. Data were normalized against the root-mean-square (RMS) of all data points for 5-HT and 5-HIAA and against Internal Standard OA5HT-*d*_17_ for OA5HT. Lateral resolution, 50 μm. Corresponding bar plots (mean ± s.e.m) reflecting the distribution differences of 5-HT (**D**), -HIAA (**G**) and OA5HT (**J**) between the sample groups in brain regions of interest. Statistical analysis was performed by Two-way analysis of variance (ANOVA) followed by Tukey's multiple comparison test (*P ≤ 0.05; **P ≤ 0.01). Groups: WT-Sham; WT-mTBI; APP-SWE; APP-SWE-mTBI

In order to provide a more comprehensive omics analysis, a MALDI-MSI was applied to investigate metabo-lipidomic differences between WT and APP-SWE brains (Fig. 9). In particular, we focused the study on the serotonergic pathway because of its key role in the modulation of several symptoms occurring in the early stages of AD (i.e. alterations of mood, emotive disorders, confusion, agitation and anxiety or modifications in the sleep–wake cycle). Specifically, Fig. 9A, D show MALDI-MSI images relative to the spatial distribution of serotonin (5HT) and its major metabolite (5-Hydroxyindoleacetic acid, 5-HIAA) in brain tissue sections. In this context, as shown in Fig. 9B, E, 5HT and 5-HIAA levels were significantly reduced in the cortex of APP-SWE mice. In particular, 5HT levels decreased significantly after mTBI in both WT and APP-SWE mice (*P* < 0.05 WT sham vs WT mTBI and WT mTBI vs APP-SWE mTBI, *P* < 0.01 APP-SWE sham vs APP-SWE mTBI, Fig. 9C) and decreased also in APP-SWE sham in comparison to WT sham (*P* < 0.05, Fig. 9C). Two-way ANOVA showed significant differences for genotype (F_1, 8_ = 24.30, *P* = 0.0012) and for type of injury (F_1, 8_ = 34.86, *P* = 0.0004) but not for interaction (F_1, 8_ = 0.1530, *P* = 0.7059). On the other hand, in the cortex mTBI decreased significantly 5-HIAA levels only in the APP-SWE genotype (*P* < 0.05 and 0.01 APP-SWE sham vs APP-SWE mTBI and WT mTBI vs APP-SWE mTBI, respectively, Fig. 9F). Two-way ANOVA showed significant differences for genotype (F_1, 8_ = 21.53, *P* = 0.0017) and for type of injury (F_1, 8_ = 7.055, *P* = 0.0290) but not for interaction (F_1, 8_ = 3.554, *P* = 0.0961). Additionally, in the hippocampus 5-HT levels significantly increased in APP-SWE sham in comparison to WT sham mice (*P* < 0.05), but no significant change was induced by mTBI. Two-way ANOVA showed a significant difference for genotype (F_1, 8_ = 14.19, *P* = 0.0055) but not for type of injury (F_1, 8_ = 3.091, *P* = 0.1168) and interaction (F_1, 8_ = 3.678, *P* = 0.0914). 5-HIAA levels also were found to be increased in APP-SWE sham mice in comparison to their WT counterparts (*P* < 0.01 WT sham vs APP-SWE sham), whereas mTBI induced opposite effects in WT and APP-SWE mice. In fact, in the WT mTBI group, 5-HIAA levels increased in comparison to WT sham group (*P* < 0.05) and on the contrary they decreased in APP-SWE mTBI when compared to APP-SWE sham mice (*P* < 0.01). Two-way ANOVA showed significant differences for interaction (F_1, 8_ = 32.55, *P* = 0.0005) and for genotype (F_1, 8_ = 7.970, *P* = 0.0224) but not for type of injury (F_1, 8_ = 0.3951, *P* = 0.5472). Moreover, we focused our analysis also on *N*-acylserotonins that we have identified with previously targeted lipidomics to provide spatiotemporal information on their alterations. Noteworthy, OA5HT was detected and tentatively identified by MSI using the mass information with sub-ppm mass accuracy, together with Isotopic Fine Structure (ISF) and accurate molecular formula measurements following compared to the authentic standard and the signal was finally normalized against the corresponding deuterated standard (isotopic fine structure is reported in supplementary section Figure S2) As shown in Fig. 9G, H, the trend observed in the cortex with MSI was aligned with the quantitative results provided by LC-HRMS analysis, with an increase in WT mTBI (*P* < 0.05 WT sham vs WT mTBI, Fig. 9I) and a marked decrease in APP-SWE mTBI (*P* < 0.01 WT mTBI vs APP-SWE mTBI). Two-way ANOVA showed significant differences for genotype (F_1, 8_ = 28.64, *P* = 0.0007) and for type of injury (F_1, 8_ = 16.28, *P* = 0.0038) but not for interaction (F_1, 8_ = 4.061, *P* = 0.0787). On the other hand, in the hippocampus of APP-SWE mTBI mice we observed a significant decrease in comparison to WT mTBI (*P* < 0.05). Two-way ANOVAs showed a significant difference for genotype (F_1, 8_ = 13.91, *P* = 0.0058) but not for interaction (F_1, 8_ = 0.8068, *P* = 0.3953) and type of injury (F_1, 8_ = 0.1895, *P* = 0.6748). Given the fact that 5-HT is the precursor for *N*-acyserotonin biosynthesis (but not their degradation product, since these molecules are quite stable to hydrolysis), we analyzed also the possible correlation between the levels of the neurotransmitter and those of its eCBome derivatives through a Spearman correlation analysis (Figure S4). Interestingly, in the cortex we observed a positive correlation of 5HT with EPA5HT and PA5HT (r = 1) (Figure S4 A) of WT mTBI mice, while in APP-SWE mice a negative correlation with EPA5HT (r = −1) was found in the sham group. In the APP-SWE mTBI group a negative correlation with PA5HT (r = −1) and a positive one with DHA5HT (r = 1) were also observed. On the other hand, in the hippocampus (Figure S4 B), a positive correlation with EPA5HT (r = 1) was observed in WT sham mice, while in WT mTBI mice a positive correlation with OA5HT (identified with MSI) and PA5HT (r = 1), and a negative one with DHA5HT (r = −1) were found. In the APP mice we observed only a negative correlation between 5HT and PA5HT in the mTBI group (r = −1). However, no significance was observed.

### mTBI in APP-SWE mice enhanced heterodimerization between 5HT2A and CB1 in cortex

**Fig. 10:**
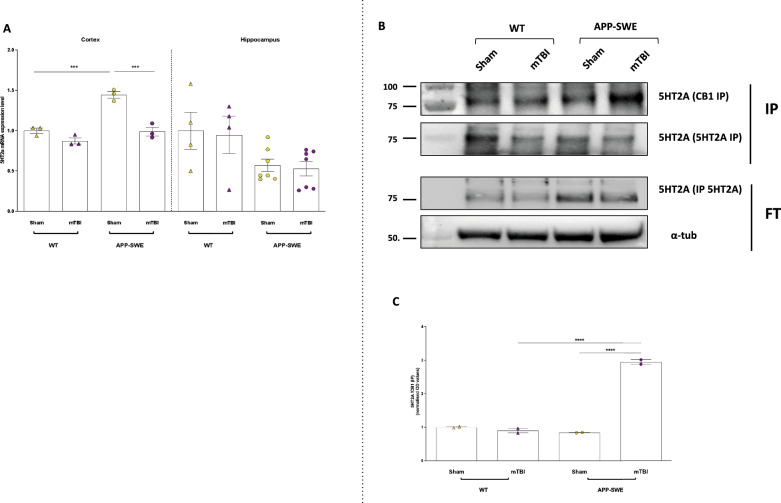
5HT2a expression and immunoprecipitation assay performed in the cortex in WT and APP-SWE mice (sham and mTBI). **A** Bar graph with individual points showing the 5HT2a mRNA expression in the cortex (left) and hippocampus (right) of wt and APP mice subjected to mTBI. **B** Representative blots showing the signals corresponding to 5HT2A protein immunoprecipitated (IP) with specific CB1 and 5HT2A antibodies. Alpha tubulin was used as an internal procedure control. The value of N = 2 arises from the pooling of two or three samples per experimental point. (C) Bar graph with individual points showing the quantification of IP results. Statistical analysis was performed by Two-way analysis of variance (ANOVA) followed by Tukey's multiple comparison test (*P ≤ 0.05; **P ≤ 0.01; ***P ≤ 0.001). Data are represented as bar graphs with individual points

Among the large family of serotonergic receptors (5-HT1 to 5-HT7, each of them presents in several isoforms), several classes appear to be implicated in AD related mechanisms. In particular, 5-HT_2A_ receptors have been reported to regulate the proteolytic cleavage of APP, neuroinflammation and cognitive deficits [24]. Remarkably, previous studies demonstrate that 5HT_2A_ and CB_1_ receptors form heterodimers in the brain whose dysfunction is implicated in the cognitive deficits produced by delta-9-tetrahydrocannabinol (D9-THC) [25]. In light of this, in this study we analysed the interaction between 5HT_2A_ and CB_1_ receptors in our experimental conditions. Before doing that, we measured transcript levels of 5HT_2A_ in the cortex and hippocampus as shown in Fig. 10A. In the cortex of wild-types, trauma did not induce significant changes, however, in sham APP-SWE mice the expression levels of 5HT_2A_ were higher than in WT sham mice (*P* < 0.001). Moreover, we observed that mTBI induced a significant decrease of 5HT_2A_ in comparison to sham mice (*P* < 0.001).

Two-way ANOVA showed significant differences for interaction (F_1, 8_ = 15.49, *P* = 0.0043), for genotype (F_1, 8_ = 45.28, *P* = 0.0001) and for type of injury (F_1, 8_ = 49.15, *P* = 0.0001).

On the contrary, in the hippocampus no significant alterations were observed. Next, we analysed the heterodimerization between CB_1_ and 5HT_2A_ in the cortex. As shown in Fig. 10B, C the immunoprecipitation analysis revealed that the 5HT_2A_ and CB_1_ receptors effectively form heteromers in the cortex of WT mice and that the expression of this complex was not significantly changed in APP-SWE sham mice, but rather in the cortex of APP-SWE mTBI mice (*P* < 0.0001 both APP-SWE mTBI vs WT mTBI and APP-SWE sham). Two-way ANOVA showed significant differences for interaction (F_1, 4_ = 505.4, *P* < 0.0043), for genotype (F_1, 4_ = 367.7, *P* < 0.0001) and type of injury (F_1, 4_ = 419.3, *P* < 0.0001).

## Discussion

Traumatic brain injury is a global health issue since it represents a chronic disease with long-term sequelae, including an increased risk of late-onset neurodegeneration. Compelling data from several studies have suggested the association between TBI and AD [3, 4], however there is still the need for a better understanding of the underlying molecular mechanisms. Our study aimed to investigate the behavioural and biomolecular effects of mild TBI in a transgenic model of AD at a presymptomatic stage, in order to identify potential changes that occur in the early stages of the amyloidogenic process, prior, and potentially contributing, to amyloid plaque formation and cognitive impairment.

Our previous studies [11, 26–29] extensively described the long-term behavioural features of mice with mTBI, and suggested a characteristic biphasic phenotype in these animals (early aggressive and late depressed), as similarly reported in humans (DSM V). Here, we showed that mTBI causes cognitive deficits with impairments in spatial and recognition memory 60 days post injury. However, it did not affect cognitive abilities in APP-SWE mice. As they were tested in a presymptomatic stage (6 months), APP-SWE sham mice did not exhibit significant impairments in terms of cognition [30] as compared with WT littermates. However, they exhibited anxiety and increased locomotor activity, as shown by the reduced time spent in the center in the open field, together with an enhanced travelled distance. Moreover, they showed aggressive behaviour regardless of the trauma. Even though we cannot exclude that any behavioural change caused by the trauma might be masked by the basal restless phenotype, overall, our data indicate no substantial behavioural differences, in terms of cognitive functioning, between sham and mTBI groups under the APP-SWE genotype.

On the other hand, biochemical analyses revealed that mTBI increased Aβ_1-42_ levels in the cortex in APP-SWE, but not in WT mice. Though the mTBI-induced cognitive impairment in WT mice was not relatable to the Aβ_1-42_ amount, our data may suggest that brain trauma can affect AD progression in genetically predisposed animals. In agreement, we found elevated cortical levels of BACE1, the enzyme responsible for APP cleavage and production of Aβ peptides, in traumatized APP-SWE mice. Therefore, the early formation of the insoluble Aβ “proto-fibrils'' [31], triggered by the increased activity of BACE1, could be exacerbated by the trauma. Interestingly, mTBI did not affect BACE1 expression in wild-types, which supports the hypothesis of a predisposing effect, which in itself, under a WT genotype, is not sufficient to develop dementia, at least from a molecular point of view. This potential predisposing effect of mTBI appeared to be mostly concerned with the cortex and, surprisingly, was not observed the hippocampus, in agreement with the lack of any behavioural consequence in these presymptomatic mice, and possibly suggesting that such putative effect might involve cortical circuitries linked with the hippocampus, or cortical functions not normally affected in symptomatic and uninjured APP-SWE mice. However, further experiments will be necessary to identify the behavioural long-term consequences of the trauma in transgenic AD mice with full-blown pathology (about 9–10 months) to ascertain a possible worsening of the disease.

In view of the inflammatory nature of AD, we also investigated immune-inflammatory processes, which are crucial events driving TBI pathophysiology. The early neuroinflammation mediated by glia cell activation in TBI represents a beneficial mechanism, which stimulates an anti-inflammatory response to the damage [32]. Indeed, several studies [11, 12, 33, 34] detected increased levels of IL-1β in cortical areas in injured mice a few days following damage. However, brain blood barrier (BBB) damage is associated with the movement of immune cells and inflammatory molecules leading to a systemic inflammatory condition. Here we detected an elevation in peripheral levels of some pro-inflammatory cytokines 60 days following the trauma, which may play a role in the long-term neurological sequelae of brain injury. Specifically, mTBI increased the levels of IL-1β and TNFα in WT mice, whereas APP-SWE injured mice showed an increase in IL-1β and IL-17A expression.

As a major mediator of brain damage [35, 36], circulatory IL-1β levels were increased by the trauma in traumatized mice compared to the related sham animals. In addition, a significant correlation between the APP strain and high blood IL-1β levels was observed. Conversely, the role of TNF-α is well established in AD pathogenesis [37]. Pre-clinical and clinical studies support the involvement of peripheral and central TNF-α in AD, and elevated TNF-α levels were found in the serum [38, 39] and postmortem brains of AD patients and animal models [40, 41]. However, our data showed increased blood TNF-α levels in WT without affecting transgenic mice even though an increasing trend in APP-SWE was observed. In any case, we can speculate that brain trauma did not modulate TNF-α in APP mice, at least at this presymptomatic stage. Similar to TNF-α and IL-1β, the expression of IL-17 has been correlated to changes in the upstream inflammatory signalling factor IL-23 in secondary brain injury after TBI [42]. Indeed, increased IL-17 levels have been detected in the serum of TBI rats a few days after injury [42]. Interestingly, the pathogenic role of IL-17 in promoting synaptic dysfunction and cognitive and memory deficit in AD has been recently suggested [43]. Noteworthy, we detected the late (60 days, when the behavioural traits are still not visible) overexpression of IL-17 only in APP-SWE mTBI mice. This finding may support the concept that the pathophysiological dysregulation of this cytokine may promote neurodegeneration, and support the hypothesis that mTBI might exacerbate some late behavioural dysfunctions in eventually symptomatic APP-SWE mice.

**Fig. 11:**
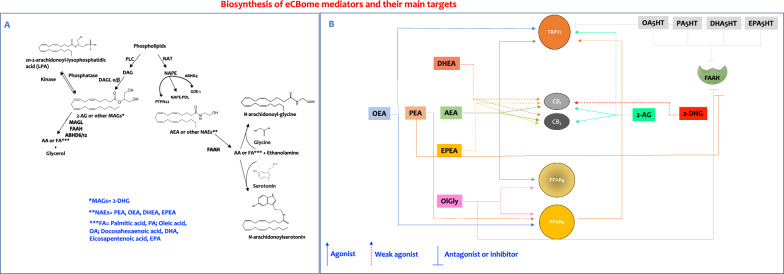
Scheme for biosinthesis of the endocannabinoidome mediators (**A**) and their main targets (**B**). PLC, phospholipase C; DAG, diacylglycerol; DAGLα/β, *sn*-1-specific diacylglycerol lipase-α or β; MAGs, monoacylglycerols; 2-AG, 2-arachidonoylglycerol; NAT, N-acyltransferase (including phospholipase A2 group IVE and phospholipase A/acyltransferase; ABHD4, α/β-hydrolase 4; PTPN22, tyrosine-protein phosphatase non-receptor type 22; *N*-acyl-phosphatidylethanolamine-hydrolysing phospholipase D (NAPE-PLD); GDE1, glycerophosphodiester phosphodiesterase 1; AEA, anandamide; NAEs, *N*-acylethanolamines; FAAH, fatty acid amide hydrolase; MAGL, monoacylglycerol lipase; ABHD6/12, α/β-hydrolase 6/12; FA, fatty acid; CB, cannabinoid receptor; PPAR, peroxisome proliferator-activated receptor; TRPV1, transient receptor potential cation channel subfamily V member 1; OEA, *N*-oleoylethanolamine; PEA, *N*-palmitoylethanolamine; DHEA, *N*-docosahexaenoyl ethanolamine; EPEA, *N*-eicosapentaenoyl ethanolamine; 2-DHG, 2-docosahexaenoyl glycerol; OlGly, *N*-oleoylglycine; OA5HT,* N*-oleoylserotonin; PA5HT*, N*-palmitoylserotonin DHA5HT, *N*-docosahexaenoylserotonin and EPA5HT*, N*-eicosapentaenoylserotonin

The present study is the first investigating the effect of mTBI and APP genotype on eCBome signalling. For a better understanding of the system and to facilitate the reading of this section, we included a scheme summarising the biosynthesis and molecular targets of the eCBome mediators investigated in the present study (Fig. 11).

Our data show a significant alteration of 2-AG, which is up-regulated in both the cortex and hippocampus of pre-symptomatic APP-SWE mice, with trauma inducing a significant increase in this mediator when compared with sham controls. Increased levels of 2-AG might act as a neuroprotective mechanism to reduce BACE1 and Aβ production, possibly via PPARɣ activation, as previously reported in 5XFAD transgenic (TG) mice by pharmacological inhibition of MAGL [44, 45]. In particular, in the mentioned paper, the authors observed a reduction in BACE1 and Aβ production by the intraperitoneal (i.p.) administration of the MAGL inhibitor ZL184 (12 mg/kg) 3 times per week starting at 2 months of age for 16 weeks or starting at 4 months of age for 8 weeks, and they observed the effects at 6 months of age. They observed also a CB_1_ and CB_2_ independent mechanism, with an involvement of PPARɣ and our data showed an increase in the expression of this receptor in both cerebral areas analysed. Conversely, 2-AG might also contribute to the pathogenesis and the progression of the disease, as evidence suggests that in neurological disorders, eCBs may no longer be tightly regulated but become dysregulated and contribute to the pathological state and/or in its symptoms in different ways depending on the location and timing of their production and on the stage of the disease [7]. Very interestingly, the correlation analysis showed that both 2-AG and EPEA, an omega-3 fatty acid-derived NAE with proposed anti-inflammatory activity, are related to levels of Aβ_1−42_ in cortex and, in particular, EPEA negatively correlated with Aβ_1−42_ in APP-SWE injured mice. We have previously shown in the same mouse model of AD that neither EPEA, nor other NAEs, did change significantly during the symptomatic stage (12–15 months) in the cortex [46], nor after 60 days post-mTBI in adult C57BL/6 mice [12]. In the literature several papers have reported the beneficial role of ethanolamine plasmalogens (PlsEtns), a subtype of phospholipids that are involved in the biosynthesis of NAE-related eCBs [47], and have a close association with AD [48]. Decreased levels of PlsEtns have been commonly found in AD patients, and were correlated with impairment of cognitive function and severity of disease, whereas EPA-enriched ethanolamine plasmalogens (EPA-pPE) significantly improve cognition in an AD mouse model by suppressing β-amyloid generation [49]. Therefore, the decreased levels of EPEA, a potential product of EPA-pPE, in the cortex of injured APP-SWE mice might be related with reduced availability of EPEA biosynthetic precursors and with an accelerated AD-like phenotype, due to their negative correlation with Aβ_1−42_. In addition, albeit not significantly for most NAEs, AEA, PEA, OEA and EPEA showed the same trend in the cortex, suggesting a common behaviour and response to the disease. Interestingly, again in the cortex, the transcriptomic analysis of genes involved in the metabolism of eCBs suggested an increased tone of 2-AG, but also an increased biosynthesis of NAEs. However, if some of the biosynthetic precursors of NAEs are less available, as it might happen for EPEA, the increased gene expression of biosynthetic enzymes would be not enough to increase their endogenous levels, and might nevertheless represent an adaptive mechanism attempting to reduce the consequences of decreased amounts of NAE biosynthetic precursors. In the hippocampus, the unchanged NAE levels could be related to differential gene expression of their biosynthetic and degradative enzymes, because our data suggest the existence of a balance between the catabolic and metabolic routes. Also the increased levels of 2-AG might be related with increased expression of *Dagla* in the APP-SWE mTBI group with no change in *Magl* expression. Moreover, in this brain area we found an increase of the transcript levels of CB_1_ in APP-SWE mice compared to their WT, with no change induced by trauma, which might be correlated with the increase of 2-AG. In addition, mTBI enhanced significantly OlGly levels in APP-SWE animals, which could be correlated with the increased expression of the gene coding for *Pparg*, since this compound is able to activate both PPARɑ [27] and PPARɣ [50]. Very interestingly, our data showed a decrease of the gene expression levels of *Ppara* in APP-SWE mice, which could be correlated with reduced signalling of PEA (a PPARɑ agonist), and an increase of *Pparg*. These results are in line with the established role of these nuclear receptors, since PPARɣ is reported to regulate lipogenesis and insulin sensitization [51] but also to play anti-inflammatory actions [52–55], whereas PPARɑ controls the expression of genes encoding key enzymes of fatty acid oxidation [56] and modulates amyloid metabolism by activating the non-amyloidogenic ɑ-secretase, while inhibiting the amyloidogenic β-secretase [57, 58]. Moreover, recently, PPARɑ expression and transcriptional activity have been found to correlate with the expression of APP [59]. Therefore, the upregulation of PPARɣ induced by mTBI might be a mechanism to promote insulin sensitivity and enhance glucose metabolism; on the other hand, the downregulation of PPARɑ might outline a reduced triglyceride metabolism with a subsequent overall dysregulation in energy homeostasis that might contribute to the development of AD induced by brain trauma in genetically predisposed mice. Given the anti-neuroinflammatory actions of both these nuclear receptors [55, 60, 61], their opposing changes in presymptomatic APP-SWE mice might play, respectively, a protective and exacerbating role in the development of the neuroinflammatory disorder.

Here, we have also demonstrated for the first time that *N*-acylserotonins, a lipid class that belongs to the expanded eCB system and play a role as antagonists of TRPV1 channels (an eCBome receptor) and/or FAAH inhibitors [62], and identified so far only in intestinal tissues [63], are produced also in the brain and are altered after mTBI. In particular, in the cortex the oleoyl-derivative, OA5HT, increased significantly after mTBI in wild-types but not in APP-SWE mice, whereas in the hippocampus mTBI enhanced OA5HT levels in AD mice suggesting a production probably depending on the site and on the type and stage of the disease. We also identified the DHA-derived *N*-acyl-serotonin, DHA5HT, and the correlation analysis suggested that this anti-inflammatory mediator may modulate the release of inflammatory cytokines in the plasma. Interestingly, we observed a modulation of PA5HT induced by trauma in both WT and APP-SWE mice in the hippocampus, that could be correlated with 5HT levels, as shown in the Spearman analysis. This mediator, which showed a remarkable protective effect against glutamate-induced cytotoxicity and oxidative stress, and markedly suppressed the glutamate-induced activation of ERK in the late phase, is a dual antagonist of FAAH and TRPV1 [64, 65], and was also tested in an in vivo model of Parkinson’s disease to investigate its effects on L-3,4-dihydroxyphenylalanine (L-DOPA)-induced dyskinesia (LID), which is a common side effect of the long-term treatment with L-DOPA. PA5HT effectively attenuated the development of LID by decreasing dopamine D1 receptor-hyperactivation [65]. However, the authors did not investigate the endogenous modulation of PA5HT levels in the brain. Our data showed that these mediators are significantly altered by the injury and also in the AD mouse model, which might be useful in the development of novel therapeutic targets. Moreover, we have previously reported that in an experimental model of antibiotic-induced dysbiosis there was a significant decrease in the intestinal levels of some *N*-acylserotonins [17]. We hypothesised that, in view of the fact that circulating long chain *N*-acylserotonins can cross the BBB and are resistant to hydrolysis to serotonin [66], their decrease might contribute to both intestinal inflammation and depression-like symptoms observed in antibiotic-induced dysbiosis [17].

Evidence for an interplay between the eCB and the serotonin signalling systems has been suggested by many studies showing a high level of functional overlap and interaction between these two systems [67–70]. Therefore, we employed a MALDI-MS imaging approach to visualize the spatial distribution of multiple neuromodulators directly across different brain regions in mouse brain tissue sections from WT and APP-SWE mice, with the aim to better understand the possible interplay between serotonin and the eCBome, beyond the role of the neurotransmitter in generating the aforementioned *N*-acyl-serotonins. Our data demonstrate that 5-HT and 5-HIAA are regulated in the same manner and depending on the brain area analysed. We speculate that, while in the cortex 5-HT and 2-AG are inversely correlated, in the hippocampus there seems to be a positive association. Indeed, previous evidence supported the hypothesis that eCBs, phytocannabinoids and/or synthetic cannabinoids are able to increase or decrease 5-HT levels depending on the brain region, ligand and dose [71]. Moreover, our MALDI-MSI approach was able to localise for the first time OA5HT in the brain and obtain similar quantitative results as with LC-HRMS, at least for the cortex. The different result obtained in the hippocampus by using the two techniques might be due to the different preparation of the samples, which, in the case of MALDI-MSI, avoids tissue homogenization, extraction and purification, thus preserving tissue integrity, while, in the case of LC-HRMS, conventional procedures of organic extractions are needed to enrich the purified fraction in a specific lipid class and improve sensitivity. Although MSI allowed to investigate the spatiotemporal behaviour of the molecule OA5HT within the histological compartments of the brain structures, this type of analysis of the mediator may have been biased by the reduced detection sensitivity and the lower derivatization efficiency on the phenol group instead of the primary amine [18]. Therefore, the result should certainly be verified on a larger cohort of subjects, also considering the biological variability of the experimental model. Finally, we demonstrated that the transcript levels of 5HT_2A_ in the cortex underwent alterations similar to 2-AG levels, while in the hippocampus no change occurred, possibly suggesting that, at least in the cortex, 2-AG is able to modulate 5HT_2A_ expression or viceversa. Moreover, the IP showed that heterodimerization between CB_1_ and 5HT_2A_ occurs in the cortex, for the first time in a manner not induced by a pharmacological activation. The increased tone of 2-AG in APP-SWE mice might have driven the formation of the heteromers. Indeed, evidence in literature supports the hypothesis that this type of receptor interaction mediates the cognitive impairment induced by THC [72]. Thus, the formation and enhancement of the heterodimers induced by the trauma (possibly via 2-AG) in the transgenic AD mouse might be associated with later cognitive impairment. Further investigations are needed to assess this hypothesis, although our data suggest that a pivotal role might be played by this interplay. While this study primarily focused on CB_1_ and its interactions with 5-HT_2A_ receptors, substantiated by existing literature and our findings, future research should also investigate CB_2_ in this context. Expanding the scope to include this receptor will provide a more comprehensive understanding of the role of the eCB system in this pathological condition.

In conclusion, the present study demonstrates that mild brain trauma might induce and/or exacerbate some biochemical hallmarks of AD in genetically predisposed animals. The brain eCBome is differentially modulated by both mTBI and the presence of an asymptomatic AD-like genotype in a brain region-dependent manner. The serotonergic system, through serotonin and its 5HT_2A_ receptors, influences the signalling of the eCBome by either participating in the formation of *N*-acyl-serotonins or forming heteromers with CB_1_ receptors, thus providing potentially novel targets for the development of new therapeutic tools against AD and the consequences of mTBI.

### Supplementary Information

Additional file 1.

Additional file 2.

Additional file 3.

Additional file 4.

Additional file 5.

Additional file 6.

Additional file 7.

## Data Availability

The datasets used and/or analysed during the current study are available from the corresponding author on reasonable request.
