# Supplementary material for: Early biomarkers in the presymptomatic phase of cognitive impairment: changes in the endocannabinoidome and serotonergic pathways in Alzheimer's-prone mice after mTBI
Source: Acta Neuropathol Commun. 2024 Jul 12;12:113. doi: 10.1186/s40478-024-01820-0 (PMC11241935; doi:10.1186/s40478-024-01820-0)
Supplement: Supplementary file 1 — Additional file 1. [file 40478_2024_1820_MOESM1_ESM.docx]

**Additional information**

**Title: Early biomarkers in the presymptomatic phase of cognitive impairment: changes in the endocannabinoidome and serotonergic pathways in Alzheimer's-prone mice after mTBI**

Francesca Guida^1^, Monica Iannotta^1^, Anna Lauritano^2^, Rosmara Infantino^1^, Emanuela Salviati^3^, Roberta Verde^2^, Livio Luongo^1^, Eduardo Maria Sommella^3^, Fabio Arturo Iannotti^2^, Pietro Campiglia^3^, Sabatino Maione^1^, Vincenzo Di Marzo^2,4†*^ and Fabiana Piscitelli^2†*^.

^1^ Department of Experimental Medicine, Pharmacology Division, University of Campania "L. Vanvitelli", Naples, Italy^; 2^ Endocannabinoid Research Group, Institute of Biomolecular Chemistry (ICB), National Research Council (CNR), Pozzuoli (NA), Italy; ^3^ Università degli studi di Salerno, Dipartimento di Farmacia, Italy, Fisciano (SA); ^4^ Institut Universitaire de Cardiologie et de Pneumologie de Québec and Institut sur la Nutrition et les Aliments Fonctionnels, Université Laval, Quebec City, Canada

**Additional file 1**

**Table S1.** List of neurotransmitters acquired by MALDI FT-ICR-MSI from mouse brain tissue sections.

File format: .txt

| **Compound** | **Molecular Formula** | **Theoretical m/z** | **Observed m/z** | **Mass accuracy (ppm)** |
| --- | --- | --- | --- | --- |
| 5-HT + FMP-10 ^+^ | C_30_H_26_N_3_O^+^ | 444.2070 | 444.2074 | 0.90 |
| 5-HIAA + FMP-10 ^+^ | C_30_H_23_N_2_O_3_^+^ | 459.1703 | 459.1701 | -0.44 |
| OA5HT + FMP-10 ^+^ | C_48_H_58_N_3_O_2_^+^ | 708.4523 | 708.4557 | 4.79 |
| OA5HT + FMP-10 ^+^ | C_48_H_41_D_17_N_3_O_2_^+^ | 725.5590 | 725.5582 | -0.14 |

**Table S2.** Table reporting details for statistical analysis and normality distribution tests for behavioural and biochemical analysis

File format: .pdf

**Additional figures**

**Figure S1**. Spearman’s correlation coefficient for Aβ_1−42_ and 2-AG and EPEA levels in the cortex for WT (A) and APP-SWE (B) mice.

**Figure S2**. Spearman’s correlation coefficient for DHA5HT in hippocampus (HP) and cortex (CE) with plasmatic cytokines for WT (A) and APP-SWE (B) mice.

**Figure S3.** Tentative identification of N-oleoyl serotonin (OA5HT) and Internal Standard OA5HT-*d_17_* by MSI using the mass information with sub-ppm mass accuracy and the Isotopic Fine Structure (ISF) provided by the FT-ICR-MS platform.

**Figure S4**. Spearman’s correlation coefficient for serotonin (5HT) with the identified *N*-acylserotonins in the cortex (A) and (B) hippocampus.

**Figure S5**. Procedural controls. (A) Representative blot showing the 5HT2A expression in the cortex (not immunoprecipitated, Input). (B) Normal IgG used as an isotype control and loaded on gel

Files format: .pdf
