# Supplementary material for: Early biomarkers in the presymptomatic phase of cognitive impairment: changes in the endocannabinoidome and serotonergic pathways in Alzheimer's-prone mice after mTBI
Source: Acta Neuropathol Commun. 2024 Jul 12;12:113. doi: 10.1186/s40478-024-01820-0 (PMC11241935; doi:10.1186/s40478-024-01820-0)
Supplement: Supplementary file 2 — Additional file 2. [file 40478_2024_1820_MOESM2_ESM.pdf]

1 2way ANOVA of stat Rit Frequency 15 D intergroup

**Table Analyzed**	stat Rit Frequency 15 D				
**Two-way ANOVA**	Ordinary				
**Alpha**	0,05				
**Source of Variation**	% of total variation	P value	P value summary	Significant?	
**Interaction**	11,25	0,0163	*	Yes	
**genotype**	36,79	<0,0001	****	Yes	
**injury**	3,712	0,1533	ns	No	
**ANOVA table**	SS	DF	MS	F (DFn, DFd)	P value
**Interaction**	69,03	1	69,03	F (1, 28) = 6,527	P=0,0163
**genotype**	225,8	1	225,8	F (1, 28) = 21,35	P<0,0001
**injury**	22,78	1	22,78	F (1, 28) = 2,154	P=0,1533
**Residual**	296,1	28	10,58		
**Difference between column means**					
**Mean of sham**	6,750				
**Mean of mTBI**	8,438				
**Difference between means**	-1,688				
**SE of difference**	1,150				
**95% CI of difference**	-4,043 to 0,6677				
**Difference between row means**					
**Mean of wt**	4,938				
**Mean of app**	10,25				
**Difference between means**	-5,313				
**SE of difference**	1,150				
**95% CI of difference**	-7,668 to -2,957				
**Interaction CI**					
**Mean diff, A1 - B1**	-4,625				
**Mean diff, A2 - B2**	1,250				
**(A1 -B1) - (A2 - B2)**	-5,875				
**95% CI of difference**	-10,59 to -1,165				
**(B1 - A1) - (B2 - A2)**	5,875				
**95% CI of difference**	1,165 to 10,59				
**Normality of Residuals**					
**Test name**	Statistics	P value	Passed normality test (alpha=0,05)?	P value summary	
**D'Agostino-Pearson omnibus (K2)**	33,12	<0,0001	No	****	
**Anderson-Darling (A2*)**	1,691	0,0002	No	***	
**Shapiro-Wilk (W)**	0,8018	<0,0001	No	****	
**Kolmogorov-Smirnov (distance)**	0,2166	0,0005	No	***	
**Data summary**					
**Number of columns (injury)**	2				

**Number of rows (genotype)**	2				
**Number of values**	32				

	WT-sham	WT-mTBI	APP-sham	APP-mTBI
Test for normal distribution				
D'Agostino & Pearson test				
K2	10,93	4,078	1,235	1,925
P value	0,0042	0,1302	0,5393	0,3819
Passed n	No	Yes	Yes	Yes
P value su	**	ns	ns	ns
Anderson-Darling test				
A2*	1,175	0,4708	0,4	0,6169
P value	0,0019	0,1744	0,2736	0,069
Passed n	No	Yes	Yes	Yes
P value su	**	ns	ns	ns
Shapiro-Wilk test				
W	0,6756	0,868	0,894	0,8443
P value	0,0012	0,1439	0,2547	0,0834
Passed n	No	Yes	Yes	Yes
P value su	**	ns	ns	ns
Kolmogorov-Smirnov test				
KS distan	0,3472	0,2602	0,2224	0,2372
P value	0,0052	>0,1000	>0,1000	>0,1000
Passed n	No	Yes	Yes	Yes
P value su	**	ns	ns	ns
Number of	8	8	8	8

2. 2way ANOVA of stat Rit Duration 15 D intergroup

**Table Analyzed**	stat Rit Duration 15 D				
**Two-way ANOVA**	Ordinary				
**Alpha**	0,05				
**Source of Variation**	% of total variation	P value	P value summary	Significant?	
**Interaction**	21,01	0,0066	**	Yes	
**WT/APP**	0,9237	0,5437	ns	No	
**Model**	9,630	0,0570	ns	No	
**ANOVA table**	SS	DF	MS	F (DFn, DFd)	P value
**Interaction**	2493	1	2493	F (1, 28) = 8,594	P=0,0066
**WT/APP**	109,6	1	109,6	F (1, 28) = 0,3779	P=0,5437
**Model**	1143	1	1143	F (1, 28) = 3,940	P=0,0570
**Residual**	8120	28	290,0		
**Difference between column means**					
**Mean of sham**	28,57				
**Mean of mTBI**	40,53				
**Difference between means**	-11,95				
**SE of difference**	6,021				
**95% CI of difference**	-24,28 to 0,3821				
**Difference between row means**					
**Mean of wt**	32,70				
**Mean of app**	36,40				
**Difference between means**	-3,701				
**SE of difference**	6,021				
**95% CI of difference**	-16,03 to 8,632				
**Interaction CI**					
**Mean diff, A1 - B1**	-29,60				
**Mean diff, A2 - B2**	5,700				
**(A1 -B1) - (A2 - B2)**	-35,30				
**95% CI of difference**	-59,97 to -10,64				
**(B1 - A1) - (B2 - A2)**	35,30				
**95% CI of difference**	10,64 to 59,97				
**Data summary**					
**Number of columns (Model)**	2				

**Number of rows (WT/APP)**	2				
**Number of values**	32				

	WT-sham	WT-mTBI	APP-sham	APP-mTBI
Test for normal distribution				
D'Agostino & Pearson test				
K2	1,906	0,6978	2,644	0,6657
P value	0,3856	0,7055	0,2666	0,7169
Passed n	Yes	Yes	Yes	Yes
P value su	ns	ns	ns	ns
Anderson-Darling test				
A2*	0,3602	0,1865	0,7888	0,2527
P value	0,3497	0,8598	0,0229	0,6276
Passed n	Yes	Yes	No	Yes
P value su	ns	ns	*	ns
Shapiro-Wilk test				
W	0,9112	0,9593	0,8078	0,9436
P value	0,3628	0,8034	0,0346	0,6463
Passed n	Yes	Yes	No	Yes
P value su	ns	ns	*	ns
Kolmogorov-Smirnov test				
KS distan	0,2374	0,1518	0,3295	0,1733
P value	>0,1000	>0,1000	0,0108	>0,1000
Passed n	Yes	Yes	No	Yes
P value su	ns	ns	*	ns
Number of	8	8	8	8

3. 2way ANOVA of stat Rit Latency 15 D intergroup

**Table Analyzed**	stat Rit Latency 15 D				
**Two-way ANOVA**	Ordinary				
**Alpha**	0,05				
**Source of Variation**	% of total variation	P value	P value summary	Significant?	
**Interaction**	10,54	0,0063	**	Yes	
**wt or ko**	26,99	<0,0001	****	Yes	
**tipe of injury**	28,64	<0,0001	****	Yes	
**ANOVA table**	SS	DF	MS	F (DFn, DFd)	P value
**Interaction**	10706	1	10706	F (1, 28) = 8,725	P=0,0063
**wt or ko**	27407	1	27407	F (1, 28) = 22,34	P<0,0001
**tipe of injury**	29083	1	29083	F (1, 28) = 23,70	P<0,0001
**Residual**	34354	28	1227		
**Difference between column means**					
**Mean of sham**	100,7				
**Mean of mTBI**	40,40				
**Difference between means**	60,29				
**SE of difference**	12,38				
**95% CI of difference**	34,93 to 85,66				
**Difference between row means**					
**Mean of wt**	99,81				
**Mean of app**	41,28				
**Difference between means**	58,53				
**SE of difference**	12,38				
**95% CI of difference**	33,16 to 83,90				
**Interaction CI**					
**Mean diff, A1 - B1**	96,88				
**Mean diff, A2 - B2**	23,71				
**(A1 -B1) - (A2 - B2)**	73,16				
**95% CI of difference**	22,43 to 123,9				
**(B1 - A1) - (B2 - A2)**	-73,16				
**95% CI of difference**	-123,9 to -22,43				
**Normality of Residuals**					
**Test name**	Statistics	P value	Passed normality test (alpha=0,05)?	P value summary	
**D'Agostino-Pearson omnibus (K2)**	1,498	0,4728	Yes	ns	
**Anderson-Darling (A2*)**	1,029	0,0090	No	**	
**Shapiro-Wilk (W)**	0,9385	0,0679	Yes	ns	
**Kolmogorov-Smirnov (distance)**	0,1770	0,0122	No	*	
**Data summary**					
**Number of columns (tipe of injury)**	2				

**Number of rows (wt or ko)**	2				
**Number of values**	32				

	WT-sham	WT-mTBI	APP-sham	APP-mTBI
Test for normal distribution				
D'Agostino & Pearson test				
K2	0,5863	1,455	0,7054	2,755
P value	0,7459	0,4831	0,7028	0,2522
Passed n	Yes	Yes	Yes	Yes
P value su	ns	ns	ns	ns
Anderson-Darling test				
A2*	0,4308	0,3399	0,1648	0,8397
P value	0,2254	0,3956	0,906	0,0166
Passed n	Yes	Yes	Yes	No
P value su	ns	ns	ns	*
Shapiro-Wilk test				
W	0,9164	0,9204	0,9663	0,7877
P value	0,4013	0,4328	0,8677	0,0211
Passed n	Yes	Yes	Yes	No
P value su	ns	ns	ns	*
Kolmogorov-Smirnov test				
KS distan	0,2712	0,2095	0,1407	0,2852
P value	0,0853	>0,1000	>0,1000	0,0543
Passed n	Yes	Yes	Yes	Yes
P value su	ns	ns	ns	ns
Number of	8	8	8	8

4. 2way ANOVA of Y-maze 30 d and 60 d intergroup

**Table Analyzed**	Y-maze 30 d and 60 d				
**Two-way RM ANOVA**	Matching: Stacked				
**Assume sphericity?**	Yes				
**Alpha**	0,05				
**Source of Variation**	% of total variation	P value	P value summary	Significant?	
**Time x Column Factor**	4,856	0,4036	ns	No	
**Time**	0,1928	0,7315	ns	No	
**Column Factor**	1,729	0,8007	ns	No	
**Subject**	48,29	0,4251	ns	No	
**ANOVA table**	SS	DF	MS	F (DFn, DFd)	P value
**Time x Column Factor**	392,9	3	131,0	F (3, 28) = 1,009	P=0,4036
**Time**	15,60	1	15,60	F (1, 28) = 0,1202	P=0,7315
**Column Factor**	139,9	3	46,63	F (3, 28) = 0,3342	P=0,8007
**Subject**	3908	28	139,6	F (28, 28) = 1,075	P=0,4251
**Residual**	3636	28	129,9		
**Difference between row means**					
**Mean of 30**	64,32				
**Mean of 60**	65,31				
**Difference between means**	-0,9875				
**SE of difference**	2,849				
**95% CI of difference**	-6,823 to 4,848				
**Data summary**					
**Number of columns (Column Factor)**	4				
**Number of rows (Time)**	2				
**Number of subjects (Subject)**	32				
**Number of missing values**	0				

	WT-sham 3	WT-Sham 6	WT-mTBI 3(	WT-mTBI 6(	APP-Sham :	APP-Sham (	APP-mTBI 3	APP-mTBI 6
Test for normal distribution								
D'Agostino & Pearson test								
K2	2,679	0,6738	2,753	1,42	1,319	2,383	0,7214	1,175
P value	0,262	0,714	0,2525	0,4917	0,5172	0,3037	0,6972	0,5558
Passed n(	Yes	Yes	Yes	Yes	Yes	Yes	Yes	Yes
P value sı	ns	ns	ns	ns	ns	ns	ns	ns
Anderson-Darling test								
A2*	0,4159	0,2514	0,4396	0,4711	0,2226	0,6829	0,2055	0,412
P value	0,2476	0,6321	0,2132	0,174	0,7403	0,0452	0,8011	0,2539
Passed n(	Yes	Yes	Yes	Yes	Yes	No	Yes	Yes
P value sı	ns	ns	ns	ns	ns	*	ns	ns
Shapiro-Wilk test								
W	0,8865	0,9564	0,905	0,8644	0,9669	0,8191	0,955	0,8988
P value	0,2169	0,7755	0,3203	0,1328	0,873	0,0457	0,7615	0,2817
Passed n(	Yes	Yes	Yes	Yes	Yes	No	Yes	Yes
P value sı	ns	ns	ns	ns	ns	*	ns	ns
Kolmogorov-Smirnov test								
KS distan	0,2234	0,1972	0,2372	0,205	0,189	0,2824	0,1502	0,2376
P value	>0,1000	>0,1000	>0,1000	>0,1000	>0,1000	0,0597	>0,1000	>0,1000
Passed n(	Yes	Yes	Yes	Yes	Yes	Yes	Yes	Yes
P value sı	ns	ns	ns	ns	ns	ns	ns	ns
Number of '	8	8	8	8	8	8	8	8

5. 2way ANOVA of Y-maze FORCED_latency 30-60D intergroup

**Table Analyzed**	Y-maze FORCED_latency 30-60D				
**Two-way RM ANOVA**	Matching: Stacked				
**Assume sphericity?**	Yes				
**Alpha**	0,05				
**Source of Variation**	% of total variation	P value	P value summary	Significant?	
**Time x Column Factor**	27,93	<0,0001	****	Yes	
**Time**	0,2183	0,5902	ns	No	
**Column Factor**	24,11	0,0004	***	Yes	
**Subject**	27,15	0,2345	ns	No	
**ANOVA table**	SS	DF	MS	F (DFn, DFd)	P value
**Time x Column Factor**	4072	3	1357	F (3, 28) = 12,66	P<0,0001
**Time**	31,82	1	31,82	F (1, 28) = 0,2968	P=0,5902
**Column Factor**	3514	3	1171	F (3, 28) = 8,287	P=0,0004
**Subject**	3958	28	141,4	F (28, 28) = 1,319	P=0,2345
**Residual**	3002	28	107,2		
**Difference between row means**					
**Mean of 30**	11,88				
**Mean of 60**	13,29				
**Difference between means**	-1,410				
**SE of difference**	2,589				
**95% CI of difference**	-6,713 to 3,892				
**Data summary**					
**Number of columns (Column Factor)**	4				
**Number of rows (Time)**	2				
**Number of subjects (Subject)**	32				
**Number of missing values**	0				

	WT-sham 3	WT-Sham 6	WT-mTBI 3(	WT-mTBI 6(	APP-Sham :	APP-Sham (	APP-mTBI 3	APP-mTBI 6
Test for normal distribution								
D'Agostino & Pearson test								
K2	12,12	16,87	3,586	1,328	12,34	6,566	8,02	21,51
P value	0,0023	0,0002	0,1665	0,5149	0,0021	0,0375	0,0181	<0,0001
Passed n(	No	No	Yes	Yes	No	No	No	No
P value su	**	***	ns	ns	**	*	*	****
Anderson-Darling test								
A2*	0,7332	1,231	0,6284	0,3612	0,8482	1,483	0,6157	1,755
P value	0,0327	0,0014	0,0641	0,3477	0,0157	0,0003	0,0696	<0,0001
Passed n(	No	No	Yes	Yes	No	No	Yes	No
P value su	*	**	ns	ns	*	***	ns	****
Shapiro-Wilk test								
W	0,7884	0,6567	0,8216	0,8952	0,7766	0,6301	0,8317	0,5361
P value	0,0215	0,0007	0,0485	0,2616	0,016	0,0004	0,0619	<0,0001
Passed n(	No	No	No	Yes	No	No	Yes	No
P value su	*	***	*	ns	*	***	ns	****
Kolmogorov-Smirnov test								
KS distan	0,2417	0,3039	0,255	0,2248	0,255	0,4237	0,2929	0,4001
P value	>0,1000	0,0284	>0,1000	>0,1000	>0,1000	0,0001	0,0419	0,0004
Passed n(	Yes	No	Yes	Yes	Yes	No	No	No
P value su	ns	*	ns	ns	ns	***	*	***
Number of	8	8	8	8	8	8	8	8

6. 2way ANOVA of Y-maze FORCED_Duration 30-60D intergroup

**Table Analyzed**	Y-maze FORCED_Duration 30-60D				
**Two-way RM ANOVA**	Matching: Stacked				
**Assume sphericity?**	Yes				
**Alpha**	0,05				
**Source of Variation**	% of total variation	P value	P value summary	Significant?	
**Time x Column Factor**	8,507	0,0067	**	Yes	
**Time**	25,63	<0,0001	****	Yes	
**Column Factor**	31,65	<0,0001	****	Yes	
**Subject**	18,30	0,3569	ns	No	
**ANOVA table**	SS	DF	MS	F (DFn, DFd)	P value
**Time x Column Factor**	2010	3	670,0	F (3, 28) = 4,990	P=0,0067
**Time**	6057	1	6057	F (1, 28) = 45,11	P<0,0001
**Column Factor**	7477	3	2492	F (3, 28) = 16,14	P<0,0001
**Subject**	4324	28	154,4	F (28, 28) = 1,150	P=0,3569
**Residual**	3759	28	134,3		
**Difference between row means**					
**Mean of 30**	35,47				
**Mean of 60**	16,02				
**Difference between means**	19,46				
**SE of difference**	2,897				
**95% CI of difference**	13,52 to 25,39				
**Data summary**					
**Number of columns (Column Factor)**	4				
**Number of rows (Time)**	2				
**Number of subjects (Subject)**	32				
**Number of missing values**	0				

	WT-sham 3	WT-Sham 6	WT-mTBI 3(	WT-mTBI 6(	APP-Sham :	APP-Sham (	APP-mTBI 3	APP-mTBI 6
Test for normal distribution								
D'Agostino & Pearson test								
K2	0,1594	0,1181	1,561	3,937	0,9235	4,325	0,4407	21,4
P value	0,9234	0,9427	0,4583	0,1397	0,6302	0,115	0,8022	<0,0001
Passed n(	Yes	Yes	Yes	Yes	Yes	Yes	Yes	No
P value su	ns	ns	ns	ns	ns	ns	ns	****
Anderson-Darling test								
A2*	0,1571	0,1605	0,3721	0,7254	0,3025	1,552	0,2461	1,848
P value	0,9208	0,9142	0,3252	0,0344	0,4943	0,0002	0,6518	<0,0001
Passed n(	Yes	Yes	Yes	No	Yes	No	Yes	No
P value su	ns	ns	ns	*	ns	***	ns	****
Shapiro-Wilk test								
W	0,979	0,9716	0,8925	0,7985	0,9324	0,6211	0,9428	0,5192
P value	0,9579	0,91	0,2468	0,0276	0,5381	0,0003	0,6387	<0,0001
Passed n(	Yes	Yes	Yes	No	Yes	No	Yes	No
P value su	ns	ns	ns	*	ns	***	ns	****
Kolmogorov-Smirnov test								
KS distan	0,1314	0,131	0,1885	0,2546	0,2132	0,44	0,154	0,3787
P value	>0,1000	>0,1000	>0,1000	>0,1000	>0,1000	<0,0001	>0,1000	0,0012
Passed n(	Yes	Yes	Yes	Yes	Yes	No	Yes	No
P value su	ns	ns	ns	ns	ns	****	ns	**
Number of '	8	8	8	8	8	8	8	8

7. 2way ANOVA of OF Travelling (primi 5 min) 30-60D intergroup

**Table Analyzed**	OF Travelling (primi 5 min) 30-60D				
**Two-way RM ANOVA**	Matching: Stacked				
**Assume sphericity?**	Yes				
**Alpha**	0,05				
**Source of Variation**	% of total variation	P value	P value summary	Significant?	
**Time x Column Factor**	3,126	0,1523	ns	No	
**Time**	3,600	0,0160	*	Yes	
**Column Factor**	44,22	<0,0001	****	Yes	
**Subject**	33,71	0,0208	*	Yes	
**ANOVA table**	SS	DF	MS	F (DFn, DFd)	P value
**Time x Column Factor**	1732997	3	577666	F (3, 28) = 1,901	P=0,1523
**Time**	1995788	1	1995788	F (1, 28) = 6,569	P=0,0160
**Column Factor**	24517259	3	8172420	F (3, 28) = 12,24	P<0,0001
**Subject**	18687491	28	667410	F (28, 28) = 2,197	P=0,0208
**Residual**	8506473	28	303803		
**Difference between row means**					
**Mean of 30**	1926				
**Mean of 60**	2279				
**Difference between means**	-353,2				
**SE of difference**	137,8				
**95% CI of difference**	-635,4 to -70,92				
**Data summary**					
**Number of columns (Column Factor)**	4				
**Number of rows (Time)**	2				
**Number of subjects (Subject)**	32				
**Number of missing values**	0				

	WT-sham 3	WT-Sham 6	WT-mTBI 3(	WT-mTBI 6(	APP-Sham :	APP-Sham (	APP-mTBI 3	APP-mTBI 6
Test for normal distribution								
D'Agostino & Pearson test								
K2	2,934	0,1069	0,4349	0,7351	0,4067	1,783	13,35	3,372
P value	0,2306	0,948	0,8046	0,6924	0,816	0,41	0,0013	0,1853
Passed n(	Yes	Yes	Yes	Yes	Yes	Yes	No	Yes
P value su	ns	ns	ns	ns	ns	ns	**	ns
Anderson-Darling test								
A2*	0,3399	0,2826	0,2311	0,2482	0,2303	0,2363	0,8635	0,5294
P value	0,3955	0,5347	0,7086	0,6438	0,7115	0,6887	0,0142	0,1186
Passed n(	Yes	Yes	Yes	Yes	Yes	Yes	No	Yes
P value su	ns	ns	ns	ns	ns	ns	*	ns
Shapiro-Wilk test								
W	0,9006	0,9359	0,9478	0,9528	0,9449	0,9406	0,7514	0,8816
P value	0,2923	0,5716	0,6895	0,7393	0,6599	0,6169	0,0085	0,1951
Passed n(	Yes	Yes	Yes	Yes	Yes	Yes	No	Yes
P value su	ns	ns	ns	ns	ns	ns	**	ns
Kolmogorov-Smirnov test								
KS distan	0,1636	0,1689	0,1579	0,1616	0,1452	0,1365	0,2641	0,2617
P value	>0,1000	>0,1000	>0,1000	>0,1000	>0,1000	>0,1000	>0,1000	>0,1000
Passed n(	Yes	Yes	Yes	Yes	Yes	Yes	Yes	Yes
P value su	ns	ns	ns	ns	ns	ns	ns	ns
Number of '	8	8	8	8	8	8	8	8

8. 2way ANOVA of OFT time in center % primi 10 min 30-60D intergroup

**Table Analyzed**	OFT time in center % primi 10 min 30-60D				
**Two-way RM ANOVA**	Matching: Stacked				
**Assume sphericity?**	Yes				
**Alpha**	0,05				
**Source of Variation**	% of total variation	P value	P value summary	Significant?	
**Time x Column Factor**	0,4184	0,9250	ns	No	
**Time**	0,002467	0,9585	ns	No	
**Column Factor**	40,98	<0,0001	****	Yes	
**Subject**	33,55	0,2220	ns	No	
**ANOVA table**	SS	DF	MS	F (DFn, DFd)	P value
**Time x Column Factor**	2,867	3	0,9555	F (3, 28) = 0,1559	P=0,9250
**Time**	0,01690	1	0,01690	F (1, 28) = 0,002758	P=0,9585
**Column Factor**	280,7	3	93,58	F (3, 28) = 11,40	P<0,0001
**Subject**	229,9	28	8,210	F (28, 28) = 1,340	P=0,2220
**Residual**	171,6	28	6,128		
**Difference between row means**					
**Mean of 30**	7,958				
**Mean of 60**	7,990				
**Difference between means**	-0,03250				
**SE of difference**	0,6189				
**95% CI of difference**	-1,300 to 1,235				
**Data summary**					
**Number of columns (Column Factor)**	4				
**Number of rows (Time)**	2				
**Number of subjects (Subject)**	32				
**Number of missing values**	0				

	WT-sham 3	WT-Sham 6	WT-mTBI 3(	WT-mTBI 6(	APP-Sham :	APP-Sham (	APP-mTBI 3	APP-mTBI 6
Test for normal distribution								
D'Agostino & Pearson test								
K2	1,697	0,5699	2,365	1,388	1,065	2,163	1,119	7,277
P value	0,428	0,7521	0,3065	0,4995	0,587	0,339	0,5715	0,0263
Passed n(	Yes	Yes	Yes	Yes	Yes	Yes	Yes	No
P value su	ns	ns	ns	ns	ns	ns	ns	*
Anderson-Darling test								
A2*	0,3792	0,229	0,3674	0,2693	0,2245	0,389	0,3084	0,9561
P value	0,3113	0,7163	0,3347	0,5714	0,7333	0,2931	0,4775	0,0079
Passed n(	Yes	Yes	Yes	Yes	Yes	Yes	Yes	No
P value su	ns	ns	ns	ns	ns	ns	ns	**
Shapiro-Wilk test								
W	0,8997	0,9469	0,9074	0,9279	0,943	0,9238	0,9545	0,7445
P value	0,2873	0,6804	0,3363	0,4971	0,6412	0,4618	0,7563	0,0071
Passed n(	Yes	Yes	Yes	Yes	Yes	Yes	Yes	No
P value su	ns	ns	ns	ns	ns	ns	ns	**
Kolmogorov-Smirnov test								
KS distan	0,1956	0,1341	0,224	0,173	0,1687	0,2182	0,1955	0,3499
P value	>0,1000	>0,1000	>0,1000	>0,1000	>0,1000	>0,1000	>0,1000	0,0046
Passed n(	Yes	Yes	Yes	Yes	Yes	Yes	Yes	No
P value su	ns	ns	ns	ns	ns	ns	ns	**
Number of	8	8	8	8	8	8	8	8

9 2way ANOVA of NOR 30-60D 1

**Within each row, compare columns (simple effects within rows)**								
**Number of families**	2							
**Number of comparisons per family**	3							
**Alpha**	0,05							
**Dunnett's multiple comparisons test**	Mean Diff,	95,00% CI of diff,	Below threshold?	Summary				
**30**								
**WT-sham vs. WT-mTBI**	0,2100	-0,01609 to 0,4361	No	ns				
**WT-sham vs. APP-sham**	0,1082	-0,1179 to 0,3343	No	ns				
**WT-sham vs. APP-mTBI**	0,1132	-0,1129 to 0,3393	No	ns				
**60**								
**WT-sham vs. WT-mTBI**	0,5710	0,3448 to 0,7971	Yes	****				
**WT-sham vs. APP-sham**	0,1875	-0,03861 to 0,4136	No	ns				
**WT-sham vs. APP-mTBI**	0,2401	0,01400 to 0,4662	Yes	*				
**Test details**	Mean 1	Mean 2	Mean Diff,	SE of diff,	N1	N2	q	DF
**30**								
**WT-sham vs. WT-mTBI**	0,3794	0,1694	0,2100	0,09365	8	8	2,243	56,00
**WT-sham vs. APP-sham**	0,3794	0,2713	0,1082	0,09365	8	8	1,155	56,00
**WT-sham vs. APP-mTBI**	0,3794	0,2663	0,1132	0,09365	8	8	1,209	56,00
**60**								
**WT-sham vs. WT-mTBI**	0,3963	-0,1747	0,5710	0,09365	8	8	6,097	56,00
**WT-sham vs. APP-sham**	0,3963	0,2088	0,1875	0,09365	8	8	2,002	56,00
**WT-sham vs. APP-mTBI**	0,3963	0,1561	0,2401	0,09365	8	8	2,564	56,00

	WT-sham 3	WT-Sham 6	WT-mTBI 3(	WT-mTBI 6(	APP-Sham :	APP-Sham (	APP-mTBI 3	APP-mTBI 6
Test for normal distribution								
D'Agostino & Pearson test								
K2	3,883	4,519	2,675	0,6672	7,396	3,235	0,05592	3,365
P value	0,1435	0,1044	0,2625	0,7163	0,0248	0,1984	0,9724	0,1859
Passed n(	Yes	Yes	Yes	Yes	No	Yes	Yes	Yes
P value su	ns	ns	ns	ns	*	ns	ns	ns
Anderson-Darling test								
A2*	0,449	0,709	0,4098	0,2534	0,5313	0,3173	0,2808	0,3409
P value	0,2007	0,0382	0,2573	0,6248	0,117	0,4529	0,5391	0,3932
Passed n(	Yes	No	Yes	Yes	Yes	Yes	Yes	Yes
P value su	ns	*	ns	ns	ns	ns	ns	ns
Shapiro-Wilk test								
W	0,8856	0,8181	0,8966	0,9516	0,8657	0,9199	0,9471	0,9151
P value	0,2127	0,0446	0,2694	0,727	0,1367	0,429	0,6817	0,3914
Passed n(	Yes	No	Yes	Yes	Yes	Yes	Yes	Yes
P value su	ns	*	ns	ns	ns	ns	ns	ns
Kolmogorov-Smirnov test								
KS distan	0,2393	0,2722	0,2079	0,2159	0,24	0,1618	0,1704	0,2065
P value	>0,1000	0,0828	>0,1000	>0,1000	>0,1000	>0,1000	>0,1000	>0,1000
Passed n(	Yes	Yes	Yes	Yes	Yes	Yes	Yes	Yes
P value su	ns	ns	ns	ns	ns	ns	ns	ns
Number of '	8	8	8	8	8	8	8	8

10 2way ANOVA of NOR 30-60D 24h intergroup

**Within each row, compare columns (simple effects within rows)**								
**Number of families**	2							
**Number of comparisons per family**	3							
**Alpha**	0,05							
**Dunnett's multiple comparisons test**	Mean Diff,	95,00% CI of diff,	Below threshold?	Summary				
**30**								
**WT-sham vs. WT-mTBI**	0,1011	-0,1928 to 0,3950	No	ns				
**WT-sham vs. APP-sham**	-0,02431	-0,3182 to 0,2696	No	ns				
**WT-sham vs. APP-mTBI**	0,2557	-0,03822 to 0,5496	No	ns				
**60**								
**WT-sham vs. WT-mTBI**	0,3446	0,05066 to 0,6385	Yes	*				
**WT-sham vs. APP-sham**	0,08928	-0,2046 to 0,3832	No	ns				
**WT-sham vs. APP-mTBI**	0,09365	-0,2003 to 0,3876	No	ns				
**Test details**	Mean 1	Mean 2	Mean Diff,	SE of diff,	N1	N2	q	DF
**30**								
**WT-sham vs. WT-mTBI**	0,1594	0,05838	0,1011	0,1217	8	8	0,8303	56,00
**WT-sham vs. APP-sham**	0,1594	0,1838	-0,02431	0,1217	8	8	0,1997	56,00
**WT-sham vs. APP-mTBI**	0,1594	-0,09625	0,2557	0,1217	8	8	2,101	56,00
**60**								
**WT-sham vs. WT-mTBI**	0,2149	-0,1297	0,3446	0,1217	8	8	2,831	56,00
**WT-sham vs. APP-sham**	0,2149	0,1256	0,08928	0,1217	8	8	0,7335	56,00
**WT-sham vs. APP-mTBI**	0,2149	0,1213	0,09365	0,1217	8	8	0,7694	56,00

	WT-sham 3	WT-Sham 6	WT-mTBI 3(	WT-mTBI 6(	APP-Sham :	APP-Sham (	APP-mTBI 3	APP-mTBI 6
Test for normal distribution								
D'Agostino & Pearson test								
K2	1,05	1,466	0,403	0,8877	0,4505	3,345	3,763	0,2435
P value	0,5915	0,4805	0,8175	0,6416	0,7983	0,1878	0,1523	0,8854
Passed n(	Yes	Yes	Yes	Yes	Yes	Yes	Yes	Yes
P value su	ns	ns	ns	ns	ns	ns	ns	ns
Anderson-Darling test								
A2*	0,2164	0,2732	0,236	0,3762	0,1464	0,562	0,537	0,1336
P value	0,7631	0,5598	0,6897	0,317	0,941	0,0982	0,1153	0,962
Passed n(	Yes	Yes	Yes	Yes	Yes	Yes	Yes	Yes
P value su	ns	ns	ns	ns	ns	ns	ns	ns
Shapiro-Wilk test								
W	0,9471	0,9245	0,965	0,8935	0,9753	0,873	0,878	0,9813
P value	0,6819	0,4673	0,8561	0,2524	0,9358	0,1611	0,1804	0,969
Passed n(	Yes	Yes	Yes	Yes	Yes	Yes	Yes	Yes
P value su	ns	ns	ns	ns	ns	ns	ns	ns
Kolmogorov-Smirnov test								
KS distan	0,1428	0,1542	0,1764	0,2053	0,1216	0,2496	0,2726	0,1151
P value	>0,1000	>0,1000	>0,1000	>0,1000	>0,1000	>0,1000	0,0818	>0,1000
Passed n(	Yes	Yes	Yes	Yes	Yes	Yes	Yes	Yes
P value su	ns	ns	ns	ns	ns	ns	ns	ns
Number of v	8	8	8	8	8	8	8	8

2way ANOVA of Ab1-42 cortex

Table Analyzed	Ab1-42 cortex				
Two-way ANOVA	Ordinary				
Alpha	0,05				
Source of Variation	% of total variation	P value	P value summary	Significant?	
Interaction	47,55	0,0015	**	Yes	
Injury	2,382	0,3213	ns	No	
Genotype	33,02	0,0043	**	Yes	
ANOVA table	SS	DF	MS	F (DFn, DFd)	P value
Interaction	0,1323	1	0,1323	F (1, 8) = 22.31	P=0.0015
Injury	0,006627	1	0,006627	F (1, 8) = 1.118	P=0.3213
Genotype	0,09188	1	0,09188	F (1, 8) = 15.49	P=0.0043
Residual	0,04744	8	0,005930		
Difference between column means					
Mean of WT	0,4658				
Mean of APP-SWE	0,6408				
Difference between means	-0,1750				
SE of difference	0,04446				
95% CI of difference	-0.2775 to -0.07247				
Difference between row means					
Mean of Sham	0,5298				
Mean of mTBI	0,5768				
Difference between means	-0,04700				
SE of difference	0,04446				
95% CI of difference	-0.1495 to 0.05553				
Interaction CI					
Mean diff, A1 - B1	0,03500				
Mean diff, A2 - B2	-0,3850				
(A1 -B1) - (A2 - B2)	0,4200				
95% CI of difference	0.2149 to 0.6251				
(B1 - A1) - (B2 - A2)	-0,4200				
95% CI of difference	-0.6251 to -0.2149				
Normality of Residuals					
Test name	Statistics	P value	Passed normality test (alpha=0.05)?	P value summary	
D'Agostino-Pearson omnibus (K2)	3,171	0,2049	Yes	ns	
Anderson-Darling (A2*)	0,5666	0,1123	Yes	ns	
Shapiro-Wilk (W)	0,9184	0,2730	Yes	ns	
Kolmogorov-Smirnov (distance)	0,1975	0,1000	Yes	ns	
Data summary					

**Number of columns (Genotype)**	2				
**Number of rows (Injury)**	2				
**Number of values**	12				

2way ANOVA of Ab1-42 hipp

Table Analyzed	Ab1-42 hipp				
Two-way ANOVA	Ordinary				
Alpha	0,05				
Source of Variation	% of total variation	P value	P value summary	Significant?	
Interaction	18,52	0,0587	ns	No	
Injury	3,936	0,3395	ns	No	
Genotype	47,02	0,0080	**	Yes	
ANOVA table	SS	DF	MS	F (DFn, DFd)	P value
Interaction	0,01031	1	0,01031	F (1, 8) = 4.853	P=0.0587
Injury	0,002192	1	0,002192	F (1, 8) = 1.032	P=0.3395
Genotype	0,02619	1	0,02619	F (1, 8) = 12.32	P=0.0080
Residual	0,01700	8	0,002125		
Difference between column means					
Mean of WT	0,1922				
Mean of APP-SWE	0,09873				
Difference between means	0,09343				
SE of difference	0,02662				
95% CI of difference	0.03205 to 0.1548				
Difference between row means					
Mean of Sham	0,1319				
Mean of mTBI	0,1590				
Difference between means	-0,02703				
SE of difference	0,02662				
95% CI of difference	-0.08841 to 0.03435				
Interaction CI					
Mean diff, A1 - B1	0,03480				
Mean diff, A2 - B2	0,1521				
(A1 -B1) - (A2 - B2)	-0,1173				
95% CI of difference	-0.2400 to 0.005492				
(B1 - A1) - (B2 - A2)	0,1173				
95% CI of difference	-0.005492 to 0.2400				
Normality of Residuals					
Test name	Statistics	P value	Passed normality test (alpha=0.05)?	P value summary	
D'Agostino-Pearson omnibus (K2)	0,3082	0,8572	Yes	ns	
Anderson-Darling (A2*)	0,2616	0,6380	Yes	ns	
Shapiro-Wilk (W)	0,9561	0,7265	Yes	ns	
Kolmogorov-Smirnov (distance)	0,1464	0,1000	Yes	ns	
Data summary					

**Number of columns (Genotype)**	2				
**Number of rows (Injury)**	2				
**Number of values**	12				

2way ANOVA of Ab1-40 cortex

Table Analyzed	Ab1-40 cortex				
Two-way ANOVA	Ordinary				
Alpha	0,05				
Source of Variation	% of total variation	P value	P value summary	Significant?	
Interaction	24,85	0,0661	ns	No	
Injury	16,97	0,1168	ns	No	
Genotype	14,25	0,1458	ns	No	
ANOVA table	SS	DF	MS	F (DFn, DFd)	P value
Interaction	0,02162	1	0,02162	F (1, 8) = 4.526	P=0.0661
Injury	0,01477	1	0,01477	F (1, 8) = 3.091	P=0.1168
Genotype	0,01240	1	0,01240	F (1, 8) = 2.596	P=0.1458
Residual	0,03822	8	0,004778		
Difference between column means					
Mean of WT	0,1435				
Mean of APP-SWE	0,07917				
Difference between means	0,06430				
SE of difference	0,03991				
95% CI of difference	-0.02773 to 0.1563				
Difference between row means					
Mean of Sham	0,1464				
Mean of mTBI	0,07623				
Difference between means	0,07017				
SE of difference	0,03991				
95% CI of difference	-0.02186 to 0.1622				
Interaction CI					
Mean diff, A1 - B1	0,1492				
Mean diff, A2 - B2	-0,02060				
(A1 -B1) - (A2 - B2)	0,1698				
95% CI of difference	-0.01426 to 0.3539				
(B1 - A1) - (B2 - A2)	-0,1698				
95% CI of difference	-0.3539 to 0.01426				
Normality of Residuals					
Test name	Statistics	P value	Passed normality test (alpha=0.05)?	P value summary	
D'Agostino-Pearson omnibus (K2)	7,321	0,0257	No	*	
Anderson-Darling (A2*)	0,9416	0,0113	No	*	
Shapiro-Wilk (W)	0,8396	0,0274	No	*	
Kolmogorov-Smirnov (distance)	0,2873	0,0071	No	**	
Data summary					

**Number of columns (Genotype)**	2				
**Number of rows (Injury)**	2				
**Number of values**	12				

2way ANOVA of Ab1-40 hipp

Table Analyzed	Ab1-40 hipp				
Two-way ANOVA	Ordinary				
Alpha	0,05				
Source of Variation	% of total variation	P value	P value summary	Significant?	
Interaction	2,414	0,6086	ns	No	
Injury	13,08	0,2499	ns	No	
Genotype	16,50	0,2011	ns	No	
ANOVA table	SS	DF	MS	F (DFn, DFd)	P value
Interaction	0,0005575	1	0,0005575	F (1, 8) = 0.2840	P=0.6086
Injury	0,003021	1	0,003021	F (1, 8) = 1.539	P=0.2499
Genotype	0,003809	1	0,003809	F (1, 8) = 1.940	P=0.2011
Residual	0,01571	8	0,001963		
Difference between column means					
Mean of WT	0,06405				
Mean of APP-SWE	0,02842				
Difference between means	0,03563				
SE of difference	0,02558				
95% CI of difference	-0.02336 to 0.09462				
Difference between row means					
Mean of Sham	0,06210				
Mean of mTBI	0,03037				
Difference between means	0,03173				
SE of difference	0,02558				
95% CI of difference	-0.02726 to 0.09072				
Interaction CI					
Mean diff, A1 - B1	0,04927				
Mean diff, A2 - B2	0,02200				
(A1 -B1) - (A2 - B2)	0,02726				
95% CI of difference	-0.09072 to 0.1452				
(B1 - A1) - (B2 - A2)	-0,02726				
95% CI of difference	-0.1452 to 0.09072				
Normality of Residuals					
Test name	Statistics	P value	Passed normality test (alpha=0.05)?	P value summary	
D'Agostino-Pearson omnibus (K2)	5,442	0,0658	Yes	ns	
Anderson-Darling (A2*)	0,3788	0,3464	Yes	ns	
Shapiro-Wilk (W)	0,9146	0,2446	Yes	ns	
Kolmogorov-Smirnov (distance)	0,1564	0,1000	Yes	ns	
Data summary					

**Number of columns (Genotype)**	2				
**Number of rows (Injury)**	2				
**Number of values**	12				

2way ANOVA of BACE1 cortex

Table Analyzed	BACE1 cortex				
Two-way ANOVA	Ordinary				
Alpha	0,05				
Source of Variation	% of total variation	P value	P value summary	Significant?	
Interaction	16,15	0,0462	*	Yes	
Injury	10,44	0,1005	ns	No	
Genotype	13,06	0,0694	ns	No	
ANOVA table	SS (Type III)	DF	MS	F (DFn, DFd)	P value
Interaction	1,513	1	1,513	F (1, 14) = 4.783	P=0.0462
Injury	0,9778	1	0,9778	F (1, 14) = 3.092	P=0.1005
Genotype	1,222	1	1,222	F (1, 14) = 3.866	P=0.0694
Residual	4,427	14	0,3162		
Difference between column means					
Predicted (LS) mean of WT	0,9398				
Predicted (LS) mean of APP-SWE	1,493				
Difference between predicted means	-0,5528				
SE of difference	0,2812				
95% CI of difference	-1.156 to 0.05022				
Difference between row means					
Predicted (LS) mean of Sham	0,9689				
Predicted (LS) mean of mTBI	1,463				
Difference between predicted means	-0,4944				
SE of difference	0,2812				
95% CI of difference	-1.097 to 0.1086				
Interaction CI					
Mean diff, A1 - B1	0,06211				
Mean diff, A2 - B2	-1,168				
(A1 -B1) - (A2 - B2)	1,230				
95% CI of difference	0.02378 to 2.436				
(B1 - A1) - (B2 - A2)	-1,230				
95% CI of difference	-2.436 to -0.02378				
Normality of Residuals					
Test name	Statistics	P value	Passed normality test (alpha=0.05)?	P value summary	
D'Agostino-Pearson omnibus (K2)	3,940	0,1394	Yes	ns	
Anderson-Darling (A2*)	1,272	0,0019	No	**	
Shapiro-Wilk (W)	0,8560	0,0106	No	*	
Kolmogorov-Smirnov (distance)	0,2341	0,0102	No	*	
Data summary					
Number of columns (Genotype)	2				
Number of rows (Injury)	2				

**Number of values**	18				

2way ANOVA of BACE1 hipp

Table Analyzed	BACE1 hipp				
Two-way ANOVA	Ordinary				
Alpha	0,05				
Source of Variation	% of total variation	P value	P value summary	Significant?	
Interaction	2,128	0,4547	ns	No	
Injury	9,010	0,1310	ns	No	
Genotype	2,749	0,3963	ns	No	
ANOVA table	SS (Type III)	DF	MS	F (DFn, DFd)	P value
Interaction	0,08917	1	0,08917	F (1, 24) = 0.5775	P=0.4547
Injury	0,3775	1	0,3775	F (1, 24) = 2.445	P=0.1310
Genotype	0,1152	1	0,1152	F (1, 24) = 0.7459	P=0.3963
Residual	3,706	24	0,1544		
Difference between column means					
Predicted (LS) mean of WT	0,8088				
Predicted (LS) mean of APP-SWE	0,6666				
Difference between predicted means	0,1422				
SE of difference	0,1646				
95% CI of difference	-0.1976 to 0.4819				
Difference between row means					
Predicted (LS) mean of Sham	0,8664				
Predicted (LS) mean of mTBI	0,6090				
Difference between predicted means	0,2574				
SE of difference	0,1646				
95% CI of difference	-0.08235 to 0.5971				
Interaction CI					
Mean diff, A1 - B1	0,2673				
Mean diff, A2 - B2	0,01707				
(A1 -B1) - (A2 - B2)	0,2502				
95% CI of difference	-0.4293 to 0.9297				
(B1 - A1) - (B2 - A2)	-0,2502				
95% CI of difference	-0.9297 to 0.4293				
Normality of Residuals					
Test name	Statistics	P value	Passed normality test (alpha=0.05)?	P value summary	
D'Agostino-Pearson omnibus (K2)	3,096	0,2126	Yes	ns	
Anderson-Darling (A2*)	0,5180	0,1726	Yes	ns	
Shapiro-Wilk (W)	0,9512	0,2120	Yes	ns	
Kolmogorov-Smirnov (distance)	0,1411	0,1000	Yes	ns	
Data summary					
Number of columns (Genotype)	2				
Number of rows (Injury)	2				

**Number of values**	28				

2way ANOVA of BACE2 cortex

Table Analyzed	BACE2 cortex				
Two-way ANOVA	Ordinary				
Alpha	0,05				
Source of Variation	% of total variation	P value	P value summary	Significant?	
Interaction	2,699	0,4603	ns	No	
Injury	11,88	0,1319	ns	No	
Genotype	1,808	0,5445	ns	No	
ANOVA table	SS (Type III)	DF	MS	F (DFn, DFd)	P value
Interaction	0,2239	1	0,2239	F (1, 16) = 0.5725	P=0.4603
Injury	0,9856	1	0,9856	F (1, 16) = 2.521	P=0.1319
Genotype	0,1500	1	0,1500	F (1, 16) = 0.3835	P=0.5445
Residual	6,257	16	0,3910		
Difference between column means					
Predicted (LS) mean of WT	1,127				
Predicted (LS) mean of APP-SWE	1,316				
Difference between predicted means	-0,1890				
SE of difference	0,3051				
95% CI of difference	-0.8358 to 0.4579				
Difference between row means					
Predicted (LS) mean of Sham	0,9790				
Predicted (LS) mean of mTBI	1,463				
Difference between predicted means	-0,4844				
SE of difference	0,3051				
95% CI of difference	-1.131 to 0.1624				
Interaction CI					
Mean diff, A1 - B1	0,04193				
Mean diff, A2 - B2	-0,4198				
(A1 -B1) - (A2 - B2)	0,4618				
95% CI of difference	-0.8319 to 1.755				
(B1 - A1) - (B2 - A2)	-0,4618				
95% CI of difference	-1.755 to 0.8319				
Normality of Residuals					
Test name	Statistics	P value	Passed normality test (alpha=0.05)?	P value summary	
D'Agostino-Pearson omnibus (K2)	0,7414	0,6903	Yes	ns	
Anderson-Darling (A2*)	1,333	0,0014	No	**	
Shapiro-Wilk (W)	0,8651	0,0096	No	**	
Kolmogorov-Smirnov (distance)	0,2243	0,0096	No	**	
Data summary					
Number of columns (Genotype)	2				
Number of rows (Injury)	2				

**Number of values**	20				

2way ANOVA of BACE2 hipp

Table Analyzed	BACE2 hipp				
Two-way ANOVA	Ordinary				
Alpha	0,05				
Source of Variation	% of total variation	P value	P value summary	Significant?	
Interaction	0,2423	0,7556	ns	No	
Injury	7,805	0,0859	ns	No	
Genotype	27,70	0,0024	**	Yes	
ANOVA table	SS (Type III)	DF	MS	F (DFn, DFd)	P value
Interaction	0,01819	1	0,01819	F (1, 26) = 0.09898	P=0.7556
Injury	0,5859	1	0,5859	F (1, 26) = 3.188	P=0.0859
Genotype	2,079	1	2,079	F (1, 26) = 11.31	P=0.0024
Residual	4,778	26	0,1838		
Difference between column means					
Predicted (LS) mean of WT	0,8142				
Predicted (LS) mean of APP-SWE	1,409				
Difference between predicted means	-0,5953				
SE of difference	0,1770				
95% CI of difference	-0.9591 to -0.2315				
Difference between row means					
Predicted (LS) mean of Sham	1,270				
Predicted (LS) mean of mTBI	0,9538				
Difference between predicted means	0,3160				
SE of difference	0,1770				
95% CI of difference	-0.04780 to 0.6798				
Interaction CI					
Mean diff, A1 - B1	-0,5397				
Mean diff, A2 - B2	-0,6510				
(A1 -B1) - (A2 - B2)	0,1114				
95% CI of difference	-0.6162 to 0.8390				
(B1 - A1) - (B2 - A2)	-0,1114				
95% CI of difference	-0.8390 to 0.6162				
Normality of Residuals					
Test name	Statistics	P value	Passed normality test (alpha=0.05)?	P value summary	
D'Agostino-Pearson omnibus (K2)	7,696	0,0213	No	*	
Anderson-Darling (A2*)	0,7163	0,0551	Yes	ns	
Shapiro-Wilk (W)	0,9356	0,0691	Yes	ns	
Kolmogorov-Smirnov (distance)	0,1419	0,1000	Yes	ns	
Data summary					
Number of columns (Genotype)	2				
Number of rows (Injury)	2				
Number of values	30				

2way ANOVA of IFN-g plasma

Table Analyzed	IFN-g plasma				
Two-way ANOVA	Ordinary				
Alpha	0,05				
Source of Variation	% of total variation	P value	P value summary	Significant?	
Interaction	0,6451	0,4686	ns	No	
Injury	10,40	0,0118	*	Yes	
Genotype	74,26	<0.0001	****	Yes	
ANOVA table	SS (Type III)	DF	MS	F (DFn, DFd)	P value
Interaction	1,842E-05	1	1,842E-05	F (1, 11) = 0.5635	P=0.4686
Injury	0,0002968	1	0,0002968	F (1, 11) = 9.081	P=0.0118
Genotype	0,002120	1	0,002120	F (1, 11) = 64.86	P<0.0001
Residual	0,0003595	11	3,268E-05		
Difference between column means					
Predicted (LS) mean of WT	0,02422				
Predicted (LS) mean of APP-SWE	0,04854				
Difference between predicted means	-0,02433				
SE of difference	0,003021				
95% CI of difference	-0.03098 to -0.01768				
Difference between row means					
Predicted (LS) mean of Sham	0,03183				
Predicted (LS) mean of mTBI	0,04093				
Difference between predicted means	-0,009103				
SE of difference	0,003021				
95% CI of difference	-0.01575 to -0.002454				
Interaction CI					
Mean diff, A1 - B1	-0,02660				
Mean diff, A2 - B2	-0,02206				
(A1 -B1) - (A2 - B2)	-0,004535				
95% CI of difference	-0.01783 to 0.008762				
(B1 - A1) - (B2 - A2)	0,004535				
95% CI of difference	-0.008762 to 0.01783				
Normality of Residuals					
Test name	Statistics	P value	Passed normality test (alpha=0.05)?	P value summary	
D'Agostino-Pearson omnibus (K2)	0,1052	0,9488	Yes	ns	
Anderson-Darling (A2*)	0,1947	0,8697	Yes	ns	
Shapiro-Wilk (W)	0,9694	0,8491	Yes	ns	
Kolmogorov-Smirnov (distance)	0,1098	0,1000	Yes	ns	
Data summary					
Number of columns (Genotype)	2				
Number of rows (Injury)	2				
Number of values	15				

2way ANOVA of IL-17A plasma

Table Analyzed	IL-17A plasma				
Two-way ANOVA	Ordinary				
Alpha	0,05				
Source of Variation	% of total variation	P value	P value summary	Significant?	
Interaction	17,26	0,0050	**	Yes	
Injury	50,06	0,0001	***	Yes	
Genotype	24,08	0,0018	**	Yes	
ANOVA table	SS (Type III)	DF	MS	F (DFn, DFd)	P value
Interaction	0,09225	1	0,09225	F (1, 9) = 13.59	P=0.0050
Injury	0,2675	1	0,2675	F (1, 9) = 39.42	P=0.0001
Genotype	0,1287	1	0,1287	F (1, 9) = 18.96	P=0.0018
Residual	0,06107	9	0,006786		
Difference between column means					
Predicted (LS) mean of WT	0,7767				
Predicted (LS) mean of APP-SWE	0,9772				
Difference between predicted means	-0,2005				
SE of difference	0,04605				
95% CI of difference	-0.3047 to -0.09637				
Difference between row means					
Predicted (LS) mean of Sham	0,7324				
Predicted (LS) mean of mTBI	1,022				
Difference between predicted means	-0,2891				
SE of difference	0,04605				
95% CI of difference	-0.3933 to -0.1850				
Interaction CI					
Mean diff, A1 - B1	-0,03075				
Mean diff, A2 - B2	-0,3703				
(A1 -B1) - (A2 - B2)	0,3396				
95% CI of difference	0.1312 to 0.5479				
(B1 - A1) - (B2 - A2)	-0,3396				
95% CI of difference	-0.5479 to -0.1312				
Normality of Residuals					
Test name	Statistics	P value	Passed normality test (alpha=0.05)?	P value summary	
D'Agostino-Pearson omnibus (K2)	0,7892	0,6740	Yes	ns	
Anderson-Darling (A2*)	0,2879	0,5598	Yes	ns	
Shapiro-Wilk (W)	0,9582	0,7253	Yes	ns	
Kolmogorov-Smirnov (distance)	0,1595	0,1000	Yes	ns	
Data summary					
Number of columns (Genotype)	2				
Number of rows (Injury)	2				

**Number of values**	13				

2way ANOVA of IL-1beta plasma

Table Analyzed	IL-1beta plasma				
Two-way ANOVA	Ordinary				
Alpha	0,05				
Source of Variation	% of total variation	P value	P value summary	Significant?	
Interaction	0,7104	0,3525	ns	No	
Injury	46,99	<0.0001	****	Yes	
Genotype	48,81	<0.0001	****	Yes	
ANOVA table	SS (Type III)	DF	MS	F (DFn, DFd)	P value
Interaction	0,0003415	1	0,0003415	F (1, 11) = 0.9424	P=0.3525
Injury	0,02258	1	0,02258	F (1, 11) = 62.33	P<0.0001
Genotype	0,02346	1	0,02346	F (1, 11) = 64.75	P<0.0001
Residual	0,003986	11	0,0003623		
Difference between column means					
Predicted (LS) mean of WT	0,06075				
Predicted (LS) mean of APP-SWE	0,1417				
Difference between predicted means	-0,08093				
SE of difference	0,01006				
95% CI of difference	-0.1031 to -0.05879				
Difference between row means					
Predicted (LS) mean of Sham	0,06151				
Predicted (LS) mean of mTBI	0,1409				
Difference between predicted means	-0,07940				
SE of difference	0,01006				
95% CI of difference	-0.1015 to -0.05727				
Interaction CI					
Mean diff, A1 - B1	-0,09069				
Mean diff, A2 - B2	-0,07117				
(A1 -B1) - (A2 - B2)	-0,01953				
95% CI of difference	-0.06380 to 0.02475				
(B1 - A1) - (B2 - A2)	0,01953				
95% CI of difference	-0.02475 to 0.06380				
Normality of Residuals					
Test name	Statistics	P value	Passed normality test (alpha=0.05)?	P value summary	
D'Agostino-Pearson omnibus (K2)	1,374	0,5030	Yes	ns	
Anderson-Darling (A2*)	0,2769	0,6015	Yes	ns	
Shapiro-Wilk (W)	0,9733	0,9039	Yes	ns	
Kolmogorov-Smirnov (distance)	0,1324	0,1000	Yes	ns	
Data summary					
Number of columns (Genotype)	2				
Number of rows (Injury)	2				
Number of values	15				

2way ANOVA of TNF-a plasma

Table Analyzed	TNF-a plasma				
Two-way ANOVA	Ordinary				
Alpha	0,05				
Source of Variation	% of total variation	P value	P value summary	Significant?	
Interaction	8,830	0,1336	ns	No	
Injury	50,42	0,0023	**	Yes	
Genotype	6,555	0,1909	ns	No	
ANOVA table	SS (Type III)	DF	MS	F (DFn, DFd)	P value
Interaction	0,008536	1	0,008536	F (1, 12) = 2.589	P=0.1336
Injury	0,04874	1	0,04874	F (1, 12) = 14.78	P=0.0023
Genotype	0,006336	1	0,006336	F (1, 12) = 1.922	P=0.1909
Residual	0,03956	12	0,003297		
Difference between column means					
Predicted (LS) mean of WT	0,5189				
Predicted (LS) mean of APP-SWE	0,5594				
Difference between predicted means	-0,04046				
SE of difference	0,02918				
95% CI of difference	-0.1040 to 0.02313				
Difference between row means					
Predicted (LS) mean of Sham	0,4830				
Predicted (LS) mean of mTBI	0,5953				
Difference between predicted means	-0,1122				
SE of difference	0,02918				
95% CI of difference	-0.1758 to -0.04862				
Interaction CI					
Mean diff, A1 - B1	-0,08742				
Mean diff, A2 - B2	0,006500				
(A1 -B1) - (A2 - B2)	-0,09392				
95% CI of difference	-0.2211 to 0.03326				
(B1 - A1) - (B2 - A2)	0,09392				
95% CI of difference	-0.03326 to 0.2211				
Normality of Residuals					
Test name	Statistics	P value	Passed normality test (alpha=0.05)?	P value summary	
D'Agostino-Pearson omnibus (K2)	4,182	0,1236	Yes	ns	
Anderson-Darling (A2*)	0,6144	0,0906	Yes	ns	
Shapiro-Wilk (W)	0,9284	0,2298	Yes	ns	
Kolmogorov-Smirnov (distance)	0,2001	0,0864	Yes	ns	
Data summary					
Number of columns (Genotype)	2				
Number of rows (Injury)	2				

**Number of values**	16				

2way ANOVA of IL22 plasma

Table Analyzed	IL22 plasma				
Two-way ANOVA	Ordinary				
Alpha	0,05				
Source of Variation	% of total variation	P value	P value summary	Significant?	
Interaction	26,37	0,0717	ns	No	
Injury	0,8996	0,7199	ns	No	
Genotype	0,9514	0,7123	ns	No	
ANOVA table	SS (Type III)	DF	MS	F (DFn, DFd)	P value
Interaction	0,05500	1	0,05500	F (1, 11) = 3.970	P=0.0717
Injury	0,001876	1	0,001876	F (1, 11) = 0.1354	P=0.7199
Genotype	0,001984	1	0,001984	F (1, 11) = 0.1432	P=0.7123
Residual	0,1524	11	0,01385		
Difference between column means					
Predicted (LS) mean of WT	0,1776				
Predicted (LS) mean of APP-SWE	0,2011				
Difference between predicted means	-0,02354				
SE of difference	0,06219				
95% CI of difference	-0.1604 to 0.1133				
Difference between row means					
Predicted (LS) mean of Sham	0,1779				
Predicted (LS) mean of mTBI	0,2008				
Difference between predicted means	-0,02289				
SE of difference	0,06219				
95% CI of difference	-0.1598 to 0.1140				
Interaction CI					
Mean diff, A1 - B1	-0,1475				
Mean diff, A2 - B2	0,1004				
(A1 -B1) - (A2 - B2)	-0,2478				
95% CI of difference	-0.5216 to 0.02592				
(B1 - A1) - (B2 - A2)	0,2478				
95% CI of difference	-0.02592 to 0.5216				
Normality of Residuals					
Test name	Statistics	P value	Passed normality test (alpha=0.05)?	P value summary	
D'Agostino-Pearson omnibus (K2)	1,408	0,4945	Yes	ns	
Anderson-Darling (A2*)	0,2220	0,7911	Yes	ns	
Shapiro-Wilk (W)	0,9642	0,7643	Yes	ns	
Kolmogorov-Smirnov (distance)	0,1292	0,1000	Yes	ns	
Data summary					
Number of columns (Genotype)	2				
Number of rows (Injury)	2				

**Number of values**	15				

2way ANOVA of IL6 plasma

Table Analyzed	IL6 plasma				
Two-way ANOVA	Ordinary				
Alpha	0,05				
Source of Variation	% of total variation	P value	P value summary	Significant?	
Interaction	37,35	0,0449	*	Yes	
Injury	0,2756	0,8460	ns	No	
Genotype	1,308	0,6735	ns	No	
ANOVA table	SS (Type III)	DF	MS	F (DFn, DFd)	P value
Interaction	0,0003779	1	0,0003779	F (1, 9) = 5.416	P=0.0449
Injury	2,788E-06	1	2,788E-06	F (1, 9) = 0.03996	P=0.8460
Genotype	1,323E-05	1	1,323E-05	F (1, 9) = 0.1896	P=0.6735
Residual	0,0006279	9	6,977E-05		
Difference between column means					
Predicted (LS) mean of WT	0,01843				
Predicted (LS) mean of APP-SWE	0,02047				
Difference between predicted means	-0,002033				
SE of difference	0,004669				
95% CI of difference	-0.01260 to 0.008529				
Difference between row means					
Predicted (LS) mean of Sham	0,01898				
Predicted (LS) mean of mTBI	0,01992				
Difference between predicted means	-0,0009333				
SE of difference	0,004669				
95% CI of difference	-0.01150 to 0.009629				
Interaction CI					
Mean diff, A1 - B1	-0,01290				
Mean diff, A2 - B2	0,008833				
(A1 -B1) - (A2 - B2)	-0,02173				
95% CI of difference	-0.04286 to -0.0006081				
(B1 - A1) - (B2 - A2)	0,02173				
95% CI of difference	0.0006081 to 0.04286				
Normality of Residuals					
Test name	Statistics	P value	Passed normality test (alpha=0.05)?	P value summary	
D'Agostino-Pearson omnibus (K2)	0,3931	0,8216	Yes	ns	
Anderson-Darling (A2*)	0,3098	0,5117	Yes	ns	
Shapiro-Wilk (W)	0,9539	0,6578	Yes	ns	
Kolmogorov-Smirnov (distance)	0,1620	0,1000	Yes	ns	
Data summary					
Number of columns (Genotype)	2				
Number of rows (Injury)	2				
Number of values	13				

2way ANOVA of AEA pmol:g cortex

Table Analyzed	AEA pmol/g cortex				
Two-way ANOVA	Ordinary				
Alpha	0,05				
Source of Variation	% of total variation	P value	P value summary	Significant?	
Interaction	2,322	0,3989	ns	No	
Injury	20,71	0,0198	*	Yes	
Genotype	27,53	0,0088	**	Yes	
ANOVA table	SS	DF	MS	F (DFn, DFd)	P value
Interaction	489,0	1	489,0	F (1, 16) = 0.7514	P=0.3989
Injury	4360	1	4360	F (1, 16) = 6.701	P=0.0198
Genotype	5798	1	5798	F (1, 16) = 8.910	P=0.0088
Residual	10412	16	650,7		
Difference between column means					
Mean of WT	43,40				
Mean of APP-SWE	77,45				
Difference between means	-34,05				
SE of difference	11,41				
95% CI of difference	-58.24 to -9.869				
Difference between row means					
Mean of Sham	75,19				
Mean of mTBI	45,66				
Difference between means	29,53				
SE of difference	11,41				
95% CI of difference	5.347 to 53.72				
Interaction CI					
Mean diff, A1 - B1	-43,94				
Mean diff, A2 - B2	-24,16				
(A1 -B1) - (A2 - B2)	-19,78				
95% CI of difference	-68.15 to 28.59				
(B1 - A1) - (B2 - A2)	19,78				
95% CI of difference	-28.59 to 68.15				
Normality of Residuals					
Test name	Statistics	P value	Passed normality test (alpha=0.05)?	P value summary	
D'Agostino-Pearson omnibus (K2)	12,86	0,0016	No	**	
Anderson-Darling (A2*)	1,326	0,0014	No	**	
Shapiro-Wilk (W)	0,8237	0,0020	No	**	
Kolmogorov-Smirnov (distance)	0,2228	0,0104	No	*	
Data summary					

**Number of columns (Genotype)**	2				
**Number of rows (Injury)**	2				
**Number of values**	20				

2way ANOVA of 2-AG pmol:mg cortex

Table Analyzed	2-AG pmol/mg cortex				
Two-way ANOVA	Ordinary				
Alpha	0,05				
Source of Variation	% of total variation	P value	P value summary	Significant?	
Interaction	0,2764	0,7197	ns	No	
Injury	1,754	0,3711	ns	No	
Genotype	64,83	<0.0001	****	Yes	
ANOVA table	SS	DF	MS	F (DFn, DFd)	P value
Interaction	0,1248	1	0,1248	F (1, 16) = 0.1334	P=0.7197
Injury	0,7920	1	0,7920	F (1, 16) = 0.8468	P=0.3711
Genotype	29,28	1	29,28	F (1, 16) = 31.31	P<0.0001
Residual	14,97	16	0,9353		
Difference between column means					
Mean of WT	0,6600				
Mean of APP-SWE	3,080				
Difference between means	-2,420				
SE of difference	0,4325				
95% CI of difference	-3.337 to -1.503				
Difference between row means					
Mean of Sham	2,069				
Mean of mTBI	1,671				
Difference between means	0,3980				
SE of difference	0,4325				
95% CI of difference	-0.5189 to 1.315				
Interaction CI					
Mean diff, A1 - B1	-2,262				
Mean diff, A2 - B2	-2,578				
(A1 -B1) - (A2 - B2)	0,3160				
95% CI of difference	-1.518 to 2.150				
(B1 - A1) - (B2 - A2)	-0,3160				
95% CI of difference	-2.150 to 1.518				
Normality of Residuals					
Test name	Statistics	P value	Passed normality test (alpha=0.05)?	P value summary	
D'Agostino-Pearson omnibus (K2)	7,349	0,0254	No	*	
Anderson-Darling (A2*)	0,7249	0,0492	No	*	
Shapiro-Wilk (W)	0,9030	0,0470	No	*	
Kolmogorov-Smirnov (distance)	0,1813	0,0837	Yes	ns	
Data summary					
Number of columns (Genotype)	2				
Number of rows (Injury)	2				

**Number of values**	20				

2way ANOVA of PEA pmol:mg cortex

Table Analyzed	PEA pmol/mg cortex				
Two-way ANOVA	Ordinary				
Alpha	0,05				
Source of Variation	% of total variation	P value	P value summary	Significant?	
Interaction	1,460	0,5533	ns	No	
Injury	12,58	0,0945	ns	No	
Genotype	22,24	0,0311	*	Yes	
ANOVA table	SS	DF	MS	F (DFn, DFd)	P value
Interaction	0,004805	1	0,004805	F (1, 16) = 0.3667	P=0.5533
Injury	0,04141	1	0,04141	F (1, 16) = 3.159	P=0.0945
Genotype	0,07321	1	0,07321	F (1, 16) = 5.586	P=0.0311
Residual	0,2097	16	0,01311		
Difference between column means					
Mean of WT	0,3710				
Mean of APP-SWE	0,2500				
Difference between means	0,1210				
SE of difference	0,05120				
95% CI of difference	0.01247 to 0.2295				
Difference between row means					
Mean of Sham	0,3560				
Mean of mTBI	0,2650				
Difference between means	0,09100				
SE of difference	0,05120				
95% CI of difference	-0.01753 to 0.1995				
Interaction CI					
Mean diff, A1 - B1	0,1520				
Mean diff, A2 - B2	0,09000				
(A1 -B1) - (A2 - B2)	0,06200				
95% CI of difference	-0.1551 to 0.2791				
(B1 - A1) - (B2 - A2)	-0,06200				
95% CI of difference	-0.2791 to 0.1551				
Normality of Residuals					
Test name	Statistics	P value	Passed normality test (alpha=0.05)?	P value summary	
D'Agostino-Pearson omnibus (K2)	13,13	0,0014	No	**	
Anderson-Darling (A2*)	0,9306	0,0145	No	*	
Shapiro-Wilk (W)	0,8671	0,0105	No	*	
Kolmogorov-Smirnov (distance)	0,2121	0,0188	No	*	
Data summary					
Number of columns (Genotype)	2				

**Number of rows (Injury)**	2				
**Number of values**	20				

2way ANOVA of OEA pmol:mg cortex

Table Analyzed	OEA pmol/mg cortex				
Two-way ANOVA	Ordinary				
Alpha	0,05				
Source of Variation	% of total variation	P value	P value summary	Significant?	
Interaction	5,327	0,1518	ns	No	
Injury	9,109	0,0666	ns	No	
Genotype	47,94	0,0004	***	Yes	
ANOVA table	SS	DF	MS	F (DFn, DFd)	P value
Interaction	0,02113	1	0,02113	F (1, 16) = 2.265	P=0.1518
Injury	0,03613	1	0,03613	F (1, 16) = 3.874	P=0.0666
Genotype	0,1901	1	0,1901	F (1, 16) = 20.39	P=0.0004
Residual	0,1492	16	0,009325		
Difference between column means					
Mean of WT	0,2350				
Mean of APP-SWE	0,4300				
Difference between means	-0,1950				
SE of difference	0,04319				
95% CI of difference	-0.2865 to -0.1035				
Difference between row means					
Mean of Sham	0,3750				
Mean of mTBI	0,2900				
Difference between means	0,08500				
SE of difference	0,04319				
95% CI of difference	-0.006549 to 0.1765				
Interaction CI					
Mean diff, A1 - B1	-0,1300				
Mean diff, A2 - B2	-0,2600				
(A1 -B1) - (A2 - B2)	0,1300				
95% CI of difference	-0.05310 to 0.3131				
(B1 - A1) - (B2 - A2)	-0,1300				
95% CI of difference	-0.3131 to 0.05310				
Normality of Residuals					
Test name	Statistics	P value	Passed normality test (alpha=0.05)?	P value summary	
D'Agostino-Pearson omnibus (K2)	11,54	0,0031	No	**	
Anderson-Darling (A2*)	1,251	0,0022	No	**	
Shapiro-Wilk (W)	0,8574	0,0071	No	**	
Kolmogorov-Smirnov (distance)	0,2500	0,0020	No	**	
Data summary					
Number of columns (Genotype)	2				

**Number of rows (Injury)**	2				
**Number of values**	20				

2way ANOVA of DHEA pmol:mg cortex

Table Analyzed	DHEA pmol/mg cortex				
Two-way ANOVA	Ordinary				
Alpha	0,05				
Source of Variation	% of total variation	P value	P value summary	Significant?	
Interaction	0,01510	0,9548	ns	No	
Injury	4,720	0,3240	ns	No	
Genotype	22,34	0,0417	*	Yes	
ANOVA table	SS	DF	MS	F (DFn, DFd)	P value
Interaction	3,802E-05	1	3,802E-05	F (1, 16) = 0.003312	P=0.9548
Injury	0,01189	1	0,01189	F (1, 16) = 1.035	P=0.3240
Genotype	0,05626	1	0,05626	F (1, 16) = 4.901	P=0.0417
Residual	0,1837	16	0,01148		
Difference between column means					
Mean of WT	0,2430				
Mean of APP-SWE	0,3491				
Difference between means	-0,1061				
SE of difference	0,04792				
95% CI of difference	-0.2076 to -0.004496				
Difference between row means					
Mean of Sham	0,3204				
Mean of mTBI	0,2717				
Difference between means	0,04876				
SE of difference	0,04792				
95% CI of difference	-0.05282 to 0.1503				
Interaction CI					
Mean diff, A1 - B1	-0,1088				
Mean diff, A2 - B2	-0,1033				
(A1 -B1) - (A2 - B2)	-0,005515				
95% CI of difference	-0.2087 to 0.1976				
(B1 - A1) - (B2 - A2)	0,005515				
95% CI of difference	-0.1976 to 0.2087				
Normality of Residuals					
Test name	Statistics	P value	Passed normality test (alpha=0.05)?	P value summary	
D'Agostino-Pearson omnibus (K2)	2,619	0,2700	Yes	ns	
Anderson-Darling (A2*)	0,4596	0,2344	Yes	ns	
Shapiro-Wilk (W)	0,9361	0,2019	Yes	ns	
Kolmogorov-Smirnov (distance)	0,1404	0,1000	Yes	ns	
Data summary					
Number of columns (Genotype)	2				
Number of rows (Injury)	2				
Number of values	20				

2way ANOVA of EPEA pmol:mg cortex

Table Analyzed	EPEA pmol/mg cortex				
Two-way ANOVA	Ordinary				
Alpha	0,05				
Source of Variation	% of total variation	P value	P value summary	Significant?	
Interaction	13,31	0,0273	*	Yes	
Injury	26,31	0,0036	**	Yes	
Genotype	24,26	0,0047	**	Yes	
ANOVA table	SS	DF	MS	F (DFn, DFd)	P value
Interaction	0,0001468	1	0,0001468	F (1, 16) = 5.897	P=0.0273
Injury	0,0002902	1	0,0002902	F (1, 16) = 11.66	P=0.0036
Genotype	0,0002675	1	0,0002675	F (1, 16) = 10.75	P=0.0047
Residual	0,0003983	16	2,489E-05		
Difference between column means					
Mean of WT	0,006900				
Mean of APP-SWE	0,01421				
Difference between means	-0,007315				
SE of difference	0,002231				
95% CI of difference	-0.01204 to -0.002585				
Difference between row means					
Mean of Sham	0,01437				
Mean of mTBI	0,006748				
Difference between means	0,007618				
SE of difference	0,002231				
95% CI of difference	0.002888 to 0.01235				
Interaction CI					
Mean diff, A1 - B1	-0,01273				
Mean diff, A2 - B2	-0,001897				
(A1 -B1) - (A2 - B2)	-0,01084				
95% CI of difference	-0.02030 to -0.001377				
(B1 - A1) - (B2 - A2)	0,01084				
95% CI of difference	0.001377 to 0.02030				
Normality of Residuals					
Test name	Statistics	P value	Passed normality test (alpha=0.05)?	P value summary	
D'Agostino-Pearson omnibus (K2)	0,9544	0,6205	Yes	ns	
Anderson-Darling (A2*)	0,3318	0,4827	Yes	ns	
Shapiro-Wilk (W)	0,9605	0,5542	Yes	ns	
Kolmogorov-Smirnov (distance)	0,1326	0,1000	Yes	ns	
Data summary					
Number of columns (Genotype)	2				
Number of rows (Injury)	2				

**Number of values**	20				

2way ANOVA of 2-DHG pmol:mg cortex

Compare cell means regardless of rows and columns								
Number of families	1							
Number of comparisons per family	6							
Alpha	0,05							
Tukey's multiple comparisons test	Mean Diff.	95.00% CI of diff.	Below threshold?	Summary	Adjusted P Value			
Sham:WT vs. Sham:APP-SWE	-5,810	-19.19 to 7.573	No	ns	0,6105			
Sham:WT vs. mTBI:WT	-5,014	-18.40 to 8.369	No	ns	0,7109			
Sham:WT vs. mTBI:APP-SWE	0,6700	-12.71 to 14.05	No	ns	0,9989			
Sham:APP-SWE vs. mTBI:WT	0,7960	-12.59 to 14.18	No	ns	0,9982			
Sham:APP-SWE vs. mTBI:APP-SWE	6,480	-6.903 to 19.86	No	ns	0,5257			
mTBI:WT vs. mTBI:APP-SWE	5,684	-7.699 to 19.07	No	ns	0,6266			
Test details	Mean 1	Mean 2	Mean Diff.	SE of diff.	N1	N2	q	DF
Sham:WT vs. Sham:APP-SWE	11,97	17,78	-5,810	4,678	5	5	1,757	16,00
Sham:WT vs. mTBI:WT	11,97	16,98	-5,014	4,678	5	5	1,516	16,00
Sham:WT vs. mTBI:APP-SWE	11,97	11,30	0,6700	4,678	5	5	0,2026	16,00
Sham:APP-SWE vs. mTBI:WT	17,78	16,98	0,7960	4,678	5	5	0,2407	16,00
Sham:APP-SWE vs. mTBI:APP-SWE	17,78	11,30	6,480	4,678	5	5	1,959	16,00
mTBI:WT vs. mTBI:APP-SWE	16,98	11,30	5,684	4,678	5	5	1,718	16,00

2way ANOVA of OlGly pmol:mg cortex

Compare cell means regardless of rows and columns								
**Number of families**	1							
**Number of comparisons per family**	6							
**Alpha**	0,05							
**Tukey's multiple comparisons test**	Mean Diff.	95.00% CI of diff.	Below threshold?	Summary	Adjusted P Value			
**Sham:WT vs. Sham:APP-SWE**	-0,3860	-1.785 to 1.013	No	ns	0,8582			
**Sham:WT vs. mTBI:WT**	0,4740	-0.9251 to 1.873	No	ns	0,7684			
**Sham:WT vs. mTBI:APP-SWE**	-0,3060	-1.705 to 1.093	No	ns	0,9223			
**Sham:APP-SWE vs. mTBI:WT**	0,8600	-0.5391 to 2.259	No	ns	0,3279			
**Sham:APP-SWE vs. mTBI:APP-SWE**	0,08000	-1.319 to 1.479	No	ns	0,9984			
**mTBI:WT vs. mTBI:APP-SWE**	-0,7800	-2.179 to 0.6191	No	ns	0,4088			
**Test details**	Mean 1	Mean 2	Mean Diff.	SE of diff.	N1	N2	q	DF
**Sham:WT vs. Sham:APP-SWE**	1,634	2,020	-0,3860	0,4890	5	5	1,116	16,00
**Sham:WT vs. mTBI:WT**	1,634	1,160	0,4740	0,4890	5	5	1,371	16,00
**Sham:WT vs. mTBI:APP-SWE**	1,634	1,940	-0,3060	0,4890	5	5	0,8849	16,00
**Sham:APP-SWE vs. mTBI:WT**	2,020	1,160	0,8600	0,4890	5	5	2,487	16,00
**Sham:APP-SWE vs. mTBI:APP-SWE**	2,020	1,940	0,08000	0,4890	5	5	0,2313	16,00
**mTBI:WT vs. mTBI:APP-SWE**	1,160	1,940	-0,7800	0,4890	5	5	2,256	16,00

2way ANOVA of OA5HT pmol:mg cortex

Table Analyzed	OA5HT pmol/mg cortex				
Two-way ANOVA	Ordinary				
Alpha	0,05				
Source of Variation	% of total variation	P value	P value summary	Significant?	
Interaction	30,68	0,0006	***	Yes	
Injury	30,92	0,0006	***	Yes	
Genotype	33,14	0,0005	***	Yes	
ANOVA table	SS (Type III)	DF	MS	F (DFn, DFd)	P value
Interaction	813,6	1	813,6	F (1, 12) = 20.95	P=0.0006
Injury	820,0	1	820,0	F (1, 12) = 21.12	P=0.0006
Genotype	878,8	1	878,8	F (1, 12) = 22.64	P=0.0005
Residual	465,9	12	38,83		
Difference between column means					
Predicted (LS) mean of WT	15,66				
Predicted (LS) mean of APP-SWE	0,3530				
Difference between predicted means	15,31				
SE of difference	3,218				
95% CI of difference	8.298 to 22.32				
Difference between row means					
Predicted (LS) mean of Sham	0,6137				
Predicted (LS) mean of mTBI	15,40				
Difference between predicted means	-14,79				
SE of difference	3,218				
95% CI of difference	-21.80 to -7.777				
Interaction CI					
Mean diff, A1 - B1	0,5793				
Mean diff, A2 - B2	30,04				
(A1 -B1) - (A2 - B2)	-29,46				
95% CI of difference	-43.48 to -15.44				
(B1 - A1) - (B2 - A2)	29,46				
95% CI of difference	15.44 to 43.48				
Normality of Residuals					
Test name	Statistics	P value	Passed normality test (alpha=0.05)?	P value summary	
D'Agostino-Pearson omnibus (K2)	19,11	<0.0001	No	****	
Anderson-Darling (A2*)	3,030	<0.0001	No	****	
Shapiro-Wilk (W)	0,6034	<0.0001	No	****	
Kolmogorov-Smirnov (distance)	0,3963	<0.0001	No	****	
Data summary					
Number of columns (Genotype)	2				
Number of rows (Injury)	2				
Number of values	16				

2way ANOVA of DHA5HT pmol:mg cortex

Table Analyzed	DHA5HT pmol/mg cortex				
Two-way ANOVA	Ordinary				
Alpha	0,05				
Source of Variation	% of total variation	P value	P value summary	Significant?	
Interaction	23,55	0,0194	*	Yes	
Injury	23,32	0,0199	*	Yes	
Genotype	25,97	0,0152	*	Yes	
ANOVA table	SS (Type III)	DF	MS	F (DFn, DFd)	P value
Interaction	4146	1	4146	F (1, 11) = 7.475	P=0.0194
Injury	4106	1	4106	F (1, 11) = 7.404	P=0.0199
Genotype	4572	1	4572	F (1, 11) = 8.244	P=0.0152
Residual	6101	11	554,6		
Difference between column means					
Predicted (LS) mean of WT	36,24				
Predicted (LS) mean of APP-SWE	0,5110				
Difference between predicted means	35,73				
SE of difference	12,44				
95% CI of difference	8.341 to 63.11				
Difference between row means					
Predicted (LS) mean of Sham	1,446				
Predicted (LS) mean of mTBI	35,30				
Difference between predicted means	-33,86				
SE of difference	12,44				
95% CI of difference	-61.24 to -6.471				
Interaction CI					
Mean diff, A1 - B1	1,708				
Mean diff, A2 - B2	69,75				
(A1 -B1) - (A2 - B2)	-68,04				
95% CI of difference	-122.8 to -13.27				
(B1 - A1) - (B2 - A2)	68,04				
95% CI of difference	13.27 to 122.8				
Normality of Residuals					
Test name	Statistics	P value	Passed normality test (alpha=0.05)?	P value summary	
D'Agostino-Pearson omnibus (K2)	13,72	0,0010	No	**	
Anderson-Darling (A2*)	2,880	<0.0001	No	****	
Shapiro-Wilk (W)	0,6227	<0.0001	No	****	
Kolmogorov-Smirnov (distance)	0,3982	<0.0001	No	****	
Data summary					
Number of columns (Genotype)	2				
Number of rows (Injury)	2				
Number of values	15				

2way ANOVA of EPA5HT pmol:mg cortex

Table Analyzed	EPA5HT pmol/mg cortex				
Two-way ANOVA	Ordinary				
Alpha	0,05				
Source of Variation	% of total variation	P value	P value summary	Significant?	
Interaction	2,347	0,4340	ns	No	
Injury	3,059	0,3733	ns	No	
Genotype	37,86	0,0056	**	Yes	
ANOVA table	SS (Type III)	DF	MS	F (DFn, DFd)	P value
Interaction	0,7361	1	0,7361	F (1, 15) = 0.6462	P=0.4340
Injury	0,9593	1	0,9593	F (1, 15) = 0.8421	P=0.3733
Genotype	11,87	1	11,87	F (1, 15) = 10.42	P=0.0056
Residual	17,09	15	1,139		
Difference between column means					
Predicted (LS) mean of WT	2,869				
Predicted (LS) mean of APP-SWE	1,280				
Difference between predicted means	1,589				
SE of difference	0,4920				
95% CI of difference	0.5398 to 2.637				
Difference between row means					
Predicted (LS) mean of Sham	2,300				
Predicted (LS) mean of mTBI	1,849				
Difference between predicted means	0,4515				
SE of difference	0,4920				
95% CI of difference	-0.5972 to 1.500				
Interaction CI					
Mean diff, A1 - B1	1,984				
Mean diff, A2 - B2	1,193				
(A1 -B1) - (A2 - B2)	0,7910				
95% CI of difference	-1.306 to 2.888				
(B1 - A1) - (B2 - A2)	-0,7910				
95% CI of difference	-2.888 to 1.306				
Normality of Residuals					
Test name	Statistics	P value	Passed normality test (alpha=0.05)?	P value summary	
D'Agostino-Pearson omnibus (K2)	1,022	0,5999	Yes	ns	
Anderson-Darling (A2*)	0,2116	0,8319	Yes	ns	
Shapiro-Wilk (W)	0,9809	0,9525	Yes	ns	
Kolmogorov-Smirnov (distance)	0,1041	0,1000	Yes	ns	
Data summary					
Number of columns (Genotype)	2				
Number of rows (Injury)	2				
Number of values	19				

2way ANOVA of PA5HT pmol:mg cortex

Compare cell means regardless of rows and columns								
**Number of families**	1							
**Number of comparisons per family**	6							
**Alpha**	0,05							
**Tukey's multiple comparisons test**	Predicted (LS) mean diff.	95.00% CI of diff.	Below threshold?	Summary	Adjusted P Value			
**Sham:WT vs. Sham:APP-SWE**	-21,60	-58.52 to 15.32	No	ns	0,3596			
**Sham:WT vs. mTBI:WT**	-0,5885	-37.51 to 36.33	No	ns	>0.9999			
**Sham:WT vs. mTBI:APP-SWE**	-1,388	-40.30 to 37.53	No	ns	0,9996			
**Sham:APP-SWE vs. mTBI:WT**	21,01	-13.80 to 55.82	No	ns	0,3342			
**Sham:APP-SWE vs. mTBI:APP-SWE**	20,21	-16.71 to 57.13	No	ns	0,4143			
**mTBI:WT vs. mTBI:APP-SWE**	-0,7990	-37.72 to 36.12	No	ns	>0.9999			
**Test details**	Predicted (LS) mean 1	Predicted (LS) mean 2	Predicted (LS) mean diff.	SE of diff.	N1	N2	q	DF
**Sham:WT vs. Sham:APP-SWE**	14,12	35,72	-21,60	12,70	4	5	2,405	14,00
**Sham:WT vs. mTBI:WT**	14,12	14,71	-0,5885	12,70	4	5	0,06552	14,00
**Sham:WT vs. mTBI:APP-SWE**	14,12	15,51	-1,388	13,39	4	4	0,1466	14,00
**Sham:APP-SWE vs. mTBI:WT**	35,72	14,71	21,01	11,98	5	5	2,481	14,00
**Sham:APP-SWE vs. mTBI:APP-SWE**	35,72	15,51	20,21	12,70	5	4	2,250	14,00
**mTBI:WT vs. mTBI:APP-SWE**	14,71	15,51	-0,7990	12,70	5	4	0,08896	14,00

2way ANOVA of AEA pmol:g hipp

Compare cell means regardless of rows and columns								
Number of families	1							
Number of comparisons per family	6							
Alpha	0,05							
Tukey's multiple comparisons test	Mean Diff.	95.00% CI of diff.	Below threshold?	Summary	Adjusted P Value			
Sham:WT vs. Sham:APP-SWE	-30,66	-102.3 to 41.01	No	ns	0,6213			
Sham:WT vs. mTBI:WT	22,62	-49.04 to 94.29	No	ns	0,8034			
Sham:WT vs. mTBI:APP-SWE	0,05800	-71.61 to 71.73	No	ns	>0.9999			
Sham:APP-SWE vs. mTBI:WT	53,29	-18.38 to 125.0	No	ns	0,1866			
Sham:APP-SWE vs. mTBI:APP-SWE	30,72	-40.95 to 102.4	No	ns	0,6199			
mTBI:WT vs. mTBI:APP-SWE	-22,57	-94.23 to 49.10	No	ns	0,8045			
Test details	Mean 1	Mean 2	Mean Diff.	SE of diff.	N1	N2	q	DF
Sham:WT vs. Sham:APP-SWE	141,4	172,0	-30,66	25,05	5	5	1,731	16,00
Sham:WT vs. mTBI:WT	141,4	118,7	22,62	25,05	5	5	1,277	16,00
Sham:WT vs. mTBI:APP-SWE	141,4	141,3	0,05800	25,05	5	5	0,003274	16,00
Sham:APP-SWE vs. mTBI:WT	172,0	118,7	53,29	25,05	5	5	3,008	16,00
Sham:APP-SWE vs. mTBI:APP-SWE	172,0	141,3	30,72	25,05	5	5	1,734	16,00
mTBI:WT vs. mTBI:APP-SWE	118,7	141,3	-22,57	25,05	5	5	1,274	16,00

2way ANOVA of 2-AG pmol:mg hipp

Table Analyzed	2-AG pmol/mg hipp				
Two-way ANOVA	Ordinary				
Alpha	0,05				
Source of Variation	% of total variation	P value	P value summary	Significant?	
Interaction	2,486	0,2096	ns	No	
Injury	0,06086	0,8405	ns	No	
Genotype	74,18	<0.0001	****	Yes	
ANOVA table	SS	DF	MS	F (DFn, DFd)	P value
Interaction	0,4322	1	0,4322	F (1, 16) = 1.709	P=0.2096
Injury	0,01058	1	0,01058	F (1, 16) = 0.04184	P=0.8405
Genotype	12,90	1	12,90	F (1, 16) = 51.00	P<0.0001
Residual	4,046	16	0,2529		
Difference between column means					
Mean of WT	0,7840				
Mean of APP-SWE	2,390				
Difference between means	-1,606				
SE of difference	0,2249				
95% CI of difference	-2.083 to -1.129				
Difference between row means					
Mean of Sham	1,564				
Mean of mTBI	1,610				
Difference between means	-0,04600				
SE of difference	0,2249				
95% CI of difference	-0.5227 to 0.4307				
Interaction CI					
Mean diff, A1 - B1	-1,312				
Mean diff, A2 - B2	-1,900				
(A1 -B1) - (A2 - B2)	0,5880				
95% CI of difference	-0.3655 to 1.541				
(B1 - A1) - (B2 - A2)	-0,5880				
95% CI of difference	-1.541 to 0.3655				
Normality of Residuals					
Test name	Statistics	P value	Passed normality test (alpha=0.05)?	P value summary	
D'Agostino-Pearson omnibus (K2)	1,023	0,5996	Yes	ns	
Anderson-Darling (A2*)	0,1955	0,8754	Yes	ns	
Shapiro-Wilk (W)	0,9736	0,8275	Yes	ns	
Kolmogorov-Smirnov (distance)	0,08118	0,1000	Yes	ns	
Data summary					
Number of columns (Genotype)	2				
Number of rows (Injury)	2				

**Number of values**	20				

2way ANOVA of PEA pmol:mg hipp

Table Analyzed	PEA pmol/mg hipp				
Two-way ANOVA	Ordinary				
Alpha	0,05				
Source of Variation	% of total variation	P value	P value summary	Significant?	
Interaction	1,210	0,5559	ns	No	
Injury	3,706	0,3085	ns	No	
Genotype	47,27	0,0019	**	Yes	
ANOVA table	SS (Type III)	DF	MS	F (DFn, DFd)	P value
Interaction	0,03012	1	0,03012	F (1, 15) = 0.3628	P=0.5559
Injury	0,09224	1	0,09224	F (1, 15) = 1.111	P=0.3085
Genotype	1,176	1	1,176	F (1, 15) = 14.17	P=0.0019
Residual	1,245	15	0,08301		
Difference between column means					
Predicted (LS) mean of WT	0,8100				
Predicted (LS) mean of APP-SWE	0,3100				
Difference between predicted means	0,5000				
SE of difference	0,1328				
95% CI of difference	0.2169 to 0.7831				
Difference between row means					
Predicted (LS) mean of Sham	0,6300				
Predicted (LS) mean of mTBI	0,4900				
Difference between predicted means	0,1400				
SE of difference	0,1328				
95% CI of difference	-0.1431 to 0.4231				
Interaction CI					
Mean diff, A1 - B1	0,5800				
Mean diff, A2 - B2	0,4200				
(A1 -B1) - (A2 - B2)	0,1600				
95% CI of difference	-0.4062 to 0.7262				
(B1 - A1) - (B2 - A2)	-0,1600				
95% CI of difference	-0.7262 to 0.4062				
Normality of Residuals					
Test name	Statistics	P value	Passed normality test (alpha=0.05)?	P value summary	
D'Agostino-Pearson omnibus (K2)	17,52	0,0002	No	***	
Anderson-Darling (A2*)	0,9630	0,0118	No	*	
Shapiro-Wilk (W)	0,8426	0,0051	No	**	
Kolmogorov-Smirnov (distance)	0,2446	0,0040	No	**	
Data summary					
Number of columns (Genotype)	2				
Number of rows (Injury)	2				
Number of values	19				

2way ANOVA of OEA pmol:mg hipp

Compare cell means regardless of rows and columns								
Number of families	1							
Number of comparisons per family	6							
Alpha	0,05							
Tukey's multiple comparisons test	Mean Diff.	95.00% CI of diff.	Below threshold?	Summary	Adjusted P Value			
Sham:WT vs. Sham:APP-SWE	-0,3400	-0.6965 to 0.01650	No	ns	0,0643			
Sham:WT vs. mTBI:WT	-0,02800	-0.3845 to 0.3285	No	ns	0,9958			
Sham:WT vs. mTBI:APP-SWE	-0,1200	-0.4765 to 0.2365	No	ns	0,7718			
Sham:APP-SWE vs. mTBI:WT	0,3120	-0.04450 to 0.6685	No	ns	0,0974			
Sham:APP-SWE vs. mTBI:APP-SWE	0,2200	-0.1365 to 0.5765	No	ns	0,3247			
mTBI:WT vs. mTBI:APP-SWE	-0,09200	-0.4485 to 0.2645	No	ns	0,8802			
Test details	Mean 1	Mean 2	Mean Diff.	SE of diff.	N1	N2	q	DF
Sham:WT vs. Sham:APP-SWE	0,4200	0,7600	-0,3400	0,1246	5	5	3,859	16,00
Sham:WT vs. mTBI:WT	0,4200	0,4480	-0,02800	0,1246	5	5	0,3178	16,00
Sham:WT vs. mTBI:APP-SWE	0,4200	0,5400	-0,1200	0,1246	5	5	1,362	16,00
Sham:APP-SWE vs. mTBI:WT	0,7600	0,4480	0,3120	0,1246	5	5	3,541	16,00
Sham:APP-SWE vs. mTBI:APP-SWE	0,7600	0,5400	0,2200	0,1246	5	5	2,497	16,00
mTBI:WT vs. mTBI:APP-SWE	0,4480	0,5400	-0,09200	0,1246	5	5	1,044	16,00

2way ANOVA of DHEA pmol:mg hipp

Compare cell means regardless of rows and columns								
**Number of families**	1							
**Number of comparisons per family**	6							
**Alpha**	0,05							
**Tukey's multiple comparisons test**	Mean Diff.	95.00% CI of diff.	Below threshold?	Summary	Adjusted P Value			
**Sham:WT vs. Sham:APP-SWE**	-0,2984	-0.5937 to -0.003057	Yes	*	0,0472			
**Sham:WT vs. mTBI:WT**	0,07800	-0.2173 to 0.3733	No	ns	0,8729			
**Sham:WT vs. mTBI:APP-SWE**	-0,07492	-0.3702 to 0.2204	No	ns	0,8854			
**Sham:APP-SWE vs. mTBI:WT**	0,3764	0.08106 to 0.6717	Yes	*	0,0105			
**Sham:APP-SWE vs. mTBI:APP-SWE**	0,2235	-0.07186 to 0.5188	No	ns	0,1754			
**mTBI:WT vs. mTBI:APP-SWE**	-0,1529	-0.4482 to 0.1424	No	ns	0,4706			
**Test details**	Mean 1	Mean 2	Mean Diff.	SE of diff.	N1	N2	q	DF
**Sham:WT vs. Sham:APP-SWE**	0,3640	0,6624	-0,2984	0,1032	5	5	4,088	16,00
**Sham:WT vs. mTBI:WT**	0,3640	0,2860	0,07800	0,1032	5	5	1,069	16,00
**Sham:WT vs. mTBI:APP-SWE**	0,3640	0,4389	-0,07492	0,1032	5	5	1,026	16,00
**Sham:APP-SWE vs. mTBI:WT**	0,6624	0,2860	0,3764	0,1032	5	5	5,157	16,00
**Sham:APP-SWE vs. mTBI:APP-SWE**	0,6624	0,4389	0,2235	0,1032	5	5	3,062	16,00
**mTBI:WT vs. mTBI:APP-SWE**	0,2860	0,4389	-0,1529	0,1032	5	5	2,095	16,00

2way ANOVA of EPEA pmol:mg hipp

Compare cell means regardless of rows and columns								
**Number of families**	1							
**Number of comparisons per family**	6							
**Alpha**	0,05							
**Tukey's multiple comparisons test**	Mean Diff.	95.00% CI of diff.	Below threshold?	Summary	Adjusted P Value			
**Sham:WT vs. Sham:APP-SWE**	-0,001461	-0.01513 to 0.01221	No	ns	0,9897			
**Sham:WT vs. mTBI:WT**	0,006000	-0.007672 to 0.01967	No	ns	0,6024			
**Sham:WT vs. mTBI:APP-SWE**	0,002738	-0.01093 to 0.01641	No	ns	0,9387			
**Sham:APP-SWE vs. mTBI:WT**	0,007461	-0.006211 to 0.02113	No	ns	0,4268			
**Sham:APP-SWE vs. mTBI:APP-SWE**	0,004199	-0.009473 to 0.01787	No	ns	0,8158			
**mTBI:WT vs. mTBI:APP-SWE**	-0,003262	-0.01693 to 0.01041	No	ns	0,9022			
**Test details**	Mean 1	Mean 2	Mean Diff.	SE of diff.	N1	N2	q	DF
**Sham:WT vs. Sham:APP-SWE**	0,02000	0,02146	-0,001461	0,004779	5	5	0,4323	16,00
**Sham:WT vs. mTBI:WT**	0,02000	0,01400	0,006000	0,004779	5	5	1,776	16,00
**Sham:WT vs. mTBI:APP-SWE**	0,02000	0,01726	0,002738	0,004779	5	5	0,8103	16,00
**Sham:APP-SWE vs. mTBI:WT**	0,02146	0,01400	0,007461	0,004779	5	5	2,208	16,00
**Sham:APP-SWE vs. mTBI:APP-SWE**	0,02146	0,01726	0,004199	0,004779	5	5	1,243	16,00
**mTBI:WT vs. mTBI:APP-SWE**	0,01400	0,01726	-0,003262	0,004779	5	5	0,9654	16,00

2way ANOVA of 2-DHG pmol:mg hipp

Compare cell means regardless of rows and columns								
**Number of families**	1							
**Number of comparisons per family**	6							
**Alpha**	0,05							
**Tukey's multiple comparisons test**	Predicted (LS) mean diff.	95.00% CI of diff.	Below threshold?	Summary	Adjusted P Value			
**Sham:WT vs. Sham:APP-SWE**	-14,42	-38.98 to 10.15	No	ns	0,3518			
**Sham:WT vs. mTBI:WT**	19,33	-5.236 to 43.89	No	ns	0,1463			
**Sham:WT vs. mTBI:APP-SWE**	-5,222	-33.19 to 22.75	No	ns	0,9455			
**Sham:APP-SWE vs. mTBI:WT**	33,74	10.58 to 56.90	Yes	**	0,0044			
**Sham:APP-SWE vs. mTBI:APP-SWE**	9,193	-17.55 to 35.94	No	ns	0,7471			
**mTBI:WT vs. mTBI:APP-SWE**	-24,55	-51.29 to 2.193	No	ns	0,0764			
**Test details**	Predicted (LS) mean 1	Predicted (LS) mean 2	Predicted (LS) mean diff.	SE of diff.	N1	N2	q	DF
**Sham:WT vs. Sham:APP-SWE**	39,95	54,36	-14,42	8,370	4	5	2,436	13,00
**Sham:WT vs. mTBI:WT**	39,95	20,62	19,33	8,370	4	5	3,266	13,00
**Sham:WT vs. mTBI:APP-SWE**	39,95	45,17	-5,222	9,529	4	3	0,7749	13,00
**Sham:APP-SWE vs. mTBI:WT**	54,36	20,62	33,74	7,891	5	5	6,048	13,00
**Sham:APP-SWE vs. mTBI:APP-SWE**	54,36	45,17	9,193	9,112	5	3	1,427	13,00
**mTBI:WT vs. mTBI:APP-SWE**	20,62	45,17	-24,55	9,112	5	3	3,811	13,00

2way ANOVA of OlGly pmol:mg hipp

Table Analyzed	OlGly pmol/mg hipp				
Two-way ANOVA	Ordinary				
Alpha	0,05				
Source of Variation	% of total variation	P value	P value summary	Significant?	
Interaction	21,49	0,0236	*	Yes	
Injury	0,09831	0,8661	ns	No	
Genotype	31,80	0,0080	**	Yes	
ANOVA table	SS (Type III)	DF	MS	F (DFn, DFd)	P value
Interaction	6,413	1	6,413	F (1, 14) = 6.447	P=0.0236
Injury	0,02934	1	0,02934	F (1, 14) = 0.02949	P=0.8661
Genotype	9,490	1	9,490	F (1, 14) = 9.539	P=0.0080
Residual	13,93	14	0,9948		
Difference between column means					
Predicted (LS) mean of WT	1,859				
Predicted (LS) mean of APP-SWE	3,320				
Difference between predicted means	-1,461				
SE of difference	0,4731				
95% CI of difference	-2.476 to -0.4465				
Difference between row means					
Predicted (LS) mean of Sham	2,630				
Predicted (LS) mean of mTBI	2,549				
Difference between predicted means	0,08125				
SE of difference	0,4731				
95% CI of difference	-0.9335 to 1.096				
Interaction CI					
Mean diff, A1 - B1	-0,2600				
Mean diff, A2 - B2	-2,663				
(A1 -B1) - (A2 - B2)	2,403				
95% CI of difference	0.3730 to 4.432				
(B1 - A1) - (B2 - A2)	-2,403				
95% CI of difference	-4.432 to -0.3730				
Normality of Residuals					
Test name	Statistics	P value	Passed normality test (alpha=0.05)?	P value summary	
D'Agostino-Pearson omnibus (K2)	0,3562	0,8369	Yes	ns	
Anderson-Darling (A2*)	0,1425	0,9638	Yes	ns	
Shapiro-Wilk (W)	0,9819	0,9675	Yes	ns	
Kolmogorov-Smirnov (distance)	0,09085	0,1000	Yes	ns	
Data summary					
Number of columns (Genotype)	2				
Number of rows (Injury)	2				
Number of values	18				

2way ANOVA of OA5HT pmol:mg hipp

Table Analyzed	OA5HT pmol/mg hipp				
Two-way ANOVA	Ordinary				
Alpha	0,05				
Source of Variation	% of total variation	P value	P value summary	Significant?	
Interaction	37,54	0,0065	**	Yes	
Injury	2,450	0,4280	ns	No	
Genotype	7,535	0,1742	ns	No	
ANOVA table	SS (Type III)	DF	MS	F (DFn, DFd)	P value
Interaction	11,49	1	11,49	F (1, 14) = 10.21	P=0.0065
Injury	0,7500	1	0,7500	F (1, 14) = 0.6663	P=0.4280
Genotype	2,307	1	2,307	F (1, 14) = 2.049	P=0.1742
Residual	15,76	14	1,126		
Difference between column means					
Predicted (LS) mean of WT	0,9063				
Predicted (LS) mean of APP-SWE	1,640				
Difference between predicted means	-0,7337				
SE of difference	0,5125				
95% CI of difference	-1.833 to 0.3655				
Difference between row means					
Predicted (LS) mean of Sham	1,064				
Predicted (LS) mean of mTBI	1,482				
Difference between predicted means	-0,4183				
SE of difference	0,5125				
95% CI of difference	-1.518 to 0.6809				
Interaction CI					
Mean diff, A1 - B1	0,9040				
Mean diff, A2 - B2	-2,371				
(A1 -B1) - (A2 - B2)	3,275				
95% CI of difference	1.077 to 5.474				
(B1 - A1) - (B2 - A2)	-3,275				
95% CI of difference	-5.474 to -1.077				
Normality of Residuals					
Test name	Statistics	P value	Passed normality test (alpha=0.05)?	P value summary	
D'Agostino-Pearson omnibus (K2)	1,469	0,4798	Yes	ns	
Anderson-Darling (A2*)	0,4308	0,2734	Yes	ns	
Shapiro-Wilk (W)	0,9332	0,2212	Yes	ns	
Kolmogorov-Smirnov (distance)	0,1795	0,1000	Yes	ns	
Data summary					
Number of columns (Genotype)	2				
Number of rows (Injury)	2				
Number of values	18				

2way ANOVA of DHA5HT pmol:mg hipp

Compare cell means regardless of rows and columns								
Number of families	1							
Number of comparisons per family	6							
Alpha	0,05							
Tukey's multiple comparisons test	Predicted (LS) mean diff.	95.00% CI of diff.	Below threshold?	Summary	Adjusted P Value			
Sham:WT vs. Sham:APP-SWE	-0,5725	-2.177 to 1.032	No	ns	0,7256			
Sham:WT vs. mTBI:WT	0,7275	-1.099 to 2.554	No	ns	0,6556			
Sham:WT vs. mTBI:APP-SWE	-0,9905	-2.595 to 0.6136	No	ns	0,3112			
Sham:APP-SWE vs. mTBI:WT	1,300	-0.4463 to 3.046	No	ns	0,1787			
Sham:APP-SWE vs. mTBI:APP-SWE	-0,4180	-1.930 to 1.094	No	ns	0,8481			
mTBI:WT vs. mTBI:APP-SWE	-1,718	-3.464 to 0.02829	No	ns	0,0544			
Test details	Predicted (LS) mean 1	Predicted (LS) mean 2	Predicted (LS) mean diff.	SE of diff.	N1	N2	q	DF
Sham:WT vs. Sham:APP-SWE	1,498	2,070	-0,5725	0,5465	4	5	1,481	13,00
Sham:WT vs. mTBI:WT	1,498	0,7700	0,7275	0,6222	4	3	1,653	13,00
Sham:WT vs. mTBI:APP-SWE	1,498	2,488	-0,9905	0,5465	4	5	2,563	13,00
Sham:APP-SWE vs. mTBI:WT	2,070	0,7700	1,300	0,5950	5	3	3,090	13,00
Sham:APP-SWE vs. mTBI:APP-SWE	2,070	2,488	-0,4180	0,5153	5	5	1,147	13,00
mTBI:WT vs. mTBI:APP-SWE	0,7700	2,488	-1,718	0,5950	3	5	4,084	13,00

2way ANOVA of EPA5HT pmol:mg hipp

Table Analyzed	EPA5HT pmol/mg hipp				
Two-way ANOVA	Ordinary				
Alpha	0,05				
Source of Variation	% of total variation	P value	P value summary	Significant?	
Interaction	26,17	0,0072	**	Yes	
Injury	18,39	0,0196	*	Yes	
Genotype	22,73	0,0110	*	Yes	
ANOVA table	SS (Type III)	DF	MS	F (DFn, DFd)	P value
Interaction	211,2	1	211,2	F (1, 14) = 9.879	P=0.0072
Injury	148,4	1	148,4	F (1, 14) = 6.944	P=0.0196
Genotype	183,5	1	183,5	F (1, 14) = 8.581	P=0.0110
Residual	299,3	14	21,38		
Difference between column means					
Predicted (LS) mean of WT	12,39				
Predicted (LS) mean of APP-SWE	5,969				
Difference between predicted means	6,425				
SE of difference	2,193				
95% CI of difference	1.721 to 11.13				
Difference between row means					
Predicted (LS) mean of Sham	12,07				
Predicted (LS) mean of mTBI	6,292				
Difference between predicted means	5,779				
SE of difference	2,193				
95% CI of difference	1.075 to 10.48				
Interaction CI					
Mean diff, A1 - B1	13,32				
Mean diff, A2 - B2	-0,4685				
(A1 -B1) - (A2 - B2)	13,79				
95% CI of difference	4.379 to 23.19				
(B1 - A1) - (B2 - A2)	-13,79				
95% CI of difference	-23.19 to -4.379				
Normality of Residuals					
Test name	Statistics	P value	Passed normality test (alpha=0.05)?	P value summary	
D'Agostino-Pearson omnibus (K2)	2,396	0,3018	Yes	ns	
Anderson-Darling (A2*)	0,3726	0,3811	Yes	ns	
Shapiro-Wilk (W)	0,9436	0,3331	Yes	ns	
Kolmogorov-Smirnov (distance)	0,1436	0,1000	Yes	ns	
Data summary					
Number of columns (Genotype)	2				
Number of rows (Injury)	2				
Number of values	18				

2way ANOVA of PA5HT pmol:mg hipp

Table Analyzed	PA5HT pmol/mg hipp				
Two-way ANOVA	Ordinary				
Alpha	0,05				
Source of Variation	% of total variation	P value	P value summary	Significant?	
Interaction	3,317	0,2164	ns	No	
Injury	58,07	0,0001	***	Yes	
Genotype	11,95	0,0283	*	Yes	
ANOVA table	SS (Type III)	DF	MS	F (DFn, DFd)	P value
Interaction	341,4	1	341,4	F (1, 13) = 1.688	P=0.2164
Injury	5977	1	5977	F (1, 13) = 29.56	P=0.0001
Genotype	1231	1	1231	F (1, 13) = 6.085	P=0.0283
Residual	2629	13	202,2		
Difference between column means					
Predicted (LS) mean of WT	69,20				
Predicted (LS) mean of APP-SWE	52,11				
Difference between predicted means	17,10				
SE of difference	6,930				
95% CI of difference	2.123 to 32.07				
Difference between row means					
Predicted (LS) mean of Sham	79,50				
Predicted (LS) mean of mTBI	41,82				
Difference between predicted means	37,68				
SE of difference	6,930				
95% CI of difference	22.71 to 52.65				
Interaction CI					
Mean diff, A1 - B1	26,10				
Mean diff, A2 - B2	8,091				
(A1 -B1) - (A2 - B2)	18,01				
95% CI of difference	-11.94 to 47.95				
(B1 - A1) - (B2 - A2)	-18,01				
95% CI of difference	-47.95 to 11.94				
Normality of Residuals					
Test name	Statistics	P value	Passed normality test (alpha=0.05)?	P value summary	
D'Agostino-Pearson omnibus (K2)	0,5143	0,7732	Yes	ns	
Anderson-Darling (A2*)	0,2014	0,8566	Yes	ns	
Shapiro-Wilk (W)	0,9778	0,9346	Yes	ns	
Kolmogorov-Smirnov (distance)	0,1161	0,1000	Yes	ns	
Data summary					
Number of columns (Genotype)	2				
Number of rows (Injury)	2				
Number of values	17				

2way ANOVA of Napepld cortex

Table Analyzed	Napepld cortex				
Two-way ANOVA	Ordinary				
Alpha	0,05				
Source of Variation	% of total variation	P value	P value summary	Significant?	
Interaction	49,74	0,0005	***	Yes	
Injury	1,368	0,4840	ns	No	
Genotype	0,6904	0,6177	ns	No	
ANOVA table	SS (Type III)	DF	MS	F (DFn, DFd)	P value
Interaction	2,526	1	2,526	F (1, 16) = 18.67	P=0.0005
Injury	0,06945	1	0,06945	F (1, 16) = 0.5133	P=0.4840
Genotype	0,03506	1	0,03506	F (1, 16) = 0.2591	P=0.6177
Residual	2,165	16	0,1353		
Difference between column means					
Predicted (LS) mean of WT	0,5771				
Predicted (LS) mean of APP-SWE	0,6626				
Difference between predicted means	-0,08546				
SE of difference	0,1679				
95% CI of difference	-0.4414 to 0.2705				
Difference between row means					
Predicted (LS) mean of Sham	0,6800				
Predicted (LS) mean of mTBI	0,5597				
Difference between predicted means	0,1203				
SE of difference	0,1679				
95% CI of difference	-0.2356 to 0.4762				
Interaction CI					
Mean diff, A1 - B1	0,6400				
Mean diff, A2 - B2	-0,8109				
(A1 -B1) - (A2 - B2)	1,451				
95% CI of difference	0.7390 to 2.163				
(B1 - A1) - (B2 - A2)	-1,451				
95% CI of difference	-2.163 to -0.7390				
Normality of Residuals					
Test name	Statistics	P value	Passed normality test (alpha=0.05)?	P value summary	
D'Agostino-Pearson omnibus (K2)	4,212	0,1217	Yes	ns	
Anderson-Darling (A2*)	1,024	0,0084	No	**	
Shapiro-Wilk (W)	0,8864	0,0231	No	*	
Kolmogorov-Smirnov (distance)	0,1895	0,0579	Yes	ns	
Data summary					
Number of columns (Genotype)	2				
Number of rows (Injury)	2				

**Number of values**	20				

2way ANOVA of Gde1 cortex

Table Analyzed	Gde1 cortex				
Two-way ANOVA	Ordinary				
Alpha	0,05				
Source of Variation	% of total variation	P value	P value summary	Significant?	
Interaction	35,19	<0.0001	****	Yes	
Injury	27,25	<0.0001	****	Yes	
Genotype	43,36	<0.0001	****	Yes	
ANOVA table	SS (Type III)	DF	MS	F (DFn, DFd)	P value
Interaction	24,81	1	24,81	F (1, 16) = 125.2	P<0.0001
Injury	19,21	1	19,21	F (1, 16) = 96.94	P<0.0001
Genotype	30,57	1	30,57	F (1, 16) = 154.2	P<0.0001
Residual	3,171	16	0,1982		
Difference between column means					
Predicted (LS) mean of WT	0,8636				
Predicted (LS) mean of APP-SWE	3,387				
Difference between predicted means	-2,524				
SE of difference	0,2032				
95% CI of difference	-2.954 to -2.093				
Difference between row means					
Predicted (LS) mean of Sham	1,125				
Predicted (LS) mean of mTBI	3,126				
Difference between predicted means	-2,001				
SE of difference	0,2032				
95% CI of difference	-2.432 to -1.570				
Interaction CI					
Mean diff, A1 - B1	-0,2502				
Mean diff, A2 - B2	-4,797				
(A1 -B1) - (A2 - B2)	4,547				
95% CI of difference	3.685 to 5.409				
(B1 - A1) - (B2 - A2)	-4,547				
95% CI of difference	-5.409 to -3.685				
Normality of Residuals					
Test name	Statistics	P value	Passed normality test (alpha=0.05)?	P value summary	
D'Agostino-Pearson omnibus (K2)	1,533	0,4645	Yes	ns	
Anderson-Darling (A2*)	0,4298	0,2786	Yes	ns	
Shapiro-Wilk (W)	0,9350	0,1928	Yes	ns	
Kolmogorov-Smirnov (distance)	0,1782	0,0959	Yes	ns	
Data summary					
Number of columns (Genotype)	2				
Number of rows (Injury)	2				

**Number of values**	20				

2way ANOVA of Faah cortex

Table Analyzed	Faah cortex				
**Two-way ANOVA**	Ordinary				
**Alpha**	0,05				
**Source of Variation**	% of total variation	P value	P value summary	Significant?	
**Interaction**	12,25	0,1362	ns	No	
**Injury**	8,710	0,2045	ns	No	
**Genotype**	2,832	0,4616	ns	No	
**ANOVA table**	SS (Type III)	DF	MS	F (DFn, DFd)	P value
**Interaction**	0,4663	1	0,4663	F (1, 16) = 2.462	P=0.1362
**Injury**	0,3315	1	0,3315	F (1, 16) = 1.750	P=0.2045
**Genotype**	0,1078	1	0,1078	F (1, 16) = 0.5691	P=0.4616
**Residual**	3,031	16	0,1894		
**Difference between column means**					
**Predicted (LS) mean of WT**	0,9756				
**Predicted (LS) mean of APP-SWE**	0,8257				
**Difference between predicted means**	0,1499				
**SE of difference**	0,1987				
**95% CI of difference**	-0.2713 to 0.5710				
**Difference between row means**					
**Predicted (LS) mean of Sham**	0,7692				
**Predicted (LS) mean of mTBI**	1,032				
**Difference between predicted means**	-0,2628				
**SE of difference**	0,1987				
**95% CI of difference**	-0.6839 to 0.1583				
**Interaction CI**					
**Mean diff, A1 - B1**	0,4615				
**Mean diff, A2 - B2**	-0,1618				
**(A1 -B1) - (A2 - B2)**	0,6234				
**95% CI of difference**	-0.2189 to 1.466				
**(B1 - A1) - (B2 - A2)**	-0,6234				
**95% CI of difference**	-1.466 to 0.2189				
**Normality of Residuals**					
**Test name**	Statistics	P value	Passed normality test (alpha=0.05)?	P value summary	
**D'Agostino-Pearson omnibus (K2)**	5,369	0,0683	Yes	ns	
**Anderson-Darling (A2*)**	0,6148	0,0944	Yes	ns	
**Shapiro-Wilk (W)**	0,8996	0,0406	No	*	
**Kolmogorov-Smirnov (distance)**	0,1602	0,1000	Yes	ns	
**Data summary**					
**Number of columns (Genotype)**	2				
**Number of rows (Injury)**	2				

**Number of values**	20				

2way ANOVA of Dagla cortex

Table Analyzed	Dagla cortex				
Two-way ANOVA	Ordinary				
Alpha	0,05				
Source of Variation	% of total variation	P value	P value summary	Significant?	
Interaction	2,434	<0.0001	****	Yes	
Injury	2,195	<0.0001	****	Yes	
Genotype	95,00	<0.0001	****	Yes	
ANOVA table	SS (Type III)	DF	MS	F (DFn, DFd)	P value
Interaction	21,14	1	21,14	F (1, 16) = 34.14	P<0.0001
Injury	19,07	1	19,07	F (1, 16) = 30.80	P<0.0001
Genotype	825,1	1	825,1	F (1, 16) = 1333	P<0.0001
Residual	9,905	16	0,6190		
Difference between column means					
Predicted (LS) mean of WT	0,9473				
Predicted (LS) mean of APP-SWE	14,06				
Difference between predicted means	-13,11				
SE of difference	0,3591				
95% CI of difference	-13.87 to -12.35				
Difference between row means					
Predicted (LS) mean of Sham	6,506				
Predicted (LS) mean of mTBI	8,499				
Difference between predicted means	-1,993				
SE of difference	0,3591				
95% CI of difference	-2.754 to -1.232				
Interaction CI					
Mean diff, A1 - B1	-11,01				
Mean diff, A2 - B2	-15,21				
(A1 -B1) - (A2 - B2)	4,197				
95% CI of difference	2.674 to 5.720				
(B1 - A1) - (B2 - A2)	-4,197				
95% CI of difference	-5.720 to -2.674				
Normality of Residuals					
Test name	Statistics	P value	Passed normality test (alpha=0.05)?	P value summary	
D'Agostino-Pearson omnibus (K2)	6,119	0,0469	No	*	
Anderson-Darling (A2*)	0,6032	0,1012	Yes	ns	
Shapiro-Wilk (W)	0,9119	0,0694	Yes	ns	
Kolmogorov-Smirnov (distance)	0,1615	0,1000	Yes	ns	
Data summary					
Number of columns (Genotype)	2				
Number of rows (Injury)	2				

**Number of values**	20				

2way ANOVA of Magl cortex

Table Analyzed	Magl cortex				
Two-way ANOVA	Ordinary				
Alpha	0,05				
Source of Variation	% of total variation	P value	P value summary	Significant?	
Interaction	0,1597	0,8188	ns	No	
Injury	4,694	0,2249	ns	No	
Genotype	47,46	0,0010	**	Yes	
ANOVA table	SS (Type III)	DF	MS	F (DFn, DFd)	P value
Interaction	0,008658	1	0,008658	F (1, 16) = 0.05423	P=0.8188
Injury	0,2544	1	0,2544	F (1, 16) = 1.594	P=0.2249
Genotype	2,573	1	2,573	F (1, 16) = 16.12	P=0.0010
Residual	2,555	16	0,1597		
Difference between column means					
Predicted (LS) mean of WT	0,8636				
Predicted (LS) mean of APP-SWE	0,1315				
Difference between predicted means	0,7321				
SE of difference	0,1824				
95% CI of difference	0.3455 to 1.119				
Difference between row means					
Predicted (LS) mean of Sham	0,6127				
Predicted (LS) mean of mTBI	0,3825				
Difference between predicted means	0,2302				
SE of difference	0,1824				
95% CI of difference	-0.1564 to 0.6169				
Interaction CI					
Mean diff, A1 - B1	0,7746				
Mean diff, A2 - B2	0,6897				
(A1 -B1) - (A2 - B2)	0,08494				
95% CI of difference	-0.6883 to 0.8582				
(B1 - A1) - (B2 - A2)	-0,08494				
95% CI of difference	-0.8582 to 0.6883				
Normality of Residuals					
Test name	Statistics	P value	Passed normality test (alpha=0.05)?	P value summary	
D'Agostino-Pearson omnibus (K2)	3,295	0,1926	Yes	ns	
Anderson-Darling (A2*)	0,5824	0,1145	Yes	ns	
Shapiro-Wilk (W)	0,9167	0,0857	Yes	ns	
Kolmogorov-Smirnov (distance)	0,1859	0,0685	Yes	ns	
Data summary					
Number of columns (Genotype)	2				
Number of rows (Injury)	2				
Number of values	20				

2way ANOVA of Cb1 cortex

Table Analyzed	Cb1 cortex				
Two-way ANOVA	Ordinary				
Alpha	0,05				
Source of Variation	% of total variation	P value	P value summary	Significant?	
Interaction	3,506	<0.0001	****	Yes	
Injury	4,554	<0.0001	****	Yes	
Genotype	92,18	<0.0001	****	Yes	
ANOVA table	SS (Type III)	DF	MS	F (DFn, DFd)	P value
Interaction	5,278	1	5,278	F (1, 16) = 51.32	P<0.0001
Injury	6,856	1	6,856	F (1, 16) = 66.67	P<0.0001
Genotype	138,8	1	138,8	F (1, 16) = 1349	P<0.0001
Residual	1,645	16	0,1028		
Difference between column means					
Predicted (LS) mean of WT	1,073				
Predicted (LS) mean of APP-SWE	6,450				
Difference between predicted means	-5,377				
SE of difference	0,1464				
95% CI of difference	-5.687 to -5.066				
Difference between row means					
Predicted (LS) mean of Sham	3,164				
Predicted (LS) mean of mTBI	4,359				
Difference between predicted means	-1,195				
SE of difference	0,1464				
95% CI of difference	-1.505 to -0.8848				
Interaction CI					
Mean diff, A1 - B1	-4,328				
Mean diff, A2 - B2	-6,425				
(A1 -B1) - (A2 - B2)	2,097				
95% CI of difference	1.477 to 2.718				
(B1 - A1) - (B2 - A2)	-2,097				
95% CI of difference	-2.718 to -1.477				
Normality of Residuals					
Test name	Statistics	P value	Passed normality test (alpha=0.05)?	P value summary	
D'Agostino-Pearson omnibus (K2)	0,2610	0,8777	Yes	ns	
Anderson-Darling (A2*)	0,1341	0,9744	Yes	ns	
Shapiro-Wilk (W)	0,9844	0,9774	Yes	ns	
Kolmogorov-Smirnov (distance)	0,09623	0,1000	Yes	ns	
Data summary					
Number of columns (Genotype)	2				
Number of rows (Injury)	2				

**Number of values**	20				

2way ANOVA of Ppara cortex

Table Analyzed	Ppara cortex				
Two-way ANOVA	Ordinary				
Alpha	0,05				
Source of Variation	% of total variation	P value	P value summary	Significant?	
Interaction	8,825	0,1762	ns	No	
Injury	14,12	0,0924	ns	No	
Genotype	0,9406	0,6503	ns	No	
ANOVA table	SS (Type III)	DF	MS	F (DFn, DFd)	P value
Interaction	1,556	1	1,556	F (1, 16) = 2.003	P=0.1762
Injury	2,489	1	2,489	F (1, 16) = 3.204	P=0.0924
Genotype	0,1658	1	0,1658	F (1, 16) = 0.2134	P=0.6503
Residual	12,43	16	0,7770		
Difference between column means					
Predicted (LS) mean of WT	1,645				
Predicted (LS) mean of APP-SWE	1,459				
Difference between predicted means	0,1859				
SE of difference	0,4023				
95% CI of difference	-0.6670 to 1.039				
Difference between row means					
Predicted (LS) mean of Sham	1,192				
Predicted (LS) mean of mTBI	1,912				
Difference between predicted means	-0,7201				
SE of difference	0,4023				
95% CI of difference	-1.573 to 0.1328				
Interaction CI					
Mean diff, A1 - B1	-0,3835				
Mean diff, A2 - B2	0,7552				
(A1 -B1) - (A2 - B2)	-1,139				
95% CI of difference	-2.845 to 0.5671				
(B1 - A1) - (B2 - A2)	1,139				
95% CI of difference	-0.5671 to 2.845				
Normality of Residuals					
Test name	Statistics	P value	Passed normality test (alpha=0.05)?	P value summary	
D'Agostino-Pearson omnibus (K2)	19,78	<0.0001	No	****	
Anderson-Darling (A2*)	1,100	0,0053	No	**	
Shapiro-Wilk (W)	0,8246	0,0021	No	**	
Kolmogorov-Smirnov (distance)	0,2176	0,0139	No	*	
Data summary					
Number of columns (Genotype)	2				
Number of rows (Injury)	2				

**Number of values**	20				

2way ANOVA of Pparg cortex

Table Analyzed	Pparg cortex				
Two-way ANOVA	Ordinary				
Alpha	0,05				
Source of Variation	% of total variation	P value	P value summary	Significant?	
Interaction	0,4335	0,1061	ns	No	
Injury	0,7067	0,0439	*	Yes	
Genotype	96,22	<0.0001	****	Yes	
ANOVA table	SS (Type III)	DF	MS	F (DFn, DFd)	P value
Interaction	0,9913	1	0,9913	F (1, 16) = 2.934	P=0.1061
Injury	1,616	1	1,616	F (1, 16) = 4.783	P=0.0439
Genotype	220,0	1	220,0	F (1, 16) = 651.2	P<0.0001
Residual	5,406	16	0,3379		
Difference between column means					
Predicted (LS) mean of WT	1,517				
Predicted (LS) mean of APP-SWE	8,288				
Difference between predicted means	-6,770				
SE of difference	0,2653				
95% CI of difference	-7.333 to -6.208				
Difference between row means					
Predicted (LS) mean of Sham	4,612				
Predicted (LS) mean of mTBI	5,193				
Difference between predicted means	-0,5802				
SE of difference	0,2653				
95% CI of difference	-1.143 to -0.01778				
Interaction CI					
Mean diff, A1 - B1	-7,225				
Mean diff, A2 - B2	-6,316				
(A1 -B1) - (A2 - B2)	-0,9089				
95% CI of difference	-2.034 to 0.2160				
(B1 - A1) - (B2 - A2)	0,9089				
95% CI of difference	-0.2160 to 2.034				
Normality of Residuals					
Test name	Statistics	P value	Passed normality test (alpha=0.05)?	P value summary	
D'Agostino-Pearson omnibus (K2)	1,309	0,5196	Yes	ns	
Anderson-Darling (A2*)	0,7500	0,0424	No	*	
Shapiro-Wilk (W)	0,9321	0,1692	Yes	ns	
Kolmogorov-Smirnov (distance)	0,1812	0,0841	Yes	ns	
Data summary					
Number of columns (Genotype)	2				
Number of rows (Injury)	2				

**Number of values**	20				

2way ANOVA of Napepld hipp

Table Analyzed	Napepld hipp				
**Two-way ANOVA**	Ordinary				
**Alpha**	0,05				
**Source of Variation**	% of total variation	P value	P value summary	Significant?	
**Interaction**	0,1377	0,8208	ns	No	
**Injury**	13,99	0,0379	*	Yes	
**Genotype**	55,04	0,0006	***	Yes	
**ANOVA table**	SS	DF	MS	F (DFn, DFd)	P value
**Interaction**	0,004567	1	0,004567	F (1, 12) = 0.05358	P=0.8208
**Injury**	0,4638	1	0,4638	F (1, 12) = 5.442	P=0.0379
**Genotype**	1,825	1	1,825	F (1, 12) = 21.41	P=0.0006
**Residual**	1,023	12	0,08524		
**Difference between column means**					
**Mean of WT**	0,8466				
**Mean of APP-SWE**	1,522				
**Difference between means**	-0,6755				
**SE of difference**	0,1460				
**95% CI of difference**	-0.9936 to -0.3575				
**Difference between row means**					
**Mean of Sham**	1,355				
**Mean of mTBI**	1,014				
**Difference between means**	0,3405				
**SE of difference**	0,1460				
**95% CI of difference**	0.02247 to 0.6586				
**Interaction CI**					
**Mean diff, A1 - B1**	-0,7093				
**Mean diff, A2 - B2**	-0,6417				
**(A1 -B1) - (A2 - B2)**	-0,06758				
**95% CI of difference**	-0.7037 to 0.5685				
**(B1 - A1) - (B2 - A2)**	0,06758				
**95% CI of difference**	-0.5685 to 0.7037				
**Normality of Residuals**					
**Test name**	Statistics	P value	Passed normality test (alpha=0.05)?	P value summary	
**D'Agostino-Pearson omnibus (K2)**	10,79	0,0045	No	**	
**Anderson-Darling (A2*)**	0,6631	0,0676	Yes	ns	
**Shapiro-Wilk (W)**	0,8681	0,0254	No	*	
**Kolmogorov-Smirnov (distance)**	0,2134	0,0498	No	*	
**Data summary**					

**Number of columns (Genotype)**	2				
**Number of rows (Injury)**	2				
**Number of values**	16				

2way ANOVA of Gde1 hipp

Table Analyzed	Gde1 hipp				
**Two-way ANOVA**	Ordinary				
**Alpha**	0,05				
**Source of Variation**	% of total variation	P value	P value summary	Significant?	
**Interaction**	1,771	0,2159	ns	No	
**Injury**	0,05964	0,8146	ns	No	
**Genotype**	85,72	<0.0001	****	Yes	
**ANOVA table**	SS	DF	MS	F (DFn, DFd)	P value
**Interaction**	0,06592	1	0,06592	F (1, 12) = 1.707	P=0.2159
**Injury**	0,002220	1	0,002220	F (1, 12) = 0.05747	P=0.8146
**Genotype**	3,191	1	3,191	F (1, 12) = 82.60	P<0.0001
**Residual**	0,4635	12	0,03863		
**Difference between column means**					
**Mean of WT**	0,9240				
**Mean of APP-SWE**	1,817				
**Difference between means**	-0,8931				
**SE of difference**	0,09827				
**95% CI of difference**	-1.107 to -0.6790				
**Difference between row means**					
**Mean of Sham**	1,382				
**Mean of mTBI**	1,359				
**Difference between means**	0,02356				
**SE of difference**	0,09827				
**95% CI of difference**	-0.1906 to 0.2377				
**Interaction CI**					
**Mean diff, A1 - B1**	-0,7647				
**Mean diff, A2 - B2**	-1,021				
**(A1 -B1) - (A2 - B2)**	0,2568				
**95% CI of difference**	-0.1715 to 0.6850				
**(B1 - A1) - (B2 - A2)**	-0,2568				
**95% CI of difference**	-0.6850 to 0.1715				
**Normality of Residuals**					
**Test name**	Statistics	P value	Passed normality test (alpha=0.05)?	P value summary	
**D'Agostino-Pearson omnibus (K2)**	1,874	0,3918	Yes	ns	
**Anderson-Darling (A2*)**	0,3199	0,5039	Yes	ns	
**Shapiro-Wilk (W)**	0,9477	0,4537	Yes	ns	
**Kolmogorov-Smirnov (distance)**	0,1508	0,1000	Yes	ns	
**Data summary**					
**Number of columns (Genotype)**	2				

**Number of rows (Injury)**	2				
**Number of values**	16				

2way ANOVA of Faah hipp

Table Analyzed	Faah hipp				
Two-way ANOVA	Ordinary				
Alpha	0,05				
Source of Variation	% of total variation	P value	P value summary	Significant?	
Interaction	7,260	0,1554	ns	No	
Injury	4,677	0,2471	ns	No	
Genotype	50,16	0,0018	**	Yes	
ANOVA table	SS	DF	MS	F (DFn, DFd)	P value
Interaction	0,1243	1	0,1243	F (1, 12) = 2.298	P=0.1554
Injury	0,08007	1	0,08007	F (1, 12) = 1.481	P=0.2471
Genotype	0,8586	1	0,8586	F (1, 12) = 15.88	P=0.0018
Residual	0,6489	12	0,05408		
Difference between column means					
Mean of WT	0,9826				
Mean of APP-SWE	1,446				
Difference between means	-0,4633				
SE of difference	0,1163				
95% CI of difference	-0.7166 to -0.2100				
Difference between row means					
Mean of Sham	1,144				
Mean of mTBI	1,285				
Difference between means	-0,1415				
SE of difference	0,1163				
95% CI of difference	-0.3948 to 0.1119				
Interaction CI					
Mean diff, A1 - B1	-0,2870				
Mean diff, A2 - B2	-0,6396				
(A1 -B1) - (A2 - B2)	0,3525				
95% CI of difference	-0.1541 to 0.8592				
(B1 - A1) - (B2 - A2)	-0,3525				
95% CI of difference	-0.8592 to 0.1541				
Normality of Residuals					
Test name	Statistics	P value	Passed normality test (alpha=0.05)?	P value summary	
D'Agostino-Pearson omnibus (K2)	8,786	0,0124	No	*	
Anderson-Darling (A2*)	0,8020	0,0294	No	*	
Shapiro-Wilk (W)	0,8714	0,0285	No	*	
Kolmogorov-Smirnov (distance)	0,2111	0,0550	Yes	ns	
Data summary					

**Number of columns (Genotype)**	2				
**Number of rows (Injury)**	2				
**Number of values**	16				

2way ANOVA of Dagla hipp

Table Analyzed	Dagla hipp				
Two-way ANOVA	Ordinary				
Alpha	0,05				
Source of Variation	% of total variation	P value	P value summary	Significant?	
Interaction	0,2469	0,7057	ns	No	
Injury	5,343	0,0972	ns	No	
Genotype	74,60	<0.0001	****	Yes	
ANOVA table	SS	DF	MS	F (DFn, DFd)	P value
Interaction	0,01094	1	0,01094	F (1, 12) = 0.1496	P=0.7057
Injury	0,2368	1	0,2368	F (1, 12) = 3.237	P=0.0972
Genotype	3,306	1	3,306	F (1, 12) = 45.19	P<0.0001
Residual	0,8780	12	0,07317		
Difference between column means					
Mean of WT	0,9045				
Mean of APP-SWE	1,814				
Difference between means	-0,9092				
SE of difference	0,1352				
95% CI of difference	-1.204 to -0.6145				
Difference between row means					
Mean of Sham	1,481				
Mean of mTBI	1,237				
Difference between means	0,2433				
SE of difference	0,1352				
95% CI of difference	-0.05136 to 0.5380				
Interaction CI					
Mean diff, A1 - B1	-0,9615				
Mean diff, A2 - B2	-0,8569				
(A1 -B1) - (A2 - B2)	-0,1046				
95% CI of difference	-0.6940 to 0.4847				
(B1 - A1) - (B2 - A2)	0,1046				
95% CI of difference	-0.4847 to 0.6940				
Normality of Residuals					
Test name	Statistics	P value	Passed normality test (alpha=0.05)?	P value summary	
D'Agostino-Pearson omnibus (K2)	1,250	0,5354	Yes	ns	
Anderson-Darling (A2*)	0,2495	0,6999	Yes	ns	
Shapiro-Wilk (W)	0,9758	0,9214	Yes	ns	
Kolmogorov-Smirnov (distance)	0,1239	0,1000	Yes	ns	
Data summary					

**Number of columns (Genotype)**	2				
**Number of rows (Injury)**	2				
**Number of values**	16				

2way ANOVA of Magl hipp

Table Analyzed	Magl hipp				
Two-way ANOVA	Ordinary				
Alpha	0,05				
Source of Variation	% of total variation	P value	P value summary	Significant?	
Interaction	0,4712	0,7848	ns	No	
Injury	9,465	0,2346	ns	No	
Genotype	17,53	0,1143	ns	No	
ANOVA table	SS	DF	MS	F (DFn, DFd)	P value
Interaction	0,07526	1	0,07526	F (1, 12) = 0.07797	P=0.7848
Injury	1,512	1	1,512	F (1, 12) = 1.566	P=0.2346
Genotype	2,800	1	2,800	F (1, 12) = 2.901	P=0.1143
Residual	11,58	12	0,9653		
Difference between column means					
Mean of WT	0,7612				
Mean of APP-SWE	1,598				
Difference between means	-0,8367				
SE of difference	0,4913				
95% CI of difference	-1.907 to 0.2336				
Difference between row means					
Mean of Sham	1,487				
Mean of mTBI	0,8722				
Difference between means	0,6147				
SE of difference	0,4913				
95% CI of difference	-0.4556 to 1.685				
Interaction CI					
Mean diff, A1 - B1	-0,9739				
Mean diff, A2 - B2	-0,6995				
(A1 -B1) - (A2 - B2)	-0,2743				
95% CI of difference	-2.415 to 1.866				
(B1 - A1) - (B2 - A2)	0,2743				
95% CI of difference	-1.866 to 2.415				
Normality of Residuals					
Test name	Statistics	P value	Passed normality test (alpha=0.05)?	P value summary	
D'Agostino-Pearson omnibus (K2)	6,711	0,0349	No	*	
Anderson-Darling (A2*)	0,5627	0,1211	Yes	ns	
Shapiro-Wilk (W)	0,9214	0,1779	Yes	ns	
Kolmogorov-Smirnov (distance)	0,1835	0,1000	Yes	ns	
Data summary					

**Number of columns (Genotype)**	2				
**Number of rows (Injury)**	2				
**Number of values**	16				

2way ANOVA of Cb1 hipp

Table Analyzed	Cb1 hipp				
**Two-way ANOVA**	Ordinary				
**Alpha**	0,05				
**Source of Variation**	% of total variation	P value	P value summary	Significant?	
**Interaction**	4,508	0,2359	ns	No	
**Injury**	15,35	0,0400	*	Yes	
**Genotype**	45,40	0,0019	**	Yes	
**ANOVA table**	SS	DF	MS	F (DFn, DFd)	P value
**Interaction**	0,03596	1	0,03596	F (1, 12) = 1.557	P=0.2359
**Injury**	0,1225	1	0,1225	F (1, 12) = 5.303	P=0.0400
**Genotype**	0,3622	1	0,3622	F (1, 12) = 15.68	P=0.0019
**Residual**	0,2771	12	0,02309		
**Difference between column means**					
**Mean of WT**	0,8651				
**Mean of APP-SWE**	1,166				
**Difference between means**	-0,3009				
**SE of difference**	0,07598				
**95% CI of difference**	-0.4665 to -0.1354				
**Difference between row means**					
**Mean of Sham**	1,103				
**Mean of mTBI**	0,9281				
**Difference between means**	0,1750				
**SE of difference**	0,07598				
**95% CI of difference**	0.009419 to 0.3405				
**Interaction CI**					
**Mean diff, A1 - B1**	-0,2061				
**Mean diff, A2 - B2**	-0,3957				
**(A1 -B1) - (A2 - B2)**	0,1896				
**95% CI of difference**	-0.1415 to 0.5207				
**(B1 - A1) - (B2 - A2)**	-0,1896				
**95% CI of difference**	-0.5207 to 0.1415				
**Normality of Residuals**					
**Test name**	Statistics	P value	Passed normality test (alpha=0.05)?	P value summary	
**D'Agostino-Pearson omnibus (K2)**	2,271	0,3212	Yes	ns	
**Anderson-Darling (A2*)**	0,2881	0,5711	Yes	ns	
**Shapiro-Wilk (W)**	0,9509	0,5042	Yes	ns	
**Kolmogorov-Smirnov (distance)**	0,1363	0,1000	Yes	ns	
**Data summary**					

**Number of columns (Genotype)**	2				
**Number of rows (Injury)**	2				
**Number of values**	16				

2way ANOVA of Ppara hipp

Table Analyzed	Ppara hipp				
**Two-way ANOVA**	Ordinary				
**Alpha**	0,05				
**Source of Variation**	% of total variation	P value	P value summary	Significant?	
**Interaction**	20,73	0,0037	**	Yes	
**Injury**	2,152	0,2703	ns	No	
**Genotype**	57,79	<0.0001	****	Yes	
**ANOVA table**	SS	DF	MS	F (DFn, DFd)	P value
**Interaction**	0,3246	1	0,3246	F (1, 12) = 12.86	P=0.0037
**Injury**	0,03370	1	0,03370	F (1, 12) = 1.336	P=0.2703
**Genotype**	0,9050	1	0,9050	F (1, 12) = 35.87	P<0.0001
**Residual**	0,3028	12	0,02523		
**Difference between column means**					
**Mean of WT**	0,8117				
**Mean of APP-SWE**	0,3360				
**Difference between means**	0,4757				
**SE of difference**	0,07942				
**95% CI of difference**	0.3026 to 0.6487				
**Difference between row means**					
**Mean of Sham**	0,6197				
**Mean of mTBI**	0,5280				
**Difference between means**	0,09179				
**SE of difference**	0,07942				
**95% CI of difference**	-0.08125 to 0.2648				
**Interaction CI**					
**Mean diff, A1 - B1**	0,7605				
**Mean diff, A2 - B2**	0,1908				
**(A1 -B1) - (A2 - B2)**	0,5697				
**95% CI of difference**	0.2236 to 0.9158				
**(B1 - A1) - (B2 - A2)**	-0,5697				
**95% CI of difference**	-0.9158 to -0.2236				
**Normality of Residuals**					
**Test name**	Statistics	P value	Passed normality test (alpha=0.05)?	P value summary	
**D'Agostino-Pearson omnibus (K2)**	0,6883	0,7088	Yes	ns	
**Anderson-Darling (A2*)**	0,1990	0,8610	Yes	ns	
**Shapiro-Wilk (W)**	0,9820	0,9775	Yes	ns	
**Kolmogorov-Smirnov (distance)**	0,1107	0,1000	Yes	ns	
**Data summary**					

**Number of columns (Genotype)**	2				
**Number of rows (Injury)**	2				
**Number of values**	16				

2way ANOVA of Pparg hipp

Table Analyzed	Pparg hipp				
**Two-way ANOVA**	Ordinary				
**Alpha**	0,05				
**Source of Variation**	% of total variation	P value	P value summary	Significant?	
**Interaction**	8,959	<0.0001	****	Yes	
**Injury**	3,234	0,0030	**	Yes	
**Genotype**	84,99	<0.0001	****	Yes	
**ANOVA table**	SS	DF	MS	F (DFn, DFd)	P value
**Interaction**	2,061	1	2,061	F (1, 12) = 38.19	P<0.0001
**Injury**	0,7442	1	0,7442	F (1, 12) = 13.79	P=0.0030
**Genotype**	19,56	1	19,56	F (1, 12) = 362.3	P<0.0001
**Residual**	0,6478	12	0,05398		
**Difference between column means**					
**Mean of WT**	0,8567				
**Mean of APP-SWE**	3,068				
**Difference between means**	-2,211				
**SE of difference**	0,1162				
**95% CI of difference**	-2.464 to -1.958				
**Difference between row means**					
**Mean of Sham**	1,747				
**Mean of mTBI**	2,178				
**Difference between means**	-0,4313				
**SE of difference**	0,1162				
**95% CI of difference**	-0.6844 to -0.1782				
**Interaction CI**					
**Mean diff, A1 - B1**	-1,493				
**Mean diff, A2 - B2**	-2,929				
**(A1 -B1) - (A2 - B2)**	1,436				
**95% CI of difference**	0.9295 to 1.942				
**(B1 - A1) - (B2 - A2)**	-1,436				
**95% CI of difference**	-1.942 to -0.9295				
**Normality of Residuals**					
**Test name**	Statistics	P value	Passed normality test (alpha=0.05)?	P value summary	
**D'Agostino-Pearson omnibus (K2)**	2,233	0,3274	Yes	ns	
**Anderson-Darling (A2*)**	0,4595	0,2270	Yes	ns	
**Shapiro-Wilk (W)**	0,9378	0,3224	Yes	ns	
**Kolmogorov-Smirnov (distance)**	0,1669	0,1000	Yes	ns	
**Data summary**					

**Number of columns (Genotype)**	2				
**Number of rows (Injury)**	2				
**Number of values**	16				

2way ANOVA of 5HT cortex

Table Analyzed	5HT cortex				
**Two-way ANOVA**	Ordinary				
**Alpha**	0,05				
**Source of Variation**	% of total variation	P value	P value summary	Significant?	
**Interaction**	0,2273	0,7059	ns	No	
**Injury**	51,79	0,0004	***	Yes	
**Genotype**	36,10	0,0012	**	Yes	
**ANOVA table**	SS	DF	MS	F (DFn, DFd)	P value
**Interaction**	21194834	1	21194834	F (1, 8) = 0.1530	P=0.7059
**Injury**	4830254644	1	4830254644	F (1, 8) = 34.86	P=0.0004
**Genotype**	3366372064	1	3366372064	F (1, 8) = 24.30	P=0.0012
**Residual**	1108371191	8	138546399		
**Difference between column means**					
**Mean of WT**	98341				
**Mean of APP-SWE**	64842				
**Difference between means**	33498				
**SE of difference**	6796				
**95% CI of difference**	17827 to 49169				
**Difference between row means**					
**Mean of Sham**	101654				
**Mean of mTBI**	61529				
**Difference between means**	40126				
**SE of difference**	6796				
**95% CI of difference**	24455 to 55797				
**Interaction CI**					
**Mean diff, A1 - B1**	30840				
**Mean diff, A2 - B2**	36156				
**(A1 -B1) - (A2 - B2)**	-5316				
**95% CI of difference**	-36658 to 26026				
**(B1 - A1) - (B2 - A2)**	5316				
**95% CI of difference**	-26026 to 36658				
**Normality of Residuals**					
**Test name**	Statistics	P value	Passed normality test (alpha=0.05)?	P value summary	
**D'Agostino-Pearson omnibus (K2)**	2,403	0,3008	Yes	ns	
**Anderson-Darling (A2*)**	0,3764	0,3513	Yes	ns	
**Shapiro-Wilk (W)**	0,9222	0,3044	Yes	ns	
**Kolmogorov-Smirnov (distance)**	0,1711	0,1000	Yes	ns	
**Data summary**					

**Number of columns (Genotype)**	2				
**Number of rows (Injury)**	2				
**Number of values**	12				

2way ANOVA of 5HT hipp

Table Analyzed	5HT hipp				
Two-way ANOVA	Ordinary				
Alpha	0,05				
Source of Variation	% of total variation	P value	P value summary	Significant?	
Interaction	12,70	0,0914	ns	No	
Injury	10,67	0,1168	ns	No	
Genotype	49,01	0,0055	**	Yes	
ANOVA table	SS	DF	MS	F (DFn, DFd)	P value
Interaction	2733073668	1	2733073668	F (1, 8) = 3.678	P=0.0914
Injury	2296419101	1	2296419101	F (1, 8) = 3.091	P=0.1168
Genotype	10546404413	1	10546404413	F (1, 8) = 14.19	P=0.0055
Residual	5944130395	8	743016299		
Difference between column means					
Mean of WT	116963				
Mean of APP-SWE	176254				
Difference between means	-59291				
SE of difference	15738				
95% CI of difference	-95582 to -23000				
Difference between row means					
Mean of Sham	160442				
Mean of mTBI	132775				
Difference between means	27667				
SE of difference	15738				
95% CI of difference	-8624 to 63958				
Interaction CI					
Mean diff, A1 - B1	-89475				
Mean diff, A2 - B2	-29108				
(A1 -B1) - (A2 - B2)	-60366				
95% CI of difference	-132948 to 12216				
(B1 - A1) - (B2 - A2)	60366				
95% CI of difference	-12216 to 132948				
Normality of Residuals					
Test name	Statistics	P value	Passed normality test (alpha=0.05)?	P value summary	
D'Agostino-Pearson omnibus (K2)	1,393	0,4982	Yes	ns	
Anderson-Darling (A2*)	0,2786	0,5815	Yes	ns	
Shapiro-Wilk (W)	0,9451	0,5673	Yes	ns	
Kolmogorov-Smirnov (distance)	0,1469	0,1000	Yes	ns	
Data summary					

**Number of columns (Genotype)**	2				
**Number of rows (Injury)**	2				
**Number of values**	12				

2way ANOVA of 5HIAA cortex

Compare cell means regardless of rows and columns								
Number of families	1							
Number of comparisons per family	6							
Alpha	0,05							
Tukey's multiple comparisons test	Mean Diff.	95.00% CI of diff.	Below threshold?	Summary	Adjusted P Value			
Sham:WT vs. Sham:APP-SWE	2718	-1751 to 7187	No	ns	0,2824			
Sham:WT vs. mTBI:WT	760,8	-3708 to 5230	No	ns	0,9453			
Sham:WT vs. mTBI:APP-SWE	7200	2731 to 11669	Yes	**	0,0038			
Sham:APP-SWE vs. mTBI:WT	-1957	-6427 to 2512	No	ns	0,5315			
Sham:APP-SWE vs. mTBI:APP-SWE	4481	12.29 to 8950	Yes	*	0,0494			
mTBI:WT vs. mTBI:APP-SWE	6439	1970 to 10908	Yes	**	0,0075			
Test details	Mean 1	Mean 2	Mean Diff.	SE of diff.	N1	N2	q	DF
Sham:WT vs. Sham:APP-SWE	12371	9653	2718	1396	3	3	2,755	8,000
Sham:WT vs. mTBI:WT	12371	11610	760,8	1396	3	3	0,7710	8,000
Sham:WT vs. mTBI:APP-SWE	12371	5171	7200	1396	3	3	7,296	8,000
Sham:APP-SWE vs. mTBI:WT	9653	11610	-1957	1396	3	3	1,984	8,000
Sham:APP-SWE vs. mTBI:APP-SWE	9653	5171	4481	1396	3	3	4,541	8,000
mTBI:WT vs. mTBI:APP-SWE	11610	5171	6439	1396	3	3	6,525	8,000

2way ANOVA of 5HIAA hipp

Compare cell means regardless of rows and columns								
**Number of families**	1							
**Number of comparisons per family**	6							
**Alpha**	0,05							
**Tukey's multiple comparisons test**	Mean Diff.	95.00% CI of diff.	Below threshold?	Summary	Adjusted P Value			
**Sham:WT vs. Sham:APP-SWE**	-15237	-23328 to -7146	Yes	**	0,0014			
**Sham:WT vs. mTBI:WT**	-9070	-17161 to -979.3	Yes	*	0,0291			
**Sham:WT vs. mTBI:APP-SWE**	-3921	-12012 to 4170	No	ns	0,4537			
**Sham:APP-SWE vs. mTBI:WT**	6167	-1924 to 14258	No	ns	0,1460			
**Sham:APP-SWE vs. mTBI:APP-SWE**	11316	3225 to 19407	Yes	**	0,0089			
**mTBI:WT vs. mTBI:APP-SWE**	5150	-2942 to 13241	No	ns	0,2513			
**Test details**	Mean 1	Mean 2	Mean Diff.	SE of diff.	N1	N2	q	DF
**Sham:WT vs. Sham:APP-SWE**	28276	43513	-15237	2527	3	3	8,529	8,000
**Sham:WT vs. mTBI:WT**	28276	37346	-9070	2527	3	3	5,077	8,000
**Sham:WT vs. mTBI:APP-SWE**	28276	32197	-3921	2527	3	3	2,195	8,000
**Sham:APP-SWE vs. mTBI:WT**	43513	37346	6167	2527	3	3	3,452	8,000
**Sham:APP-SWE vs. mTBI:APP-SWE**	43513	32197	11316	2527	3	3	6,334	8,000
**mTBI:WT vs. mTBI:APP-SWE**	37346	32197	5150	2527	3	3	2,882	8,000

2way ANOVA of OA5HT cortex MSI

Table Analyzed	OA5HT cortex MSI				
Two-way ANOVA	Ordinary				
Alpha	0,05				
Source of Variation	% of total variation	P value	P value summary	Significant?	
Interaction	7,126	0,0787	ns	No	
Injury	28,58	0,0038	**	Yes	
Genotype	50,26	0,0007	***	Yes	
ANOVA table	SS	DF	MS	F (DFn, DFd)	P value
Interaction	398747	1	398747	F (1, 8) = 4.061	P=0.0787
Injury	1598982	1	1598982	F (1, 8) = 16.28	P=0.0038
Genotype	2812067	1	2812067	F (1, 8) = 28.64	P=0.0007
Residual	785589	8	98199		
Difference between column means					
Mean of WT	1910				
Mean of APP-SWE	941,6				
Difference between means	968,2				
SE of difference	180,9				
95% CI of difference	551.0 to 1385				
Difference between row means					
Mean of Sham	1061				
Mean of mTBI	1791				
Difference between means	-730,1				
SE of difference	180,9				
95% CI of difference	-1147 to -312.9				
Interaction CI					
Mean diff, A1 - B1	603,6				
Mean diff, A2 - B2	1333				
(A1 -B1) - (A2 - B2)	-729,2				
95% CI of difference	-1564 to 105.3				
(B1 - A1) - (B2 - A2)	729,2				
95% CI of difference	-105.3 to 1564				
Normality of Residuals					
Test name	Statistics	P value	Passed normality test (alpha=0.05)?	P value summary	
D'Agostino-Pearson omnibus (K2)	0,06299	0,9690	Yes	ns	
Anderson-Darling (A2*)	0,1739	0,9034	Yes	ns	
Shapiro-Wilk (W)	0,9736	0,9448	Yes	ns	
Kolmogorov-Smirnov (distance)	0,1111	0,1000	Yes	ns	
Data summary					

**Number of columns (Genotype)**	2				
**Number of rows (Injury)**	2				
**Number of values**	12				

2way ANOVA of OA5HT hipp MSI

Table Analyzed	OA5HT hipp MSI				
Two-way ANOVA	Ordinary				
Alpha	0,05				
Source of Variation	% of total variation	P value	P value summary	Significant?	
Interaction	3,522	0,3953	ns	No	
Injury	0,8273	0,6748	ns	No	
Genotype	60,73	0,0058	**	Yes	
ANOVA table	SS	DF	MS	F (DFn, DFd)	P value
Interaction	62538	1	62538	F (1, 8) = 0.8068	P=0.3953
Injury	14690	1	14690	F (1, 8) = 0.1895	P=0.6748
Genotype	1078354	1	1078354	F (1, 8) = 13.91	P=0.0058
Residual	620144	8	77518		
Difference between column means					
Mean of WT	1762				
Mean of APP-SWE	1163				
Difference between means	599,5				
SE of difference	160,7				
95% CI of difference	228.9 to 970.2				
Difference between row means					
Mean of Sham	1497				
Mean of mTBI	1428				
Difference between means	69,98				
SE of difference	160,7				
95% CI of difference	-300.7 to 440.7				
Interaction CI					
Mean diff, A1 - B1	455,2				
Mean diff, A2 - B2	743,9				
(A1 -B1) - (A2 - B2)	-288,8				
95% CI of difference	-1030 to 452.6				
(B1 - A1) - (B2 - A2)	288,8				
95% CI of difference	-452.6 to 1030				
Normality of Residuals					
Test name	Statistics	P value	Passed normality test (alpha=0.05)?	P value summary	
D'Agostino-Pearson omnibus (K2)	2,457	0,2928	Yes	ns	
Anderson-Darling (A2*)	0,5893	0,0977	Yes	ns	
Shapiro-Wilk (W)	0,8684	0,0624	Yes	ns	
Kolmogorov-Smirnov (distance)	0,1512	0,1000	Yes	ns	
Data summary					

**Number of columns (Genotype)**	2				
**Number of rows (Injury)**	2				
**Number of values**	12				

2way ANOVA of Gene 5HT2a cortex

Table Analyzed	Gene 5HT2a cortex				
Two-way ANOVA	Ordinary				
Alpha	0,05				
Source of Variation	% of total variation	P value	P value summary	Significant?	
Interaction	13,14	0,0043	**	Yes	
Injury	41,68	0,0001	***	Yes	
Genotype	38,40	0,0001	***	Yes	
ANOVA table	SS	DF	MS	F (DFn, DFd)	P value
Interaction	0,08100	1	0,08100	F (1, 8) = 15.49	P=0.0043
Injury	0,2569	1	0,2569	F (1, 8) = 49.15	P=0.0001
Genotype	0,2367	1	0,2367	F (1, 8) = 45.28	P=0.0001
Residual	0,04182	8	0,005227		
Difference between column means					
Mean of WT	0,9358				
Mean of APP-SWE	1,217				
Difference between means	-0,2809				
SE of difference	0,04174				
95% CI of difference	-0.3772 to -0.1846				
Difference between row means					
Mean of Sham	1,223				
Mean of mTBI	0,9300				
Difference between means	0,2926				
SE of difference	0,04174				
95% CI of difference	0.1964 to 0.3889				
Interaction CI					
Mean diff, A1 - B1	-0,4452				
Mean diff, A2 - B2	-0,1166				
(A1 -B1) - (A2 - B2)	-0,3286				
95% CI of difference	-0.5211 to -0.1361				
(B1 - A1) - (B2 - A2)	0,3286				
95% CI of difference	0.1361 to 0.5211				
Normality of Residuals					
Test name	Statistics	P value	Passed normality test (alpha=0.05)?	P value summary	
D'Agostino-Pearson omnibus (K2)	2,032	0,3621	Yes	ns	
Anderson-Darling (A2*)	0,3245	0,4732	Yes	ns	
Shapiro-Wilk (W)	0,9307	0,3873	Yes	ns	
Kolmogorov-Smirnov (distance)	0,1690	0,1000	Yes	ns	
Data summary					

**Number of columns (Genotype)**	2				
**Number of rows (Injury)**	2				
**Number of values**	12				

2way ANOVA of Gene 5HT2a hipp

Compare cell means regardless of rows and columns								
**Number of families**	1							
**Number of comparisons per family**	6							
**Alpha**	0,05							
**Tukey's multiple comparisons test**	Predicted (LS) mean diff.	95.00% CI of diff.	Below threshold?	Summary	Adjusted P Value			
**Sham:WT vs. Sham:APP-SWE**	0,4285	-0.1428 to 0.9998	No	ns	0,1845			
**Sham:WT vs. mTBI:WT**	0,05278	-0.5917 to 0.6973	No	ns	0,9955			
**Sham:WT vs. mTBI:APP-SWE**	0,4716	-0.09973 to 1.043	No	ns	0,1275			
**Sham:APP-SWE vs. mTBI:WT**	-0,3757	-0.9470 to 0.1956	No	ns	0,2799			
**Sham:APP-SWE vs. mTBI:APP-SWE**	0,04307	-0.4441 to 0.5303	No	ns	0,9943			
**mTBI:WT vs. mTBI:APP-SWE**	0,4188	-0.1525 to 0.9901	No	ns	0,1999			
**Test details**	Predicted (LS) mean 1	Predicted (LS) mean 2	Predicted (LS) mean diff.	SE of diff.	N1	N2	q	DF
**Sham:WT vs. Sham:APP-SWE**	1,000	0,5715	0,4285	0,2021	4	7	2,998	18,00
**Sham:WT vs. mTBI:WT**	1,000	0,9472	0,05278	0,2280	4	4	0,3273	18,00
**Sham:WT vs. mTBI:APP-SWE**	1,000	0,5284	0,4716	0,2021	4	7	3,299	18,00
**Sham:APP-SWE vs. mTBI:WT**	0,5715	0,9472	-0,3757	0,2021	7	4	2,629	18,00
**Sham:APP-SWE vs. mTBI:APP-SWE**	0,5715	0,5284	0,04307	0,1724	7	7	0,3533	18,00
**mTBI:WT vs. mTBI:APP-SWE**	0,9472	0,5284	0,4188	0,2021	4	7	2,930	18,00

2way ANOVA of 5HT2a:CB1 cortex

Table Analyzed	5HT2a/CB1 cortex				
Two-way ANOVA	Ordinary				
Alpha	0,05				
Source of Variation	% of total variation	P value	P value summary	Significant?	
Interaction	38,99	<0.0001	****	Yes	
Injury	32,34	<0.0001	****	Yes	
Genotype	28,37	<0.0001	****	Yes	
ANOVA table	SS	DF	MS	F (DFn, DFd)	P value
Interaction	2,444	1	2,444	F (1, 4) = 505.4	P<0.0001
Injury	2,027	1	2,027	F (1, 4) = 419.3	P<0.0001
Genotype	1,778	1	1,778	F (1, 4) = 367.7	P<0.0001
Residual	0,01934	4	0,004835		
Difference between column means					
Mean of WT	0,9507				
Mean of APP-SWE	1,894				
Difference between means	-0,9429				
SE of difference	0,04917				
95% CI of difference	-1.079 to -0.8064				
Difference between row means					
Mean of Sham	0,9188				
Mean of mTBI	1,926				
Difference between means	-1,007				
SE of difference	0,04917				
95% CI of difference	-1.143 to -0.8703				
Interaction CI					
Mean diff, A1 - B1	0,1625				
Mean diff, A2 - B2	-2,048				
(A1 -B1) - (A2 - B2)	2,211				
95% CI of difference	1.938 to 2.484				
(B1 - A1) - (B2 - A2)	-2,211				
95% CI of difference	-2.484 to -1.938				
Normality of Residuals					
Test name	Statistics	P value	Passed normality test (alpha=0.05)?	P value summary	
D'Agostino-Pearson omnibus (K2)	0,3762	0,8285	Yes	ns	
Anderson-Darling (A2*)	0,3009	0,5018	Yes	ns	
Shapiro-Wilk (W)	0,9121	0,3693	Yes	ns	
Kolmogorov-Smirnov (distance)	0,1518	0,1000	Yes	ns	
Data summary					

**Number of columns (Genotype)**	2				
**Number of rows (Injury)**	2				
**Number of values**	8				
