# Supplementary figures and images for: Early biomarkers in the presymptomatic phase of cognitive impairment: changes in the endocannabinoidome and serotonergic pathways in Alzheimer's-prone mice after mTBI
Source: Acta Neuropathol Commun. 2024 Jul 12;12:113. doi: 10.1186/s40478-024-01820-0 (PMC11241935; doi:10.1186/s40478-024-01820-0)

**A**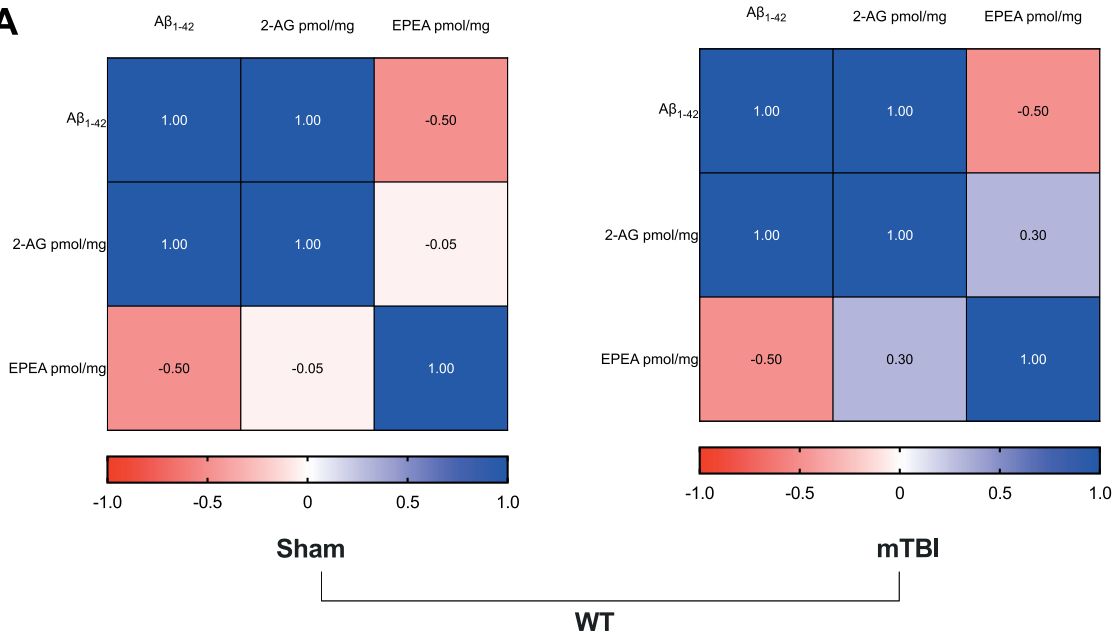**B**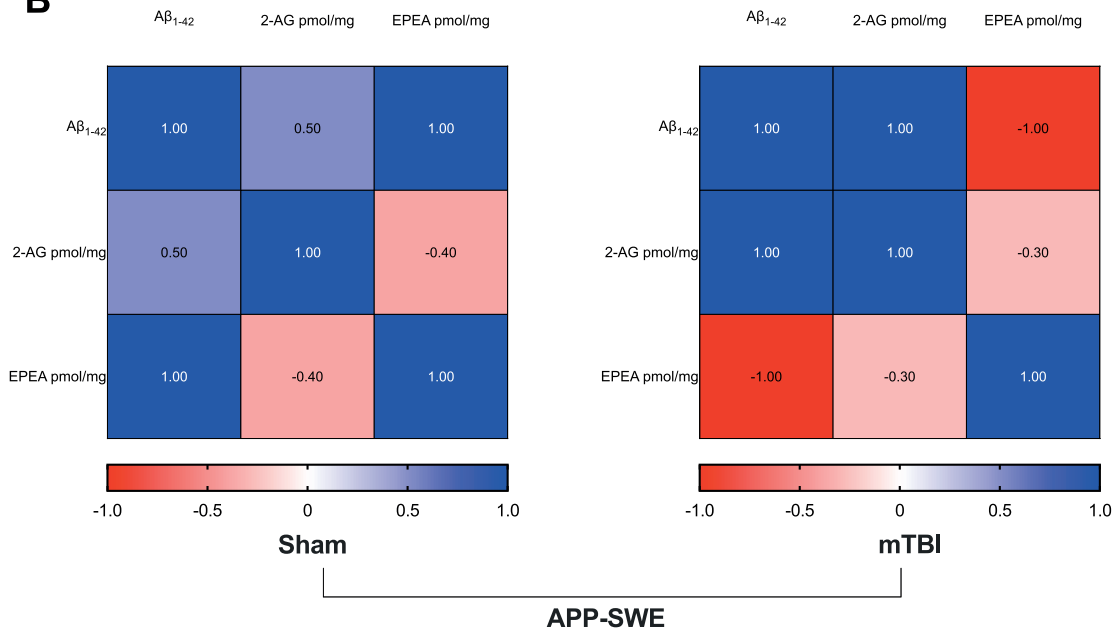

Supplement: Supplementary file 3 — Additional file 3. [file 40478_2024_1820_MOESM3_ESM.pdf]

**A**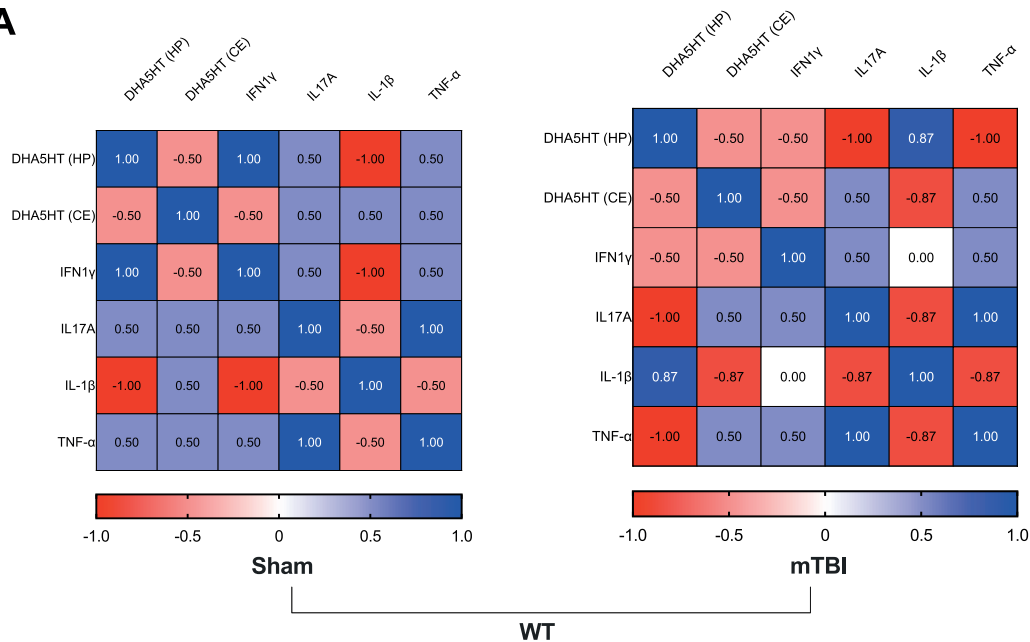**B**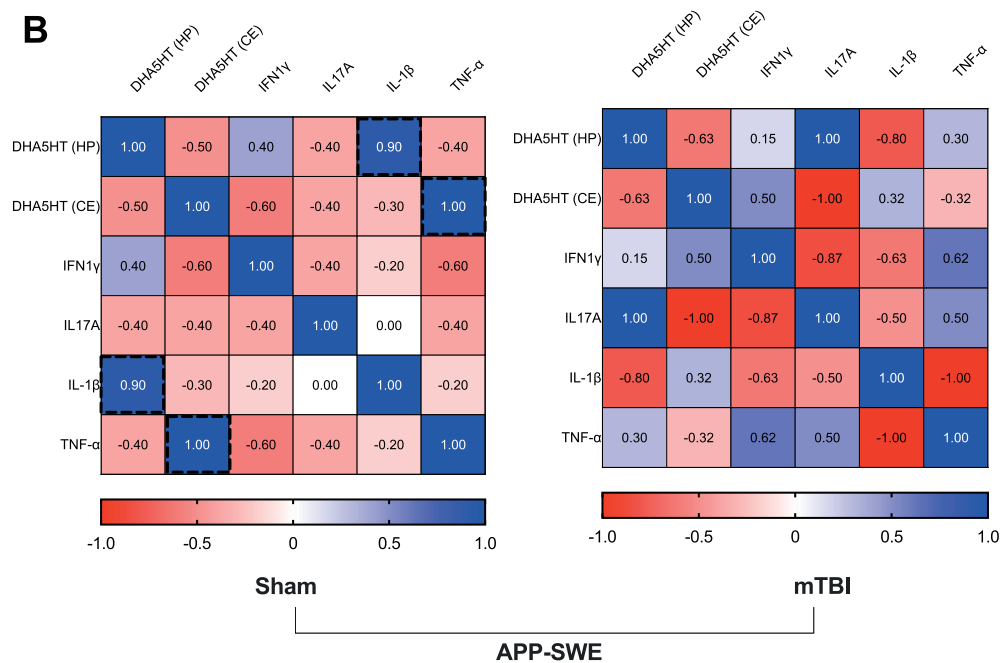

P=0.083

Supplement: Supplementary file 4 — Additional file 4. [file 40478_2024_1820_MOESM4_ESM.pdf]

A

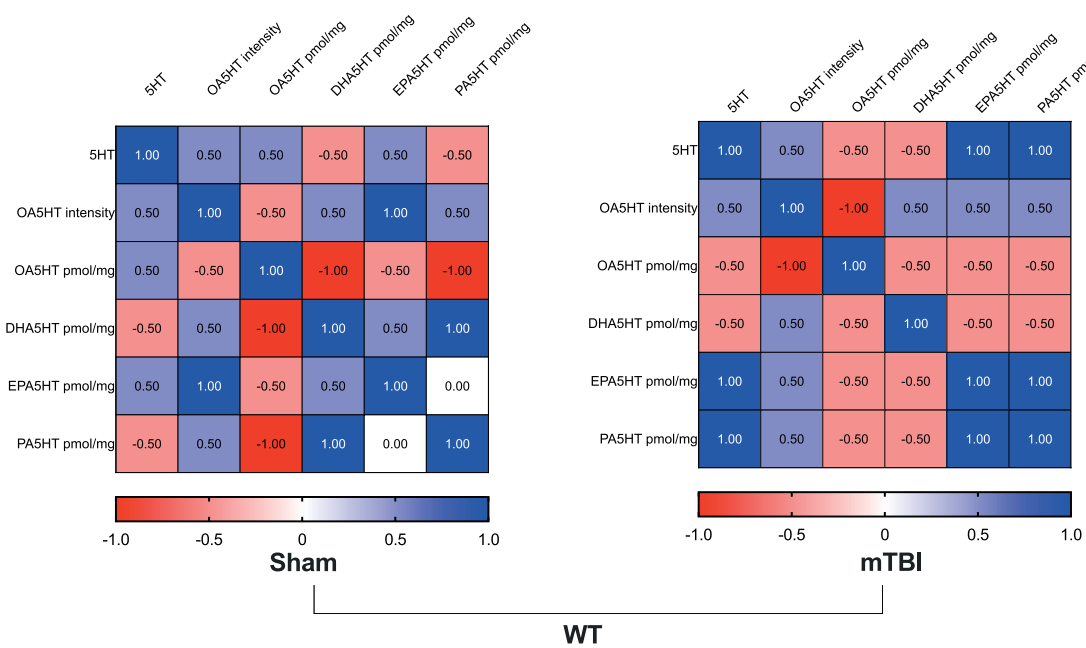

B

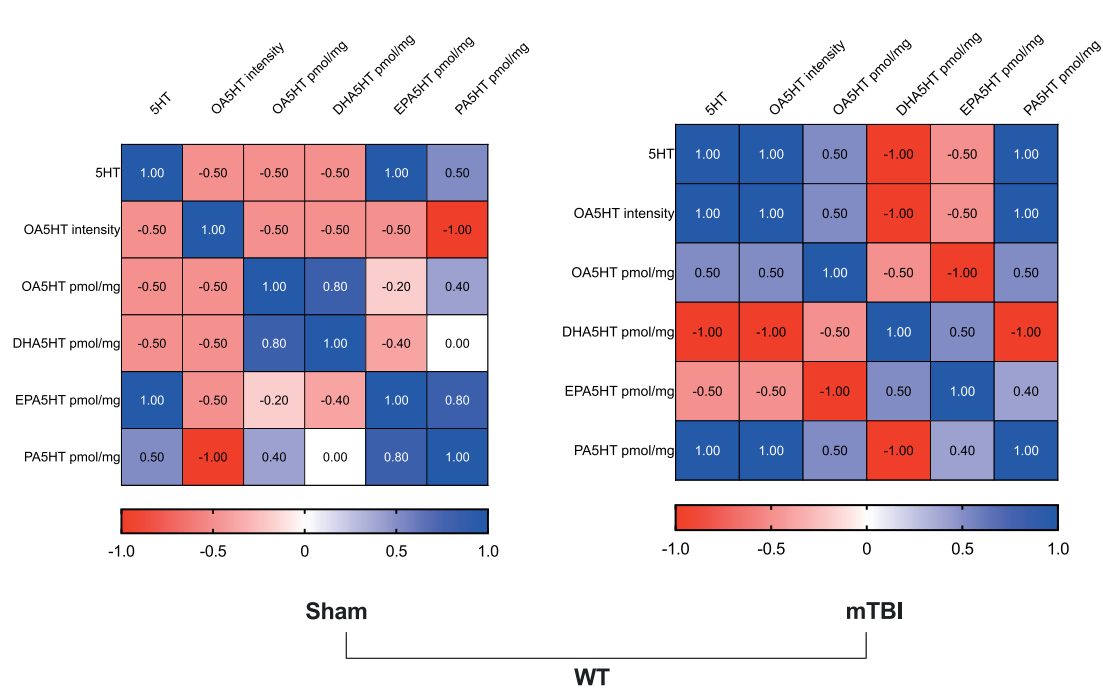

APP-SWE

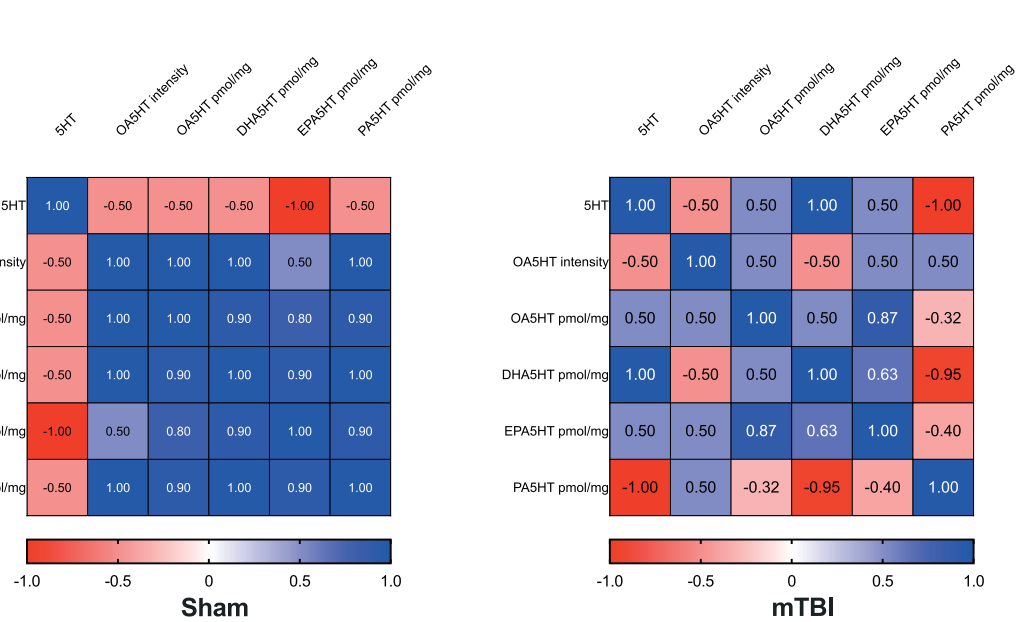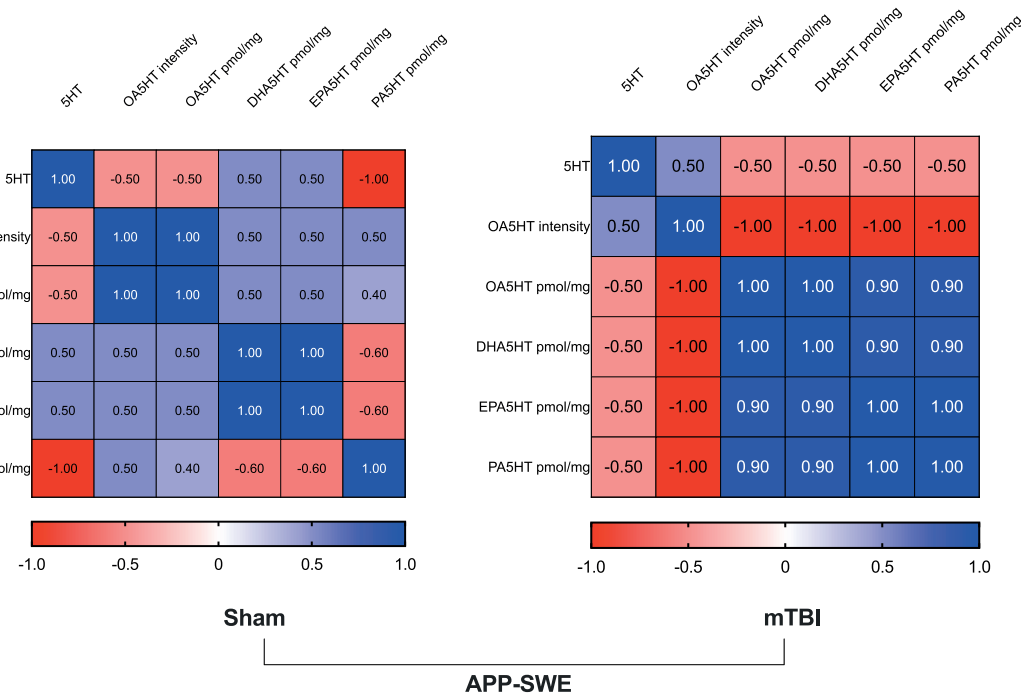

Supplement: Supplementary file 6 — Additional file 6. [file 40478_2024_1820_MOESM6_ESM.pdf]

**A**

wt sham      wt sham

75

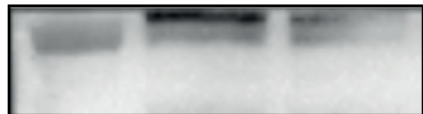**Input****B**

wt sham      wt TBI      APP sham      APP TBI

75

50

37

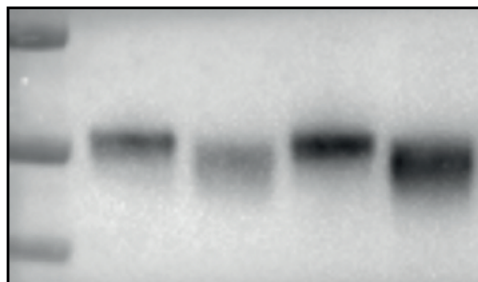**IgG**

Supplement: Supplementary file 7 — Additional file 7. [file 40478_2024_1820_MOESM7_ESM.pdf]
