# Supplementary material for: Early biomarkers in the presymptomatic phase of cognitive impairment: changes in the endocannabinoidome and serotonergic pathways in Alzheimer's-prone mice after mTBI
Source: Acta Neuropathol Commun. 2024 Jul 12;12:113. doi: 10.1186/s40478-024-01820-0 (PMC11241935; doi:10.1186/s40478-024-01820-0)
Supplement: Supplementary file 5 — Additional file 5. [file 40478_2024_1820_MOESM5_ESM.pdf]

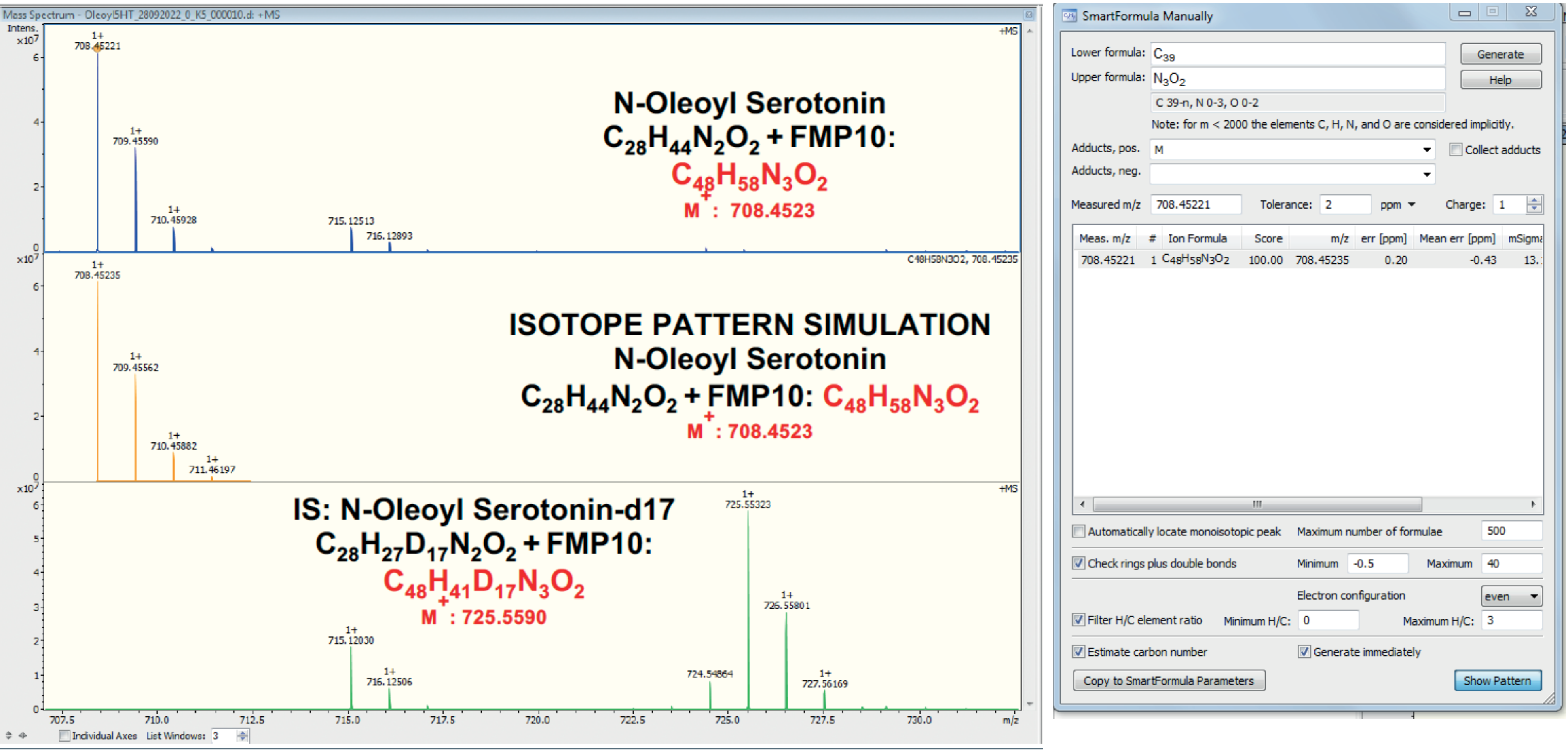
Mass Spectrum - OleoylSHT_28092022_0_K5_000010.d: +MS
Intens.
x10^7
+MS
708.45221
709.45590
710.45928
715.12513
716.12893
N-Oleoyl Serotonin
C28H44N2O2 + FMP10:
C48H58N3O2
M+ : 708.4523
C48H58N3O2, 708.45235
708.45235
709.45562
710.45882
711.46197
ISOTOPE PATTERN SIMULATION
N-Oleoyl Serotonin
C28H44N2O2 + FMP10: C48H58N3O2
M+ : 708.4523
IS: N-Oleoyl Serotonin-d17
C28H27D17N2O2 + FMP10:
C48H41D17N3O2
M+ : 725.5590
725.55323
726.55801
727.56169
724.54864
715.12030
716.12506
707.5
710.0
712.5
715.0
717.5
720.0
722.5
725.0
727.5
730.0
m/z
Individual Axes
List Windows: 3
SmartFormula Manually
Lower formula: C39
Upper formula: N3O2
C 39-n, N 0-3, O 0-2
Note: for m < 2000 the elements C, H, N, and O are considered implicitly.
Generate
Help
Adducts, pos. M
Adducts, neg.
Collect adducts
Measured m/z 708.45221
Tolerance: 2 ppm
Charge: 1
Meas. m/z | # | Ion Formula | Score | m/z | err [ppm] | Mean err [ppm] | mSigma
708.45221 | 1 | C48H58N3O2 | 100.00 | 708.45235 | 0.20 | -0.43 | 13.
Automatically locate monoisotopic peak
Maximum number of formulae 500
Check rings plus double bonds
Minimum -0.5
Maximum 40
Electron configuration even
Filter H/C element ratio
Minimum H/C: 0
Maximum H/C: 3
Estimate carbon number
Generate immediately
Copy to SmartFormula Parameters
Show Pattern
